# RNA sensing via the RIG‐I‐like receptor LGP2 is essential for the induction of a type I IFN response in ADAR1 deficiency

**DOI:** 10.15252/embj.2021109760

**Published:** 2022-02-14

**Authors:** Jorn E Stok, Timo Oosenbrug, Laurens R ter Haar, Dennis Gravekamp, Christian P Bromley, Santiago Zelenay, Caetano Reis e Sousa, Annemarthe G van der Veen

**Affiliations:** ^1^ Department of Immunology Leiden University Medical Centre Leiden The Netherlands; ^2^ Cancer Research UK Manchester Institute The University of Manchester Alderley Park UK; ^3^ Immunobiology Laboratory The Francis Crick Institute London UK

**Keywords:** autoinflammation, innate immunity, RIG‐I‐like receptor family, RNA editing, type I interferon, Microbiology, Virology & Host Pathogen Interaction, RNA Biology

## Abstract

RNA editing by the adenosine deaminase ADAR1 prevents innate immune responses to endogenous RNAs. In ADAR1‐deficient cells, unedited self RNAs form base‐paired structures that resemble viral RNAs and inadvertently activate the cytosolic RIG‐I‐like receptor (RLR) MDA5, leading to an antiviral type I interferon (IFN) response. Mutations in ADAR1 cause Aicardi‐Goutières Syndrome (AGS), an autoinflammatory syndrome characterized by chronic type I IFN production. Conversely, ADAR1 loss and the consequent type I IFN production restricts tumor growth and potentiates the activity of some chemotherapeutics. Here, we show that another RIG‐I‐like receptor, LGP2, also has an essential role in the induction of a type I IFN response in ADAR1‐deficient human cells. This requires the canonical function of LGP2 as an RNA sensor and facilitator of MDA5‐dependent signaling. Furthermore, we show that the sensitivity of tumor cells to ADAR1 loss requires LGP2 expression. Finally, type I IFN induction in tumor cells depleted of ADAR1 and treated with some chemotherapeutics fully depends on LGP2 expression. These findings highlight a central role for LGP2 in self RNA sensing with important clinical implications.

## Graphical abstract

The RNA helicase LGP2 senses endogenous RNAs produced upon loss of the RNA editing enzyme ADAR1 to restrict tumor growth via upregulation of type I IFN production.

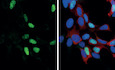

## Introduction

Receptors of the innate immune system continuously sample the intra‐ and extracellular environment for signs of an ongoing infection. Viral infections can be detected through the presence of viral nucleic acids in the cytosol of infected cells (Goubau *et al*, 2013; Rehwinkel & Gack, 2020). Upon encountering viral DNA or RNA, cytosolic nucleic acid sensors, most notably cGAS or RIG‐I‐like receptors (RLRs), respectively, initiate an antiviral type I interferon (IFN) response (Ablasser & Hur, 2020; Rehwinkel & Gack, 2020). While a type I IFN response is important for defense against viral infections, its inadvertent activation by self‐derived nucleic acids induces a sterile inflammatory response that causes immunopathology (Schlee & Hartmann, 2016). Cellular mechanisms that ensure the discrimination between foreign and endogenous nucleic acids are therefore critical to avoid autoinflammation (Schlee & Hartmann, 2016).

RNA modification by the enzyme Adenosine Deaminase Acting on RNA (ADAR1) constitutes an important mechanism by which cells ensure self/nonself RNA discrimination (Heraud‐Farlow & Walkley, 2016; Uggenti *et al*, 2019; Quin *et al*, 2021). Through modification of endogenous RNA, ADAR1 prevents the activation of cytosolic RNA sensors, including RLRs, by cellular RNA molecules and the unwanted induction of an antiviral type I IFN response (Heraud‐Farlow & Walkley, 2016; Uggenti *et al*, 2019; Quin *et al*, 2021). The importance of ADAR1 is highlighted by the severe consequences of *ADAR1* mutations in patients with Aicardi‐Goutières Syndrome (AGS) (Rice *et al*, 2012; Rodero & Crow, 2016). This rare genetic disorder belongs to the spectrum of type I interferonopathies, which are characterized by the constitutive induction of an antiviral type I IFN response in the absence of an infection (Rodero & Crow, 2016). The autoinflammatory condition that arises from inherited *ADAR1* mutations leads to severe (neuro)pathological features (Livingston & Crow, 2016; Rice *et al*, 2017). Notably, ADAR1 has also emerged as an attractive target for novel immunotherapeutic approaches in cancer (Bhate *et al*, 2019). A subset of tumor cells is sensitive to growth arrest upon knockdown or knockout of ADAR1, both *in vivo* and *in vitro* (Gannon *et al*, 2018; Ishizuka *et al*, 2019; Liu *et al*, 2019). In addition, intratumoral loss of ADAR1 increases sensitivity to treatment with immune checkpoint inhibitors and overcomes resistance to such inhibitors *in vivo* (Ishizuka *et al*, 2019). Finally, depletion of ADAR1 in cancer cells potentiates the efficacy of epigenetic therapy and increases type I IFN induction (Mehdipour *et al*, 2020). Understanding the precise mechanism by which ADAR1 (dys)function impacts on innate immunity is therefore essential to better understand its disease‐causing role in interferonopathies as well as its therapeutic potential in cancer.

ADAR1 exists as two isoforms. The nuclear p110 isoform is constitutively expressed, while the p150 isoform is induced by type I IFN receptor signaling and resides primarily in the cytoplasm (Heraud‐Farlow & Walkley, 2016; Quin *et al*, 2021). Both isoforms act on base‐paired RNA to deaminate adenosines and convert them to inosines. A‐to‐I editing is among the most widespread base modifications in mammals. Besides site‐specific A‐to‐I editing, which can alter open reading frames, miRNA seed sequences or RNA splice sites, there is also highly promiscuous and abundant editing of base‐paired RNAs with long regions of high complementarity such as transcripts spanning inverted repeat Alu (IR‐Alu) elements (Eisenberg & Levanon, 2018). Without editing, such base‐paired structures would resemble double‐stranded RNAs (dsRNAs) that are abundantly found in cells infected with some viruses. Unedited self RNA molecules are therefore prone to activate antiviral innate immune mechanisms, such as protein kinase R (PKR) (Chung *et al*, 2018), OAS1/RNase L (Li *et al*, 2017), and the RLR pathway (Mannion *et al*, 2014; Liddicoat *et al*, 2015; Pestal *et al*, 2015). While activation of PKR and OAS/RNase L causes translational shutdown and cell death, RLR engagement initiates the type I IFN response.

The link between ADAR1 editing and RLR activation was first demonstrated in a series of mouse studies. In mice, genetic loss of ADAR1 p110 and p150, p150 alone, or knock‐in of an editing‐deficient ADAR1 mutant (*Adar^E861A^* ^/^ *^E861A^*) results in embryonic lethality, fetal liver disintegration, hematopoiesis defects, and an elevated type I IFN signature (Hartner *et al*, 2004; Wang *et al*, 2004; Ward *et al*, 2011; Liddicoat *et al*, 2015). The embryonic lethality of ADAR1 null or editing‐deficient mice can be rescued by the concurrent deletion of the RLR family member melanoma differentiation‐associated protein 5 (MDA5) or the downstream signaling hub MAVS (mitochondrial antiviral signaling, also known as VISA, Cardif, IPS‐1), but not another RLR, retinoic‐acid‐inducible gene I (RIG‐I) (Mannion *et al*, 2014; Liddicoat *et al*, 2015; Pestal *et al*, 2015; Heraud‐Farlow & Walkley, 2016). In addition, loss of MDA5 or MAVS also eliminates the type I IFN signature in these mice. These observations indicate that unedited RNA mediates its immunostimulatory effects via MDA5 and MAVS and that the type I IFN response plays an important role in the immunopathology caused by loss of ADAR1.

MDA5 normally detects RNA from certain viral species, such as *Picornaviridae* (Dias Junior *et al*, 2019). It senses long stretches of dsRNA or base‐paired single‐stranded RNA, on which it oligomerizes to form filamentous structures (Dias Junior *et al*, 2019; Rehwinkel & Gack, 2020). In contrast, RIG‐I is activated by 5′ di‐ or triphosphate moieties at the base‐paired extremities of certain viral RNA species (Goubau *et al*, 2013; Rehwinkel & Gack, 2020). Activation of RIG‐I or MDA5 by their respective RNA substrates leads to conformational changes that allow their N‐terminal CARD domains to interact with the CARD domains of the adaptor MAVS (Sohn & Hur, 2016). This, in turn, leads to MAVS activation and subsequent phosphorylation and activation of the transcription factors IRF3 and NF‐κB, which mediate the transcription of type I IFNs (most notably IFN‐α subtypes and IFN‐β), type III IFNs, and other pro‐inflammatory cytokines (Goubau *et al*, 2013; Rehwinkel & Gack, 2020). Upon secretion, type I IFNs activate the IFN‐α/β receptor (IFNAR) and induce JAK‐STAT signaling, which results in the transcriptional upregulation of hundreds of IFN‐stimulated genes (ISGs), which establish an antiviral state (Schoggins *et al*, 2011; Schneider *et al*, 2014). Laboratory of genetics and physiology 2 (LGP2) is the third and least well‐understood member of the RLR family. LGP2 lacks the N‐terminal CARD domains and is therefore not able to signal via MAVS (Rodriguez *et al*, 2014; Rehwinkel & Gack, 2020). Instead, LGP2 modulates the function of RIG‐I and MDA5 during viral infection. While LGP2 suppresses RIG‐I signaling, it synergizes with MDA5 to potentiate the sensing of certain RNA viruses (Rodriguez *et al*, 2014; Rehwinkel & Gack, 2020). Akin to MDA5‐deficient mice, LGP2‐knockout mice display increased sensitivity to infection with encephalomyocarditis virus (EMCV), a member of the *Picornaviridae* family (Venkataraman *et al*, 2007; Satoh *et al*, 2010). Mechanistically, LGP2 is incorporated into MDA5 filaments and enhances the interaction between MDA5 and RNA, thereby increasing the rate of MDA5 filament formation (Bruns *et al*, 2014; Duic *et al*, 2020). Simultaneously, LGP2 enhances the dissociation of MDA5 filaments in an ATP‐dependent manner and generates shorter filaments that have greater agonistic activity than longer filaments (Bruns *et al*, 2014; Duic *et al*, 2020). Structural studies demonstrated that LGP2 primarily binds the ends of dsRNA, although it can also coat dsRNA in a similar fashion as MDA5 (Uchikawa *et al*, 2016). Thus, LGP2 promotes rapid MDA5‐dsRNA filament formation yet yields shorter filaments, ultimately leading to enhanced downstream signaling and an increased type I IFN response.

LGP2 also impacts on type I IFN responses through alternative routes that are independent from its role as typical RNA sensor. Wild‐type LGP2 and mutants that fail to hydrolyze ATP or bind RNA interact with MAVS at steady state and block the interaction between RIG‐I and MAVS, thereby limiting RIG‐I‐mediated MAVS activation (Esser‐Nobis *et al*, 2020). Upon stimulation with the dsRNA mimic poly(I:C), LGP2 releases MAVS for interaction with RIG‐I (Esser‐Nobis *et al*, 2020). LGP2 additionally limits RIG‐I signaling and potentiates MDA5 signaling by a direct protein–protein interaction with the dsRNA‐binding protein PACT (Sanchez David *et al*, 2019). Furthermore, LGP2 inhibits Dicer‐mediated processing of dsRNA (Van der Veen *et al*, 2018), perhaps to preserve dsRNA substrates for the full‐blown activation of the type I IFN response. Conversely, LGP2 may negatively regulate the antiviral type I IFN response by associating and interfering with the function of TRAF ubiquitin ligases, in a manner that is independent of ATP hydrolysis or RNA binding (Parisien *et al*, 2018). Finally, LGP2 controls CD8^+^ T cell survival and fitness during West Nile virus and lymphocytic choriomeningitis virus infection in mice, pointing to cell type‐specific functions (Suthar *et al*, 2012).

Both MDA5 and RIG‐I can bind and be activated by endogenous RNA in various contexts (Dias Junior *et al*, 2019; Streicher & Jouvenet, 2019; Stok *et al*, 2020). LGP2 has predominantly been studied upon viral infection or mimics thereof. A recent study demonstrated that mice bearing a mutation in the Zα domain of ADAR1 that is involved in binding to dsRNA in its unusual Z‐conformation (Z‐RNA) suffer from postnatal growth retardation and mortality and have a mild type I IFN signature, which can be reverted by crossing these mice with MDA5, MAVS, PKR, as well as LGP2‐knockout mice (Maurano *et al*, 2021). The extent to which LGP2 is required for type I IFN induction in response to unedited RNA species more broadly (aside from Z‐RNA), the molecular mechanism that is involved, and whether it is required in humans is unclear.

Here, we investigated the role of human LGP2 in induction of type I IFNs caused by ADAR1 deficiency. Using various genetic approaches and model systems, we demonstrate that LGP2 is essential for this induction, in a manner that involves its classical function as RNA sensor. Importantly, we further demonstrate that LGP2 is required both for sensing of unedited RNA and for reduced cell growth upon loss of ADAR1 in tumor cells. Finally, treatment of ADAR1‐depleted tumor cells with epigenetic repressors, a promising strategy for cancer therapy, potentiates the type I IFN response in an LGP2‐dependent manner. Our findings provide molecular insight into the effector mechanisms that are engaged upon dysregulation of ADAR1, with important clinical implications for the field of interferonopathies as well as cancer.

## Results

### Human LGP2 is required for the induction of a type I IFN response upon depletion of ADAR1

**Figure 1:**
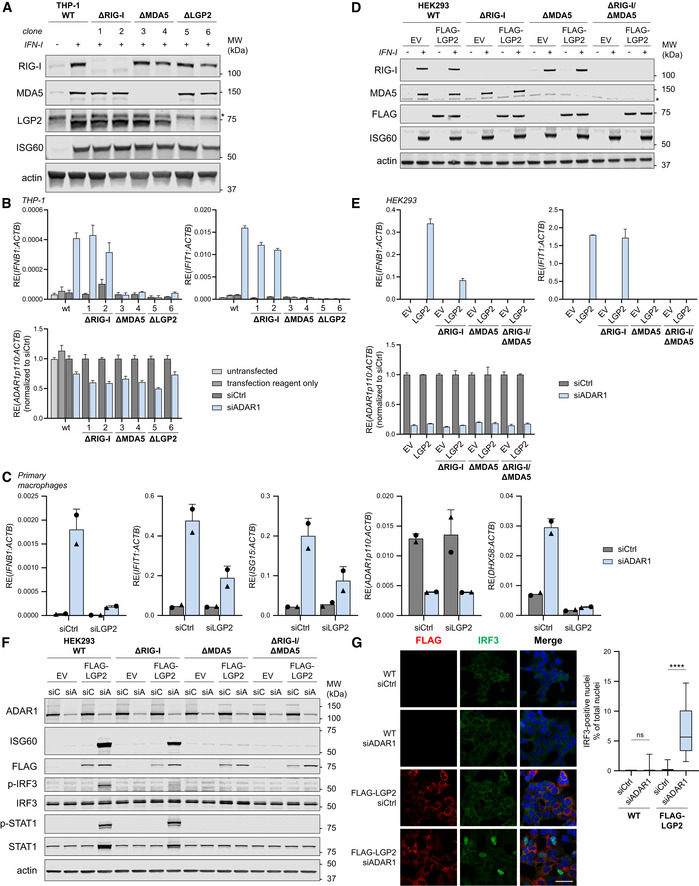
Human LGP2 is essential for the induction of a type I IFN response upon depletion of ADAR1 THP‐1 monocytes were genetically engineered to knockout RIG‐I, MDA5, or LGP2 using CRISPR/Cas9. Cells were differentiated toward macrophage‐like cells using PMA and treated with recombinant type I IFN to upregulate RLR expression. Correct gene editing and intact type I IFN responsiveness were validated by SDS‐PAGE and immunoblotting using the indicated antibodies (*n* = 3). *, nonspecific band.Cells generated in (A) were differentiated using PMA and transfected with a control siRNA (siCtrl) or an ADAR1‐targeting siRNA (siADAR1). The type I IFN response was monitored 56 h post‐transfection by RT‐qPCR analysis to determine IFN‐β and IFIT1 transcript expression, normalized to a housekeeping gene (ACTB). ADAR1 knockdown efficiency was monitored by ADAR1 p110 expression, normalized to ACTB, and displayed relative to siCtrl. Data are means ± s.d. from a representative of three biological replicate experiments.Primary human monocyte‐derived macrophages were transfected with the indicated siRNAs. Cells were harvested 96 h post‐transfection and RT‐qPCR analysis was used to monitor the type I IFN response (IFN‐β, IFIT1, and ISG15 transcripts) and knockdown efficiency of ADAR1 and LGP2 (*DHX58*). All transcripts were normalized to ACTB. Data from two independent donors (denoted with distinct symbols) are shown with mean ± s.d.HEK293 cells were genetically engineered to knockout RIG‐I, MDA5, or both, and subsequently subjected to retroviral transduction to stably express FLAG‐LGP2 or an empty vector (EV). Correct gene editing and intact type I IFN responsiveness were validated by SDS‐PAGE and immunoblotting using the indicated antibodies (*n* = 2). *, nonspecific band.Cells generated in (D) were transfected with siCtrl or siADAR1. The type I IFN response and ADAR1 knockdown efficiency were monitored 78 h post‐transfection as in (B). Data are means ± s.d. from a representative of four biological replicate experiments.Cells generated in (D) were transfected with siCtrl (siC) or siADAR1 (siA). Protein lysates were prepared 78 h post‐transfection, followed by SDS‐PAGE and immunoblotting using the indicated antibodies (*n* = 2).HEK293 WT cells and FLAG‐LGP2‐expressing HEK293 cells were transfected with siCtrl or siADAR1 and subsequently plated on coverslips for immunofluorescence microscopy. Cells were fixed, permeabilized, and stained 72 h post‐transfection with anti‐FLAG (red) and anti‐IRF3 (green) antibodies. Nuclei were stained with DAPI (blue). Scale bar is 50 μm. Total nuclei (> 450 nuclei per experimental condition) and IRF3‐positive nuclei were counted using semi‐automated software analysis and plotted as percentage IRF3‐positive nuclei of total nuclei per field of view (a representative of three biological replicate experiments is quantified). The boxplot indicates the interquartile range as a box, the median as a central line, and the whiskers extend from the minimum to the maximum value. Statistical analyses were performed using unpaired two‐tailed Mann–Whitney *U* tests. ns, not significant; ****P < 0.0001. Source data are available online for this figure.

**Figure EV1:**
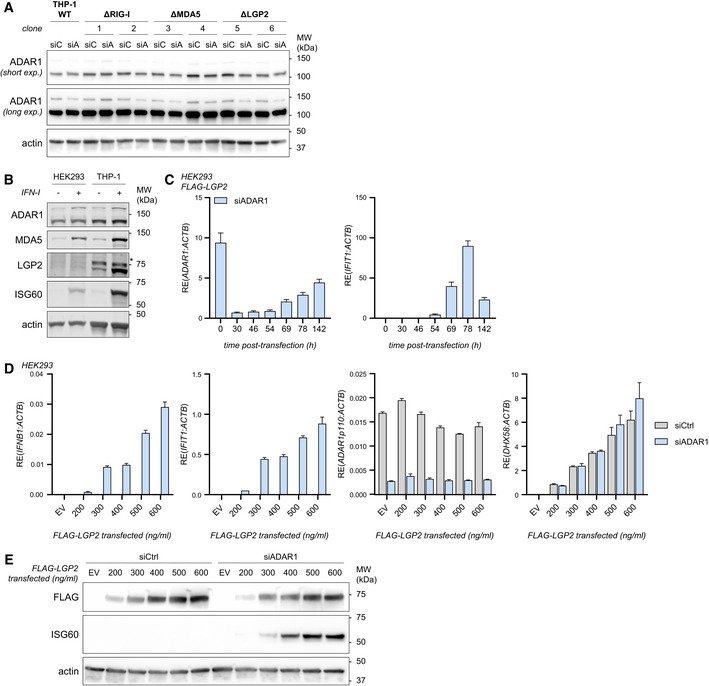
LGP2 is required for the induction of a type I IFN response upon siRNA‐mediated depletion of ADAR1 in HEK293, related to Fig 1 PMA‐differentiated THP‐1 WT, RIG‐I‐, MDA5‐, and LGP2‐knockout cells were transfected with siCtrl (siC) or siADAR1 (siA). Protein lysates were prepared 56 h post‐transfection and ADAR1 knockdown efficiency was monitored by immunoblot analysis. Data correspond to the biological replicate shown in Fig 1B.Expression level and type I IFN inducibility of relevant proteins in HEK293 and THP‐1. HEK293 and PMA‐differentiated THP‐1 cells were treated with or without recombinant type I IFN. Protein lysates were analyzed by SDS‐PAGE followed by immunoblotting with the indicated antibodies (*n* = 3). *, nonspecific band.Kinetics of siRNA‐mediated depletion of ADAR1 and induction of the type I IFN response in HEK293. HEK293 cells stably expressing FLAG‐LGP2 were transfected with an siRNA targeting ADAR1 (siADAR1) and harvested at the indicated time points post‐transfection. ADAR1 knockdown and IFIT1 upregulation were monitored by RT‐qPCR analysis (using Taqman probes) and normalized to ACTB. Data are means ± s.d. from one experiment.HEK293 WT cells were transfected with siADAR1 or a control siRNA (siCtrl) and 8 h later with increasing amounts of a vector encoding FLAG‐LGP2. As a control, cells were transfected with 250 ng of an empty vector (EV). Cells were harvested 80 h post siRNA transfection. RT‐qPCR analysis was used to monitor IFN‐β and IFIT1 expression, ADAR1 knockdown, and LGP2 (*DHX58*) expression. All transcripts were normalized to ACTB. Data are means ± s.d. from a representative of two biological replicate experiments.Cells were treated as in (D). Protein lysates were prepared and analyzed by SDS‐PAGE followed by immunoblotting using the indicated antibodies. Source data are available online for this figure.

To investigate the role of human RLRs in the induction of type I IFN caused by the absence of ADAR1, we first knocked out RIG‐I, MDA5, or LGP2 in the human monocytic leukemia cell line THP‐1 using CRISPR/Cas9‐mediated genome engineering. Correct gene ablation was confirmed by immunoblotting cells treated with recombinant type I IFN to upregulate the expression of RIG‐I, MDA5, and LGP2, which are encoded by ISGs themselves (Fig 1A). Intact type I IFN receptor signaling was verified by monitoring ISG60 upregulation (Fig 1A). For each RLR, two knockout clones were differentiated into macrophages and transfected with siRNAs targeting both isoforms of ADAR1. Despite the modest efficiency of the knockdown at the time point chosen for analysis (Figs 1B and EV1A), we observed a clear upregulation of transcripts encoding IFN‐β and the ISG IFIT1 in wild‐type cells, indicative of type I IFN induction (Fig 1B). Notably, loss of LGP2 completely abrogated type I IFN induction and signaling upon ADAR1 depletion (Fig 1B). Loss of MDA5, but not RIG‐I, also interfered with the type I IFN response, consistent with published literature (Heraud‐Farlow & Walkley, 2016). Note that throughout the manuscript ADAR1 knockdown efficiency is monitored through measurement of p110 expression levels, as analysis of the p150 isoform underestimates knockdown efficiency due to its IFN‐inducible nature. Consistent with these observations, siRNA‐mediated depletion of ADAR1 in primary human monocyte‐derived macrophages induced a type I IFN response (monitored by IFN‐β, IFIT1, and ISG15 transcript levels), which was markedly reduced upon co‐depletion of LGP2 (Fig 1C). Together, these data indicate that, besides MDA5, expression of LGP2 is crucial for the induction of a type I IFN response in ADAR1 deficiency.

**Figure EV2:**
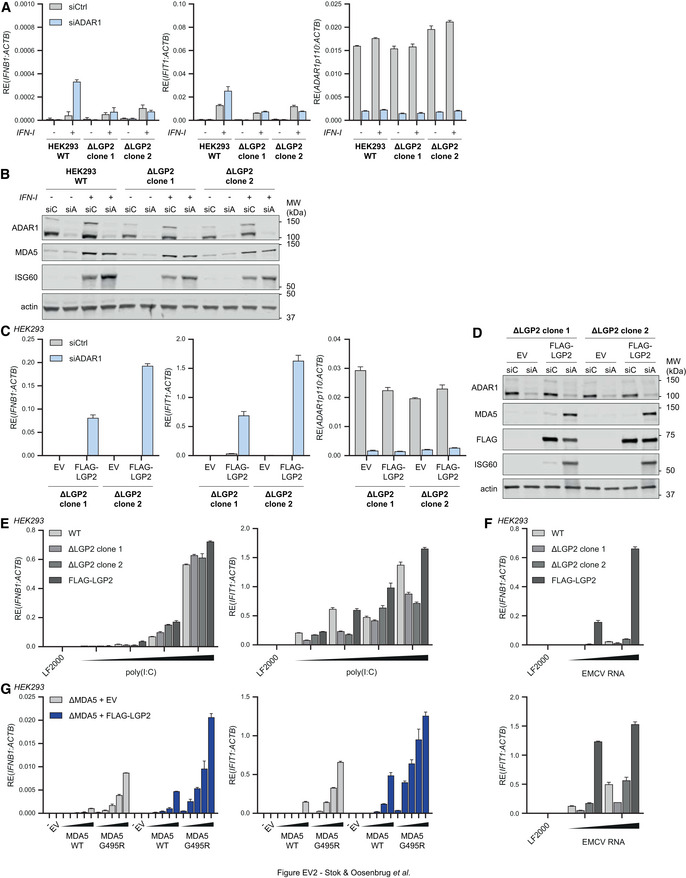
LGP2‐deficient cells fail to sense unedited self RNAs, yet maintain the ability to detect viral dsRNAs, related to Fig 1 AWT and LGP2‐knockout (clones 1 and 2) HEK293 cells were transfected with an siRNA targeting ADAR1 (siADAR1) or a control siRNA (siCtrl) and were treated 8 h later with recombinant type I IFN to upregulate RLR expression. Cells were harvested 80 h post siRNA transfection and RT‐qPCR analysis was used to monitor IFN‐β and IFIT1 expression and ADAR1 knockdown. All transcripts were normalized to ACTB. Data are means ± s.d. from a representative of three biological replicate experiments.BCells were treated as in (A). Protein lysates were prepared 80 h post siRNA transfection, followed by SDS‐PAGE and immunoblotting using the indicated antibodies (*n* = 3). siC, siCtrl; siA, siADAR1.CLGP2‐knockout (clones 1 and 2) HEK293 cells were transfected with siADAR1 or siCtrl and 8 h later with a vector encoding FLAG‐LGP2 or an empty vector (EV). Cells were harvested 80 h post siRNA transfection and RT‐qPCR analysis was used to monitor IFN‐β and IFIT1 expression and ADAR1 knockdown. All transcripts were normalized to ACTB. Data are means ± s.d. from a representative of two biological replicate experiments.DCells were treated as in (C). Protein lysates were prepared 80 h post siRNA transfection, followed by SDS‐PAGE and immunoblotting using the indicated antibodies (*n* = 2).E, FWT, LGP2‐knockout (clones 1 and 2), and stably expressing FLAG‐LGP2 HEK293 cells were transfected with transfection reagent only (LF2000), poly(I:C) (56, 112, 225, or 450 ng in (E)), or RNA isolated from HEK293 cells infected with EMCV in the presence of ribavirin (450 or 900 ng in (F)). Cells were harvested 16 h post‐transfection and RT‐qPCR analysis was used to monitor IFN‐β and IFIT1 expression. All transcripts were normalized to ACTB. Data are means ± s.d. from a representative of four (E) or three (F) biological replicate experiments.GMDA5‐knockout HEK293 cells stably expressing FLAG‐LGP2 or an empty vector (EV) were transfected with increasing amounts (5, 20, 40, 80, or 240 ng/ml) of a vector encoding FLAG‐MDA5 WT or FLAG‐MDA5 G495R. As a control, cells were transfected with 240 ng/ml control vector or left untreated. Cells were harvested 24 h post‐transfection and RT‐qPCR analysis was used to monitor IFN‐β and IFIT1 expression. All transcripts were normalized to ACTB. Data are means ± s.d. from a representative of two biological replicate experiments. Source data are available online for this figure.

To further delineate the contribution of LGP2 to the sensing of unedited self RNA, we knocked out RIG‐I, MDA5, or both by CRISPR/Cas9‐mediated gene editing in the human cell line HEK293. Correct gene editing was confirmed by immunoblotting and intact type I IFN receptor signaling in the selected clones was verified by monitoring ISG60 expression upon recombinant type I IFN treatment (Fig 1D). Unexpectedly, siRNA‐mediated depletion of ADAR1 did not yield signs of a type I IFN response in parental HEK293 cells or its CRISPR/Cas9‐engineered derivatives (Fig 1E). We noted that these HEK293 cells expressed nearly undetectable levels of LGP2, even after stimulation with recombinant type I IFN (Fig EV1B). Importantly, ectopic expression of LGP2 by means of retroviral transduction and stable integration of a FLAG‐tagged LGP2‐encoding vector (Fig 1D) enabled type I IFN induction upon siRNA‐mediated ADAR1 depletion, as determined by the expression of IFN‐β and IFIT1 transcripts (Fig 1E). This was evident in MDA5‐sufficient but not in MDA5‐deficient cells, confirming that LGP2 and MDA5 were both necessary. As expected, loss of RIG‐I did not have a major impact on type I IFN induction upon ADAR1 knockdown (Fig 1E). Of note, increased MDA5 expression, through pretreatment with recombinant type I IFN, did not bypass the requirement for LGP2 (Fig EV2A and B), which suggests that the level of MDA5 is not the rate‐limiting factor. The ISG signature reached its maximum around 78 h post‐siRNA delivery in LGP2‐overexpressing cells (Fig EV1C). These observations were confirmed at protein level: ADAR1 depletion upon siRNA treatment led to robust upregulation of the protein ISG60 exclusively in cells that express both MDA5 and LGP2 (Fig 1F). Moreover, the presence of both LGP2 and MDA5 was required for phosphorylation of IRF3 and STAT1, two key transcription factors that act downstream of MAVS and IFNAR to induce IFN‐β and ISG transcription, respectively (Fig 1F). Finally, nuclear translocation of IRF3, a hallmark of type I IFN induction, only occurred upon expression of LGP2 in ADAR1‐depleted cells (Fig 1G). These observations demonstrate that LGP2 is essential for type I IFN induction and signaling upon ADAR1 depletion.

Previous studies indicated that LGP2 can function as a concentration‐dependent biphasic switch that favors MDA5 signaling in response to viral ligands at low concentrations while inhibiting MDA5‐dependent responses at high concentrations (Rodriguez *et al*, 2014). However, in our experiments, increasing amounts of an LGP2‐encoding plasmid led to a gradual increase in the type I IFN response upon siRNA‐mediated ADAR1 depletion without any signs of an inhibitory effect (Fig EV1D and E) except at very high doses of LGP2, which negatively affect cell viability. The LGP2‐dependent biphasic response previously reported in the context of viral dsRNA sensing is therefore not evident in self RNA sensing.

The absolute requirement for LGP2 in the induction of a type I IFN response following ADAR1 depletion was surprising and distinct from its role in viral dsRNA sensing, where LGP2 evidently potentiates MDA5 signaling but is not strictly required. Indeed, while bona fide LGP2‐knockout HEK293 cells failed to induce a type I IFN response upon ADAR1 depletion (Fig EV2A and B), they retained the ability to induce a modest, yet reduced, type I IFN response upon stimulation with the dsRNA mimic high molecular weight (HMW) poly(I:C) or RNA isolated from EMCV‐infected cells, both of which activate MDA5 (Fig EV2E and F). As a control, the siADAR1‐induced IFN response was restored in LGP2‐knockout cells upon ectopic LGP2 expression (Fig EV2C and D). Whether the differential detection of unedited self RNA versus viral RNA by LGP2/MDA5 is caused by a qualitative or quantitative difference, or both, is not clear. Unedited self RNA may either be less abundant in cells or be a less suitable MDA5 ligand (e.g., because it contains only short stretches of base‐paired regions as opposed to long dsRNA found in viral RNA) and therefore it may be more reliant on LGP2 for its detection. Either way, it is evident that the requirement for LGP2 becomes critical in the case of an “imperfect” MDA5 ligand. As a side note, in another setting of autoinflammation due to a gain‐of‐function mutation in MDA5 (MDA5 G495R) (Rice *et al*, 2014), LGP2 also enhanced but was not strictly required for type I IFN induction (Fig EV2G), which indicates that LGP2 is not necessarily essential for the detection of all types of self RNA. Altogether, these findings implicate human LGP2 as a key player in the response to unedited self RNA in ADAR1‐depleted cells.

### Sensing of unedited self RNA via LGP2 requires RNA binding and ATP hydrolysis

**Figure 2:**
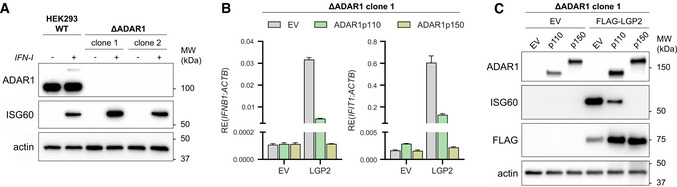
A type I IFN response is unleashed in ADAR1‐knockout cells upon expression of LGP2 HEK293 cells were genetically engineered to knock out ADAR1 using CRISPR/Cas9. Cells were treated for 24 h with recombinant type I IFN to upregulate ADAR1 p150 and ISG60 to confirm correct gene editing and type I IFN responsiveness, respectively. Protein lysates were analyzed by SDS‐PAGE followed by immunoblotting using the indicated antibodies (*n* = 3).ADAR1‐knockout HEK293 cells (clone 1) were cotransfected with an empty vector (EV) or a FLAG‐LGP2‐encoding vector (LGP2) combined with a vector encoding GFP‐tagged ADAR1 p110 or p150. Cells were harvested 72 h post‐transfection and the type I IFN response was monitored by RT‐qPCR analysis of IFN‐β and IFIT1 expression, normalized to ACTB. Data are means ± s.d. from a representative of four biological replicate experiments.ADAR1‐knockout cells (clone 1) were transfected as in (B). Protein lysates were analyzed by SDS‐PAGE followed by immunoblotting using the indicated antibodies (*n* = 4). Source data are available online for this figure.

**Figure EV3:**
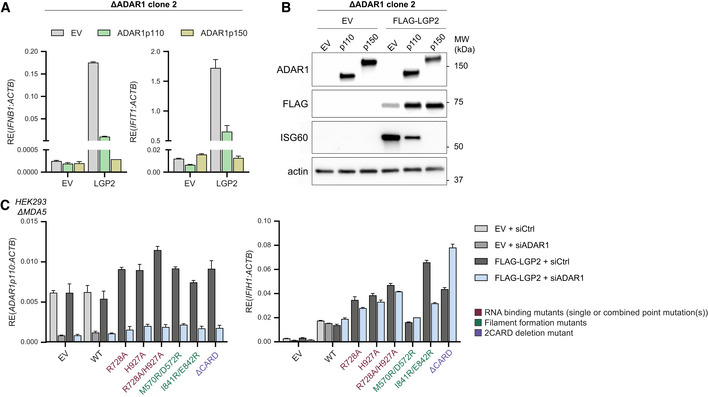
A type I IFN response is unleashed in ADAR1‐knockout cells upon expression of LGP2, related to Figs 2 and 3 ADAR1‐knockout HEK293 cells (clone 2) were cotransfected with an empty vector (EV) or a FLAG‐LGP2‐encoding vector (LGP2) combined with a vector encoding GFP‐tagged ADAR1 p110 or p150. Cells were harvested 48 h post‐transfection and the type I IFN response was monitored by measuring IFN‐β and IFIT1 transcript expression, relative to ACTB expression, by RT‐qPCR. Data are means ± s.d. from a representative of three biological replicate experiments.ADAR1‐knockout HEK293 cells (clone 2) were transfected as in (A). Protein lysates were analyzed by SDS‐PAGE followed by immunoblotting using the indicated antibodies (*n* = 3).MDA5‐knockout HEK293 cells, generated in Fig 1D, were transfected with an ADAR1‐targeting siRNA (siADAR1) or a control siRNA (siCtrl) and 8 h later with an empty vector (EV) or a vector encoding the indicated WT, truncation, or point mutant(s) of MDA5. Cells were harvested 72 h post‐siRNA transfection and RT‐qPCR analysis was used to monitor ADAR1 knockdown and MDA5 (*IFIH1*) expression. All transcripts were normalized to ACTB. Data are means ± s.d. from a representative of two biological replicate experiments. Source data are available online for this figure.

The limited expression of LGP2 and the absence of a type I IFN response upon ADAR1 depletion in wild‐type HEK293 cells allowed us to create ADAR1 knockout cells through CRISPR/Cas9, without the activation of innate immune pathways that hinder cell proliferation. Two ADAR1‐knockout clones were selected that completely lost expression of the ADAR1 p110 and p150 isoform yet remained responsive to type I IFNs, as determined by immunoblotting (Fig 2A). Genetic loss of ADAR1 did not reveal a type I IFN response until introduction of LGP2 (Figs 2B and C, and EV3A and B), in line with our earlier observations using ADAR1 siRNAs. As reported (Pestal *et al*, 2015), the IFN response was largely due to the loss of the p150 isoform, as reconstitution of p150 expression completely blocked type I IFN induction in LGP2‐expressing ADAR1 knockout cells (Figs 2B and C, and EV3A and B). In contrast, overexpression of the p110 isoform reduced, but did not block, this type I IFN response. The reduction can most likely be explained by overexpression of this isoform, which is normally restricted to the nucleus but can “spill” into the cytosol in overexpressing cells. The ADAR1‐deficient cells with a tunable, LGP2‐dependent type I IFN response provide us therefore with a useful tool to dissect the features of LGP2 and its interaction partners that are required for unedited self RNA sensing.

**Figure 3:**
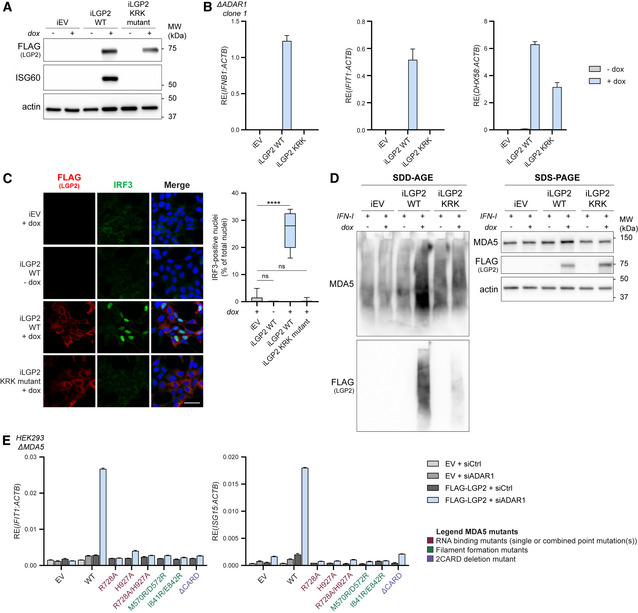
RNA binding by LGP2 is required for receptor oligomerization and type I IFN induction in ADAR1‐knockout cells ADAR1‐knockout HEK293 cells (clone 1) were modified with a lentiviral‐based inducible system to express FLAG‐LGP2 WT or a FLAG‐LGP2 RNA binding mutant (K138E/R490E/K634E, denoted as “KRK mutant”) in a doxycycline‐regulated manner. Cells were treated 72 h with doxycycline (dox). Protein lysates were analyzed by SDS‐PAGE and immunoblotting using the indicated antibodies (*n* = 3). iEV, inducible empty vector; iLGP2, inducible LGP2.Cells generated in (A) were treated with doxycycline for 72 h to induce LGP2 WT or KRK mutant gene expression. The type I IFN response (IFN‐β and IFIT1 transcripts) and LGP2 (*DHX58*) expression were monitored by RT‐qPCR analysis. All transcripts were normalized to ACTB. Data are means ± s.d. from a representative of three biological replicate experiments.Cells generated in (A) were plated on coverslips and treated with or without doxycycline for 72 h. Cells were fixed, permeabilized, and stained with anti‐FLAG (red) and anti‐IRF3 (green) antibodies. Nuclei were stained with DAPI (blue). Scale bar is 50 μm. Total nuclei (> 500 nuclei per experimental condition) and IRF3‐positive nuclei were counted using semi‐automated software analysis and plotted as percentage IRF3‐positive nuclei of total nuclei per field of view (a representative of two biological replicate experiments is quantified). The boxplot indicates the interquartile range as a box, the median as a central line, and the whiskers extend from the minimum to the maximum value. Statistical analysis was performed using a Kruskal–Wallis test with a Dunn's *post hoc* test for multiple comparisons. ns, not significant; ****P < 0.0001.Cells generated in (A) were treated with doxycycline for 72 h. During the last 24 h, recombinant type I IFN was added to upregulate endogenous MDA5 protein expression. Protein lysates were analyzed by SDD‐AGE and SDS‐PAGE using the indicated antibodies to determine protein oligomerization and total expression levels, respectively (*n* = 3).MDA5‐knockout HEK293 cells, generated in Fig 1D, were transfected with an ADAR1‐targeting siRNA (siADAR1) or a control siRNA (siCtrl) and 8 h later with an empty vector (EV) or a vector encoding the indicated WT, truncation, or point mutant(s) of MDA5. Cells were harvested 72 h post‐siRNA transfection and the type I IFN response was monitored by RT‐qPCR analysis of IFIT1 and ISG15 transcript expression, normalized to ACTB. Data are means ± s.d. from a representative of two biological replicate experiments. Source data are available online for this figure.

The canonical function of LGP2 as an RNA sensor involves RNA binding and ATP hydrolysis while other roles, such as interaction with MAVS and TRAFs, do not (Parisien *et al*, 2018; Esser‐Nobis *et al*, 2020). We introduced, by means of lentiviral transduction, a doxycycline‐inducible system to stably express FLAG‐LGP2 WT or a mutant that completely fails to bind RNA (FLAG‐LGP2 K138E/R490E/K634E, denoted as “LGP2 KRK” in figures) in ADAR1 KO cells (Fig 3A and B). Doxycycline‐induced expression of LGP2 WT in ADAR1 KO cells led to robust ISG60 protein (Fig 3A) and IFN‐β and IFIT1 transcript induction (Fig 3B). In contrast, expression of the LGP2 RNA‐binding mutant did not induce a type I IFN response. Consistent with these findings, induction of LGP2 WT, but not the RNA‐binding mutant, allowed nuclear translocation of IRF3 (Fig 3C). These observations indicate that binding to RNA substrates is required for LGP2‐dependent type I IFN induction in ADAR1‐deficient cells.

To determine whether LGP2 is required for MDA5 oligomerization in ADAR1‐deficient cells, we utilized semi‐denaturing detergent agarose gel electrophoresis (SDD‐AGE) to monitor MDA5 aggregation. To circumvent discrepancies in MDA5 protein levels across samples (due to its increased expression as an ISG in LGP2‐expressing ADAR1 KO cells), we treated cells with recombinant type I IFN to equalize MDA5 expression (Fig 3D, SDS‐PAGE). Doxycycline‐inducible expression of LGP2 WT, but not the RNA‐binding mutant, revealed MDA5 aggregation in ADAR1‐knockout cells (Fig 3D, SDD‐AGE). SDD‐AGE further revealed that RNA‐binding competent LGP2 oligomerizes in ADAR1‐knockout cells (Fig 3D), consistent with previous studies showing that human and chicken LGP2 itself can form filaments (Bruns *et al*, 2014; Uchikawa *et al*, 2016). We further tested what features of MDA5 are important for LGP2‐dependent type I IFN induction in ADAR1‐depleted cells. We transiently expressed various MDA5 mutants in MDA5 KO HEK293 cells that were stably transduced with FLAG‐LGP2 or an empty vector (as a control) and depleted ADAR1. In contrast to WT MDA5, MDA5 mutants that have impaired capacity to form filaments (M570R/D572R, I841R/E842R) or to bind RNA (R728A, H927A, R728A/H927A) (Wu *et al*, 2013) or that lack the CARD domains (MDA5ΔCARD), all fail to induce a type I IFN response in ADAR1‐depleted cells (Figs 3E and EV3B). Altogether, the above findings place LGP2 at the level of MDA5 oligomerization and activation in the ADAR1‐induced type I IFN response.

**Figure 4:**
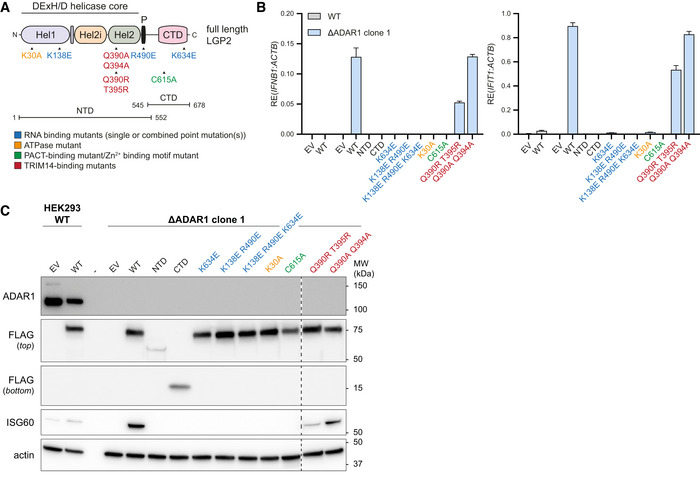
The function of LGP2 in sensing unedited self RNA involves its canonical role as dsRNA sensor that requires RNA binding and ATP hydrolysis Schematic illustration of the domain structure of LGP2 and various point mutants and truncation mutants that are used in this study. The N‐terminal domain (NTD) of LGP2 is composed of a conserved DExH/D helicase domain, subdivided into the helicase 1 (Hel1), helicase 2 (Hel2) and helicase insertion (Hel2i) domain, and a pincer motif (P). The NTD is followed by a C‐terminal domain (CTD), involved in RNA binding.HEK293 WT or ADAR1‐knockout cells (clone 1) were transiently transfected with an empty vector (EV) or a vector encoding the indicated WT, truncation, or point mutant(s) of LGP2. Cells were harvested 72 h post‐transfection and the type I IFN response was monitored by RT‐qPCR analysis of IFN‐β and IFIT1 transcript expression, normalized to ACTB. Data are means ± s.d. from a representative of three biological replicate experiments.Cells were treated as in (B). Protein lysates were prepared and analyzed by SDS‐PAGE followed by immunoblotting using the indicated antibodies (*n* = 3). The dotted line indicates the juxtaposition of two nonadjacent lanes. Source data are available online for this figure.

We transiently expressed various LGP2 truncation mutants and point mutants (Fig 4A) in ADAR1‐knockout cells and observed that, besides RNA binding, full‐length LGP2 and its ability to hydrolyze ATP are strictly required to sustain a type I IFN response. Expression of the LGP2 N‐terminal domain (NTD), C‐terminal domain (CTD), or mutation of LGP2 residues that are critical for ATPase activity (K30A) or RNA binding via the LGP2 NTD (K138E/R490E) or CTD (K634E) (Pippig *et al*, 2009; Bruns *et al*, 2013; Uchikawa *et al*, 2016), all abolished type I IFN induction in ADAR1‐deficient cells (Fig 4B and C).

Mutation of a cysteine residue in the C‐terminal domain of LGP2 crucial for binding to the dsRNA‐binding protein PACT (C615A) (Sanchez David *et al*, 2019) also prevented type I IFN induction, suggesting that PACT, via LGP2, may participate in the IFN response to unedited self RNA (Fig 4B and C). Of note, C615 is also important for the correct orientation of a Zn^2+^ ion in LGP2 (Pippig *et al*, 2009); hence, the role of PACT and/or Zn^2+^ binding will need to be evaluated in further studies.

A recent study identified a biochemical interaction between LGP2 filamentous structures and TRIM14, an unusual member of the TRIM family that lacks a RING domain and does not function as a ubiquitin E3 ligase (Kato *et al*, 2021). Mutations in the α3 helix of the Hel2 domain of LGP2 (Q390R/T395R or Q390A/Q394A) strongly decreased the interaction between the Hel2i‐Hel2 domain of LGP2 and TRIM14 (Kato *et al*, 2021). We found that the Q390A/Q394A mutation did not impact on the ability of LGP2 to induce a type I IFN response in ADAR1 knockout cells, while the Q390R/T395R mutation reduced, but not abolished, type I IFN induction (Fig 4B and C). Whether TRIM14 plays a more pronounced role in type I IFN induction upon picornavirus infection or perhaps regulates alternative, noncanonical functions of LGP2, needs to be further explored in a TRIM14‐deficient setting.

We conclude that, besides RNA binding, ATP hydrolysis is strictly required for LGP2 to mediate type I IFN induction in ADAR1‐deficient cells. This suggests that the function of LGP2 in inflammation in ADAR1 deficiency involves its canonical role as RNA sensor rather than an “indirect” role, for example, via its interaction with MAVS or TRAFs. In addition, the binding of LGP2 to PACT or a Zn^2+^ ion is important for type I IFN induction in ADAR1‐deficient cells.

### LGP2 is required for growth retardation of tumor cells and cell‐intrinsic inflammation upon loss of ADAR1, which is potentiated by epigenetic therapy

**Figure 5:**
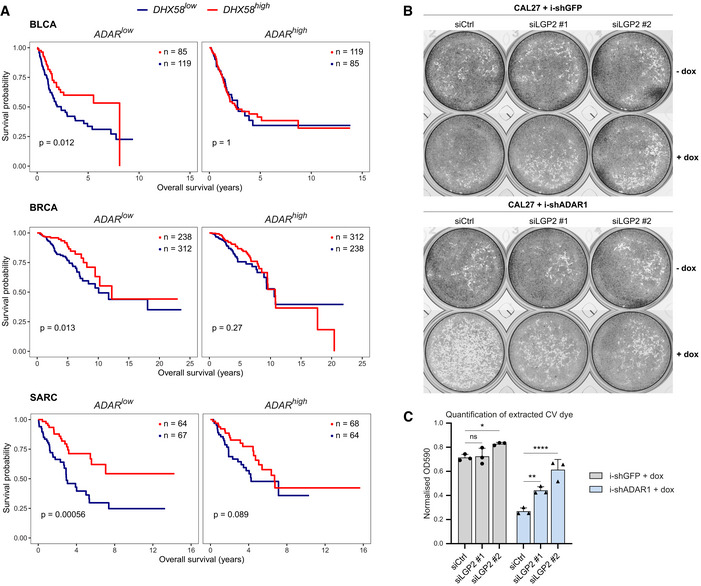
Reduced tumor cell growth upon loss of ADAR1 is dependent on LGP2 Kaplan–Meier plots showing overall survival of *ADAR* ^low^ and *ADAR* ^high^ patients stratified by *DHX58* levels in bladder cancer (BLCA; *n* = 407), breast cancer (BRCA; *n* = 1099), and sarcoma (SARC; *n* = 263) TCGA datasets. Median cut‐offs were used for patient stratification and logrank test *P* values are shown.CAL27 cells transduced with doxycycline‐inducible shRNAs targeting ADAR1 or GFP (negative control) were treated with doxycycline and transfected with two independent siRNAs targeting LGP2 (siLGP2 #1 or #2) or a control siRNA (siCtrl). To determine cell confluency at 120 h post‐transfection, cells were fixed, stained with Crystal Violet, and imaged. Images of a representative experiment are shown (*n* = 3).Crystal Violet was extracted from stained cells (B) and the dye intensity was quantified using a colorimetric assay (OD_590_). OD_590_ values of doxycycline‐treated cells were normalized to the OD_590_ values of untreated cells. Quantification of data from three independent experiments is shown as mean ± s.d. Statistical analysis was performed using ordinary two‐way ANOVA with Tukey's *post hoc* test. ns, not significant; *P < 0.05; **P < 0.01; ****P < 0.0001.

**Figure EV4:**
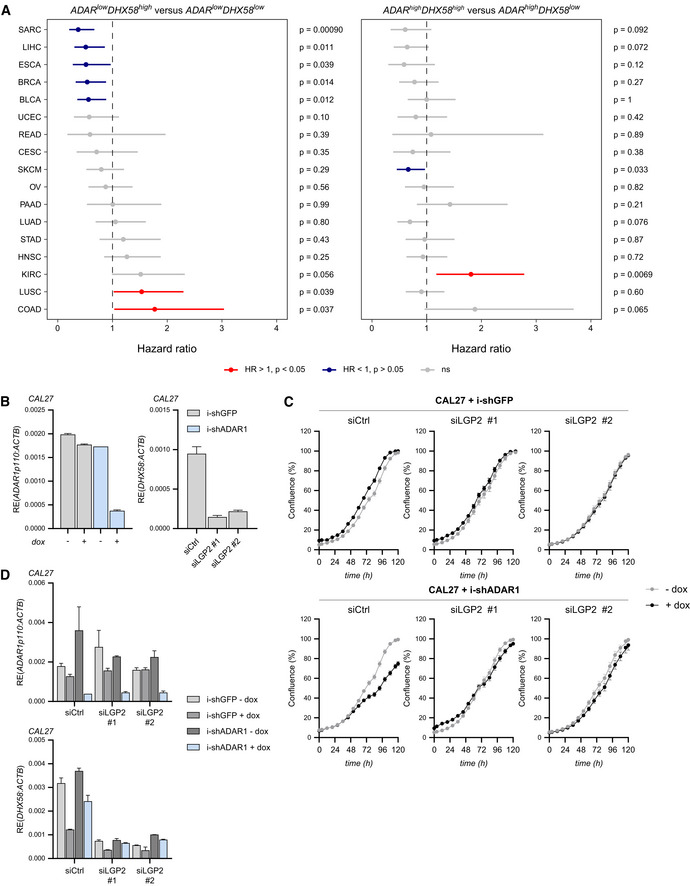
Loss of ADAR1 inhibits tumor cell growth in an LGP2‐dependent manner, related to Fig 5 A
*ADAR* ^low^ patients with concomitant *DHX58* ^high^ expression have prolonged survival across multiple cancer types. Hazard ratios and 95% confidence intervals from univariate Cox regression models for *DHX58* stratification in *ADAR* ^low^ (left panel) and *ADAR* ^high^ (right panel) patients from 17 TCGA datasets (sarcoma = SARC, liver = LIHC, esophageal = ESCA, breast = BRCA, bladder = BLCA, endometrial = UCEC, rectal = READ, cervical = CESC, melanoma = SKCM, ovarian = OV, pancreas = PAAD, lung adenocarcinoma = LUAD, stomach = STAD, head and neck = HNSC, clear cell renal cell carcinoma = KIRC, lung squamous = LUSC, colon = COAD). Median cut‐off values for both *ADAR* and *DHX58* were used for patient stratification. Dashed lines indicate a hazard ratio of 1. Wald test *P* values are shown.B–DCAL27 cells were transduced with doxycycline‐inducible shRNAs targeting ADAR1 or GFP (negative control) and subsequently treated with doxycycline and/or transfected with two independent siRNAs targeting LGP2 (siLGP2 #1 or #2) or a control siRNA (siCtrl). (B) Cells were harvested 72 h post‐treatment and RT‐qPCR analysis was used to monitor knockdown efficiency of ADAR1 and LGP2 (*DHX58*) upon doxycycline treatment or siRNA transfection, respectively. All transcripts were normalized to ACTB. Data are means ± s.d. from a representative of three biological replicate experiments. (C) Cell confluency was measured every 4 h on a IncuCyte S3 Live‐Cell Analysis machine. Data are means ± s.e.m (16 replicate fields of view per experimental condition) from a representative of two biological replicate experiments. (D) Knockdown efficiency of ADAR1 and LGP2 (*DHX58*) in samples of (C) at 120 h post‐treatment was determined as in (B). Data are means ± s.d. from one experiment.

Recent studies have placed ADAR1 in the spotlight as an attractive novel drug target to enhance antitumor immunity (Bhate *et al*, 2019). To explore the relationship between LGP2 and ADAR1 and its prognostic value for overall patient survival, we performed *in silico* analysis of ADAR1 and LGP2 (encoded by *ADAR* and *DHX58*, respectively) mRNA expression in multiple cancer types using data from The Cancer Genome Atlas (TCGA). We hypothesized that patients with high *DHX58* expression (*DHX58^high^*) would have improved survival compared to patients with low *DHX58* expression (*DHX58^low^*) in patients with low *ADAR* expression (*ADAR^low^*). Patients were stratified into four groups according to *ADAR* and *DHX58* transcript levels using median cut‐offs. Consistent with the hypothesis, *ADAR^low^DHX58^high^* patients had improved overall survival compared to *ADAR^low^DHX58^low^* patients in bladder cancer (BLCA), breast cancer (BRCA), sarcoma (SARC), esophageal (ESCA) and liver (LIHC) cancer (Figs 5A and EV4A). In contrast, no difference in survival was found for these cancer types in *ADAR^high^* cancer patients stratified based on *DHX58* levels. Thus, LGP2 mRNA abundance is correlated with improved outcomes specifically in patients with low levels of ADAR1 across multiple malignancies.

**Figure EV5:**
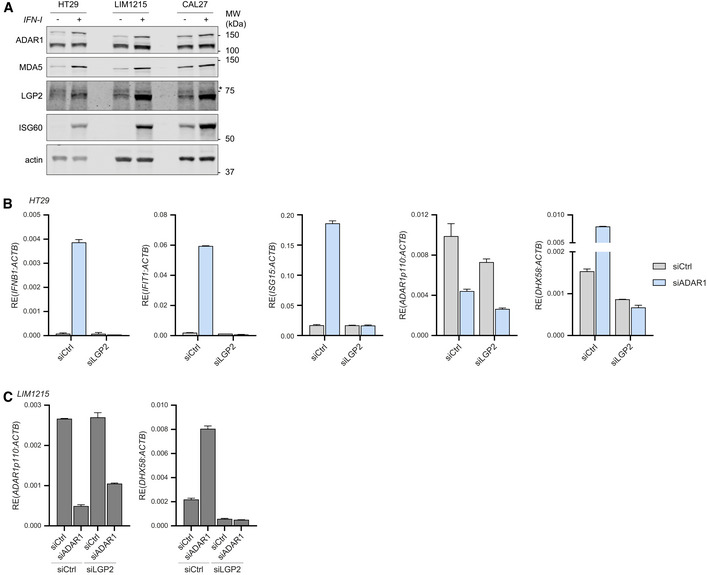
Type I IFN responsiveness and siADAR1‐dependent type I IFN induction in various cancer cell lines, related to Figs 5 and 6 Endogenous expression level and type I IFN inducibility of relevant proteins in HT29, LIM1215, and CAL27 cells. Cells were treated with or without recombinant type I IFN. Protein lysates were analyzed by SDS‐PAGE followed by immunoblotting with the indicated antibodies. *, nonspecific band.HT29 cells were transfected with the indicated siRNAs. Cells were harvested 72 h post‐transfection and RT‐qPCR analysis was used to monitor the type I IFN response (IFN‐β, IFIT1, and ISG15 transcripts) and knockdown efficiency of ADAR1 and LGP2 (*DHX58*). All transcripts were normalized to ACTB. Data are means ± s.d. from a representative of three biological replicate experiments.LIM1215 cells were transfected with the indicated siRNAs. Cells were harvested 72 h post‐transfection and knockdown efficiency of ADAR1 and LGP2 (*DHX58*) was analyzed as in (C). Data are means ± s.d. from a representative of three biological replicate experiments. Source data are available online for this figure.

Whether the above observations indicate a functional relationship between ADAR1 and LGP2 in tumors, or are merely a consequence of increased ISG expression in tumors with low ADAR1 expression, cannot be determined through bioinformatic analysis. To explore this experimentally, we used doxycycline‐inducible shRNA‐mediated knockdown of ADAR1 in a human oral squamous cell carcinoma cell line (CAL27), which was previously shown to be sensitive to ADAR1 loss (Liu *et al*, 2019) and that upregulates LGP2 upon type I IFN treatment (Fig EV5A). As predicted, knockdown of ADAR1 inhibited cell proliferation of CAL27 cells, whereas this was not the case upon expression of a control shRNA targeting GFP (Fig 5B and C). Importantly, simultaneous knockdown of LGP2 using two independent siRNAs resulted in a partial rescue of cell growth after ADAR1 knockdown (Fig 5B and C). ADAR1 and LGP2 knockdown efficiencies were validated by RT‐qPCR (Fig EV4B). The partial rescue of ADAR1‐mediated growth retardation is likely attributable to the incomplete knockdown of LGP2 as well as the activation of PKR upon ADAR1 loss, as reported (Chung *et al*, 2018; Liu *et al*, 2019). The above observations were confirmed using IncuCyte live‐cell analysis (Fig EV4C and D). We conclude that loss of LGP2 in part suppresses growth retardation following ADAR1 knockdown.

**Figure 6:**
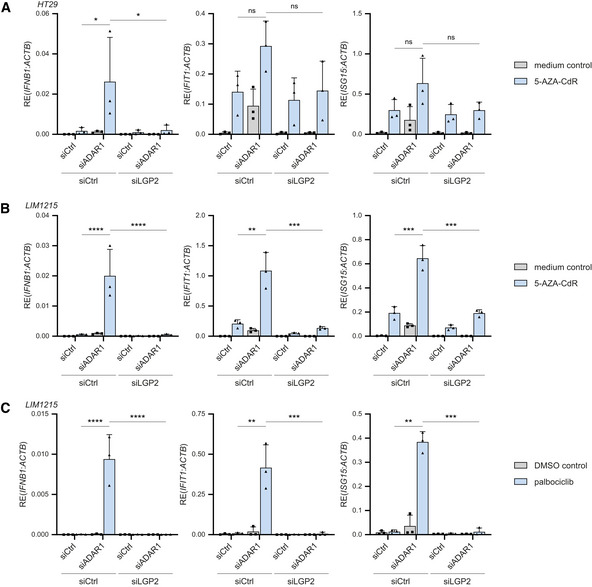
Depletion of ADAR1 in combination with epigenetic repressors trigger type I IFN induction in an LGP2‐dependent manner HT29 cells were treated with or without 300 nM 5‐AZA‐CdR for 2 days and subsequently washed and transfected with the indicated siRNAs. Cells were harvested 72 h post‐transfection and the type I IFN response was analyzed by RT‐qPCR analysis of IFN‐β, IFIT1, and ISG15 transcript expression, normalized to ACTB.LIM1215 cells were treated with or without 300 nM 5‐AZA‐CdR for 2 days and subsequently washed and transfected with the indicated siRNAs. Cells were harvested 72 h post‐transfection and the type I IFN response was analyzed as in (A).LIM1215 cells were treated with 250 nM of palbociclib or a DMSO control for 7 days. Three days after treatment initiation, cells were transfected with the indicated siRNAs and cultured for an additional 72 h. The type I IFN response was monitored as in (A). Data information: Data from three biological independent experiments are shown with mean ± s.d. Statistical analysis was performed using ordinary two‐way ANOVA with Tukey's *post hoc* test. ns, not significant; *P < 0.05; **P < 0.01; ***P < 0.001; ****P < 0.0001.

Treatment of cancer cells with DNA methyltransferase inhibitors (DNMTis) activates RNA sensors, including MDA5 and the endosomal dsRNA sensor Toll‐like receptor 3 (TLR3) (Chiappinelli *et al*, 2015; Roulois *et al*, 2015). Moreover, the combined treatment of patient‐derived colorectal cancer cell lines with the DNMTi 5‐aza‐2′‐deoxycytidine (5‐AZA‐CdR) together with shRNA‐mediated depletion of ADAR1 induces an MDA5‐dependent type I IFN response through the increased expression of IR‐Alu elements that are no longer edited (Mehdipour *et al*, 2020). To test whether this involves LGP2, we used siRNAs to deplete ADAR1, either alone or in combination with an siRNA targeting LGP2, in human colorectal adenocarcinoma cells (HT29). As expected, loss of ADAR1 triggered a type I IFN response in these cells, as determined by expression of IFN‐β and two ISGs (IFIT1 and ISG15), but this was completely blocked upon simultaneous depletion of LGP2 (Fig EV5B). The siADAR1‐mediated IFN response was further enhanced upon 5‐AZA‐CdR treatment of HT29 cells (Fig 6A), as previously reported (Mehdipour *et al*, 2020). Notably, the synergistic effect between ADAR1 depletion and 5‐AZA‐CdR treatment, which was most prominently detected at the level of IFN‐β rather than ISGs, was also strictly dependent on LGP2 (Fig 6A). In contrast, the elevated type I IFN response induced by treatment with 5‐AZA‐CdR alone was less dependent on LGP2, which indicates that some stimulatory RNAs that are demethylated and expressed upon 5‐AZA‐CdR treatment *per se* partially escape recognition by LGP2, likely through ADAR1‐mediated RNA editing. A possible explanation is that this LGP2‐independent ligand activates TLR3 (Chiappinelli *et al*, 2015). Knockdown efficiencies of ADAR1 and LGP2 were determined by RT‐qPCR analysis (Fig EV5B). The above observations were confirmed in another human colorectal carcinoma cell line (LIM1215) (Figs 6B and EV5C). To further explore the LGP2‐dependent synergy between ADAR1 depletion and anticancer therapies, we treated cells with ADAR1‐targeting siRNAs in the presence or absence of the CDK4/6 inhibitor palbociclib, which also upregulates levels of endogenous stimulatory RNAs (Goel *et al*, 2017). Combined treatment with ADAR1 siRNAs and palbociclib caused a synergistic upregulation of IFN‐β and ISGs, as described (Mehdipour *et al*, 2020), which was also strictly dependent on LGP2 (Fig 6C). We conclude that LGP2 is essential for the enhanced inflammatory response upon combined epigenetic therapy and ADAR1 depletion in tumor cells.

## Discussion

Nucleic acid sensors continuously survey their environment for the presence of nucleic acid structures that are commonly found on viral RNA. Various cellular mechanisms allow the discrimination between viral (nonself) nucleic acids and cellular (self) DNA or RNA. These mechanisms, however, are not foolproof. Loss of ADAR1‐dependent RNA editing causes unwanted recognition of self RNA and consequently inadvertent innate immune activation and severe pathology (Rice *et al*, 2012, 2017; Livingston & Crow, 2016; Rodero & Crow, 2016). Various nucleic acid sensors, including PKR, OAS/RNase L, and MDA5, sense unedited self RNA and cause translational shutdown, cell death, and autoinflammation, marked by the production of type I IFNs (Quin *et al*, 2021). Here, we demonstrate that the RNA helicase LGP2 is indispensable for type I IFN induction in ADAR1‐deficient human cells, including primary human monocyte‐derived macrophages. We further demonstrate that this involves the canonical role of LGP2 as a sensor of base‐paired RNA, which requires ATP hydrolysis and intact RNA‐binding sites and enables MDA5 oligomerization. We extend our findings to multiple cancer cell lines (THP‐1, CAL27, HT29 and LIM1215) and demonstrate that the sensitivity of tumor cells to ADAR1 loss requires the presence of LGP2. Moreover, the previously reported synergistic effects of ADAR1 depletion and epigenetic therapy on the intrinsic type I IFN response in cancer cells are also strictly dependent on LGP2. These findings have several important implications, which are discussed below.

The key role of LGP2 in the sensing of unedited self RNA sheds new light on the search for the, largely elusive, stimulatory RNAs in ADAR1 deficiency. Multiple studies have defined the ADAR1 “editome” (Ramaswami & Li, 2016). The vast majority of A‐to‐I editing sites is found in or near Alu elements, and a small proportion is found in other mobile repeat elements, such as long interspersed nuclear elements (LINEs) and endogenous retroviruses (ERVs) (Ramaswami *et al*, 2012). Alu elements are mostly embedded within 3′UTRs or introns of Pol II transcripts, while few are transcribed as individual units by Pol III (Deininger, 2011; Chung *et al*, 2018). The repetitive nature of mobile elements, especially when found in close proximity to each other and in inverted orientation, increases the risk of forming endogenous base‐paired structures that may activate innate immune pathways (Eisenberg & Levanon, 2018). Editing of repeat elements by ADAR1 may decrease the high degree of complementarity in such RNAs and minimize accidental innate immune activation. An elegant study indeed demonstrated that, *in vitro,* recombinant MDA5 preferentially binds unedited IR‐Alu elements among total cytosolic RNA extracted from ADAR1‐deficient cells (Ahmad *et al*, 2018). However, editing levels are often low within base‐paired stem regions of Alu and IR‐Alu elements while being more frequent within predicted single stranded regions (Chung *et al*, 2018). In addition, endogenous base‐paired RNAs with near‐perfect complementarity are scarce among mRNAs (but not pre‐mRNAs) (Barak *et al*, 2020). In mice, transcriptomic analysis of ADAR p110/ADAR2‐deficient brain tissue revealed as few as 36 ADAR1 p150‐specific editing sites (Kim *et al*, 2021). Thus, potentially, only few RNAs become truly stimulatory when no longer edited in intact cells or tissues. The stimulatory potential of endogenous RNA also depends on its conformation. RNA that adopts a noncanonical Z‐conformation has increased immunostimulatory potential. The latter is reduced upon binding and editing by ADAR1 p150, which contains a Z‐nucleic acid (Zα) binding domain (de Reuver *et al*, 2021; Maurano *et al*, 2021; Nakahama *et al*, 2021; Tang *et al*, 2021; Zillinger & Bartok, 2021). In mice, various mutations in the Zα domain of ADAR1 result in postnatal growth retardation and mortality as well as an increased type I IFN signature (de Reuver *et al*, 2021; Maurano *et al*, 2021; Nakahama *et al*, 2021; Tang *et al*, 2021). Transcripts that are prone to adopt a Z‐conformation, such as those with purine‐pyrimidine repeats (Koeris *et al*, 2005), can therefore be immunostimulatory, and this can be reduced by A‐to‐I editing. The precise identity and features of immunogenic RNAs remain unresolved. Protein–RNA interaction studies, such as individual‐nucleotide resolution UV‐crosslinking and immunoprecipitation (iCLIP), may help to identify RNAs that directly engage dsRNA sensors. The role of LGP2 in unedited self RNA sensing opens up the possibility to retrieve immunostimulatory RNAs via their association with LGP2 in ADAR1‐deficient cells, as has been done in the context of viral infection (Deddouche *et al*, 2014). Our observation that type I IFN induction upon loss of ADAR1 is strictly dependent on LGP2, which favors the formation of short RNA filaments (Bruns *et al*, 2014), hints at the possibility that the stimulatory RNA in ADAR1 deficiency is shorter than “classical” MDA5 substrates, which tend to be long complex dsRNAs of > 1,000/2,000 nt in length (Pichlmair *et al*, 2009; Kato *et al*, 2021).

Besides ADAR1, eight disease‐causative mutations have been identified in AGS, including gain‐of‐function mutations in *IFIH1* (encoding MDA5), all of which cause an elevated type I IFN signature (Livingston & Crow, 2016; Rodero & Crow, 2016; Uggenti *et al*, 2019, 2020). Currently, there is no licensed therapy for treatment of AGS or related interferonopathies (Crow *et al*, 2020). A few individual reports describe encouraging clinical improvements upon treatment of a small number of patients with the JAK1/2 inhibitor ruxolitinib, which blocks signaling downstream of the IFNAR (Crow *et al*, 2020). However, JAK1/2 inhibition is rather nonspecific and will block pathways beyond type I IFN signaling. In addition, transcriptional activity of IRF3 and NF‐κB leads to upregulation of other genes and cytokines, besides type I IFNs, which perhaps contribute to pathology as well (Andersen *et al*, 2008; Rehwinkel & Gack, 2020). Compounds that target the upstream nucleic acid sensing machinery, such as LGP2, may therefore have therapeutic value. In contrast to ruxolitinib, inhibition of LGP2 will prevent IRF3 and NF‐κB activation while minimally impacting on viral nucleic acid sensing via RIG‐I and DNA sensors, leaving patients less prone to a wide spectrum of viral infections.

Our findings on the essential role of LGP2 in type I IFN induction caused by ADAR1 dysfunction are strengthened by a recent report in which *Adar^P195A^* ^/^ *^p150^* ^−^ mice, which bear a mutation in the Zα domain of ADAR1 p150 (P195A) paired with a null allele of *Adar* (mimicking the most common *ADAR* mutation in AGS at P193), were intercrossed with various knockout models, including *Dhx58* ^−/−^ mice (Maurano *et al*, 2021). LGP2 deficiency rescued the postnatal mortality of *Adar^P195A^* ^/^ *^p150^* ^−^ mice and abolished the type I IFN signature. Thus, LGP2 is an essential effector molecule of ADAR1‐driven disease in both mice and humans. Loss of PKR also rescued the mortality of *ADAR^P195A^* ^/^ *^p150^* ^−^ mice, yet the type I IFN signature remained elevated (Maurano *et al*, 2021), consistent with previous literature showing that activation of PKR is largely responsible for ADAR1‐associated translational shutdown, cell death, and pathology but not IFN‐driven inflammation (Chung *et al*, 2018).

In contrast to its disease‐causing role in AGS, ADAR1 is an exciting new immuno‐oncology target and several *in vitro* and *in vivo* studies have highlighted that its deletion increases tumor cell lethality and renders tumors more vulnerable to immunotherapy (Gannon *et al*, 2018; Ishizuka *et al*, 2019; Liu *et al*, 2019). We observed that depletion of ADAR1 in multiple tumor cell lines triggered a type I IFN response in an LGP2‐dependent manner. Moreover, the reported synergy between ADAR1 deletion and epigenetic therapy was completely dependent on the expression of LGP2. Finally, LGP2 was required for reduced cell growth upon ADAR1 knockdown. Altogether, this demonstrates that LGP2 is a hitherto overlooked, yet essential, player when targeting ADAR1. It also predicts that LGP2‐sufficient tumors are more likely to respond to ADAR1‐directed therapies than LGP2‐deficient tumors. Indeed, across multiple human tumor types, patient stratification based on ADAR1 and LGP2 transcript levels revealed that patients with high LGP2 and concomitant low ADAR1 levels had improved survival. The relationship between ADAR1 and LGP2 and its impact on tumor growth, the intra‐tumoral inflammatory response, and antitumor immunity will need to be further evaluated in *in vivo* models.

Collectively, our data identify LGP2 as an important sensor of endogenous stimulatory RNA and as an essential player in autoinflammation driven by ADAR1 dysfunction with important implications for treatment of type I interferonopathies as well as for potential ADAR1‐directed cancer therapy.

## Materials and Methods

### Cell culture and reagents

HEK293, HEK293T, and CAL27 (ATCC) were cultured in Dulbecco's modified Eagle's medium (DMEM; Gibco, Thermo Fisher Scientific) supplemented with 10% heat‐inactivated fetal calf serum (FCS, Sigma), 2 mM of glutamine and 100 U/ml of penicillin/streptomycin (Gibco, Thermo Fisher Scientific). LIM1215 cells (gift from René Bernards, NKI) were cultured in McCoy's 5A medium (Gibco, Thermo Fisher Scientific) supplemented with 10% FCS, 2 mM of glutamine and 100 U/ml of penicillin/streptomycin. THP‐1 Dual (Invivogen) and HT29 (gift from Jacques van Dongen, LUMC) were cultured in Roswell Park Memorial Institute (RPMI) 1640 medium containing 10% FCS, 2 mM of glutamine, and 100 U/ml of penicillin/streptomycin. THP‐1 monocytes were differentiated into macrophage‐like cells by treatment with 150 nM of PMA (Sigma) for 24 h, after which PMA was washed away and cells were cultured for an additional 24 h before use in experiments. Human materials were obtained in accordance with the Declaration of Helsinki and the Dutch rules with respect to the use of human materials from volunteer donors. Buffy coats from healthy anonymized donors were obtained after their written informed consent, as approved by Sanquin's internal ethical board. Human PBMCs were isolated from buffy coats using Leucosep tubes (Greiner Bio‐One), according to the manufacturer's instructions. Monocytes were purified from PBMCs using positive selection with UltraPure CD14 MicroBeads (Miltenyi Biotec) and MACS LS separation columns (Miltenyi Biotec), according to the manufacturer's instructions. For differentiation of macrophages, monocytes were cultured in T75 flasks (Corning) in RPMI 1640 supplemented with 10% FCS, 2 mM of glutamine, 1 mM of sodium pyruvate (Gibco, Thermo Fisher Scientific), and 50 ng/ml of M‐CSF (premium grade, Miltenyi Biotec). Medium was refreshed every 3–4 days. After 6 days, macrophages were replated at 250,000 cells per well in 24‐well plates for experiments. Cells were grown at 37°C (HEK293 at 10% CO_2_; all others at 5% CO_2_). Universal Type I IFN α (11200, PBL Assay Science) was used at 500–1,000 U/ml. Doxycycline hexahydrate (Sigma) was used at 0.5–1 µg/ml. 5‐aza‐2′‐deoxycytidine (5‐AZA‐CdR; A3656, Sigma) was dissolved in PBS and used at 300 nM. Palbociclib isethionate (HY‐A0065, MedChemExpress) was dissolved in DMSO and used at 250 nM. High molecular weight (HMW) poly(I:C) (Invivogen) was complexed with Lipofectamine 2000 (Thermo Fisher Scientific) at a 1:1 ratio and used at the indicated dosages. RNA extracted from EMCV‐infected HeLa cells was prepared as described before (van der Veen *et al*, 2018). RNA was complexed and transfected as described for poly(I:C). Ribavirin (R9644, Sigma) was used at 200 µM. For antibiotic selection, puromycin (Sigma), geneticin (G418; Invivogen), and hygromycin B (Roche) were used at concentrations mentioned below.

### Plasmids, siRNA, and transfection

Generation of pcDNA3.1 plasmids encoding 3FLAG‐human LGP2, 3FLAG LGP2 N‐terminal domain (NTD; 1–552), 3FLAG‐LGP2 K634E, and 3FLAG‐human MDA5 has been described before (Pichlmair *et al*, 2009; van der Veen *et al*, 2018). The 3FLAG C‐terminal domain of LGP2 (CTD; 545–678) was generated by PCR amplification and was cloned into pcDNA3.1. The MDA5 2CARD deletion mutant (ΔCARD; 295–1,025) was a kind gift of Dr. Jan Rehwinkel. The 3FLAG‐LGP2 K138E/R490E, 3FLAG‐LGP2 K138E/R490E/K634E (KRK mutant), 3FLAG‐LGP2 K30A, 3FLAG‐LGP2 C615A, 3FLAG‐LGP2 Q390R/T395R, 3FLAG‐LGP2 Q390A/Q394A, 3FLAG‐MDA5 G495R, 3FLAG‐MDA5 R728A, 3FLAG‐MDA5‐H927A, 3FLAG‐MDA5 R728/H927A, 3FLAG‐MDA5 M570R/D572R, and 3FLAG‐MDA5 I841R/E842R mutations were introduced using the QuikChange II Site‐Directed Mutagenesis Kit (Agilent). pmGFP‐ADAR1‐p110 and pmGFP‐ADAR1‐p150 were a gift from Kumiko Ui‐Tei (Addgene, #117928 and #117927). For gene editing in HEK293, single guide (sg)RNAs against *ADAR* (ADAR1), *DDX58* (RIG‐I), *IFIH1* (MDA5) or *DHX58* (LGP2) were designed using the Horizon, IDT and Zhang lab CRISPR design tools and cloned into pSpCas9(BB)‐2A‐Puro (pX459), which was a gift from Feng Zhang (Addgene plasmid #62988), by introducing hybridized oligos (Table 1) via BbsI restriction sites. All plasmids were transfected using Lipofectamine 2000 (Invitrogen, Thermo Fisher Scientific) according to the manufacturer's instructions.

**Table 1:** Oligonucleotide sequences.

Target	Sequence (5′–3′)
Primer sequences used for RT‐qPCR	
*IFNB1*	FW: AGTAGGCGACACTGTTCGTG
	RV: GTCTCATTCCAGCCAGTGCT
*IFIT1*	FW: CAGAATAGCCAGATCTCAGAGG
	RV: CCAGACTATCCTTGACCTGATG
*ISG15*	FW: CTCATCTTTGCCAGTACAGGAG
	RV: CCAGCATCTTCACCGTCAG
*ADAR1p110* (p110 isoform‐specific)	FW: GGCAGTCTCCGGGTG
	RV: CTGTCTGTGCTCATAGCCTTGA
*IFIH1*	FW: GGAGTCAAAGCCCACCATCT
	RV: TGTTCATTCTGTGTCATGGGTTT
*DHX58*	FW: CTGCTCATCCATGACACCGT
	RV: GCTCATTCTTGCGGTCATCG
*ACTB*	FW: CACTCTTCCAGCCTTCCTTC
	RV: TACAGGTCTTTGCGGATGTC
sgRNA primer sequences used for CRISPR‐Cas9 target gene editing (underlined are cloning overhangs)	
*DDX58* #1 (for cloning in pX459)	FW: CACCGTGCAGGCTGCGTCGCTGCT
	RV: AAACAGCAGCGACGCAGCCTGCAC
*DDX58* #2 (for cloning in pX459)	FW: CACCGGATTATATCCGGAAGACCC
	RV: AAACGGGTCTTCCGGATATAATCC
*IFIH1* #3 (for cloning in pX459)	FW: CACCGTAGCGGAAATTCTCGTCTG
	RV: AAACCAGACGAGAATTTCCGCTAC
*IFIH1* #4 (for cloning in pX459)	FW: CACCGGGTTGGACTCGGGAATTCG
	RV: AAACCGAATTCCCGAGTCCAACCC
*DHX58* #5 (for cloning in pX459)	FW: CACCGGAGCTTCGGTCCTACCAAT
	RV: AAACATTGGTAGGACCGAAGCTCC
*DHX58* #6 (for cloning in pX459)	FW: CACCGGGGTCTTCCCGGCACCCGT
	RV: AAACACGGGTGCCGGGAAGACCCC
*ADAR* #1 (for cloning in pX459)	FW: CACCGAAATGCTGTGCTAATTGACA
	RV: AAACTGTCAATTAGCACAGCATTTC
*ADAR* #2 (for cloning in pX459)	FW: CACCGATGATGGCTCGAAACTCACC
	RV: AAACGGTGAGTTTCGAGCCATCATC
*DDX58* #1 (for cloning in AA19_pLKO)	FW: ACCGTTGCAGGCTGCGTCGCTGCT
	RV: AAACAGCAGCGACGCAGCCTGCAA
*DDX58* #2 (for cloning in AA19_pLKO)	FW: ACCGGATTATATCCGGAAGACCC
	RV: AAACGGGTCTTCCGGATATAATC
*IFIH1* #3 (for cloning in AA19_pLKO)	FW: ACCGATAGCGGAAATTCTCGTCTG
	RV: AAACCAGACGAGAATTTCCGCTAT
*IFIH1* #4 (for cloning in AA19_pLKO)	FW: ACCGTGGTTGGACTCGGGAATTCG
	RV: AAACCGAATTCCCGAGTCCAACCA
*DHX58* #5 (for cloning in AA19_pLKO)	FW: ACCGGAGCTTCGGTCCTACCAAT
	RV: AAACATTGGTAGGACCGAAGCTC
*DHX58* #6 (for cloning in AA19_pLKO)	FW: ACCGCGGGTCTTCCCGGCACCCGT
	RV: AAACACGGGTGCCGGGAAGACCCG
shRNA primer sequences used for target gene silencing (underlined are cloning overhangs and loop)	
*ADAR* (for cloning in Tet‐pLKO‐neo)	FW: CCGGGCCCACTGTTATCTTCACTTTCTCG AGAAAGTGAAGATAACAGTGGGCTTTTTG
	RV: AATTCAAAAAGCCCACTGTTATCTTCACT TTCTCGAGAAAGTGAAGATAACAGTGGGC
*GFP* (for cloning in Tet‐pLKO‐neo)	FW: CCGGGCAAGCTGACCCTGAAGTTCATCTCGAG ATGAACTTCAGGGTCAGCTTGCTTTTTG
	RV: AATTCAAAAAGCAAGCTGACCCTGAAGT TCATCTCGAGATGAACTTCAGGGTCAGCTTGC

For gene silencing in cell lines, individual siGenome silencing (si)RNAs against human ADAR1 (D‐008630‐04), human LGP2 (D‐010582‐01, D‐010582‐02, D010582‐04), and a scrambled control (D‐001210‐02 or D‐001210‐03) were purchased at Dharmacon. HEK293, HT29, and LIM1215 were transfected with 25 pmol/ml of each siRNA; CAL‐27 was transfected with 5 pmol/ml siRNA using DharmaFECT 1 (Dharmacon) according to the manufacturer's instructions. THP‐1 was transfected with 50 pmol/ml siRNA using TransIT‐TKO (Mirus Bio) according to the manufacturer's instructions.

For gene silencing in primary cells, SMARTpool ON‐TARGETplus siRNAs against human ADAR1 (L‐008630‐00‐0005), human LGP2 (L‐010582‐00‐0005), and a nontargeting control pool (D‐001810‐10‐05) were purchased at Dharmacon. Primary macrophages were replated 1 day prior to transfection and were transfected with 37.5 pmol of each siRNA indicated using HiPerFect (Qiagen), as described (Troegeler *et al*, 2014).

For viral delivery, wild‐type human 3FLAG‐LGP2 or the KRK mutant was cloned via PCR amplification into the lentiviral plasmid pLVX‐Tight‐Puro (Clontech), to allow doxycycline‐inducible expression when used together with the Lenti‐X Tet‐On Advanced lentiviral expression system (Clontech), or into the retroviral plasmid pMSCV‐puro (Clontech). shRNAs directed against ADAR1 or GFP were cloned into Tet‐pLKO‐neo, a gift from Dmitri Wiederschain (Addgene, #21916). All shRNA sequences are listed in Table 1. For gene editing in THP‐1, sgRNAs against *DDX58* (RIG‐I), *IFIH1* (MDA5), or *DHX58* (LGP2) were cloned into a pLKO.1‐puro‐derived vector (AA19_pLKO, gift from Manuel Gonçalves (Chen *et al*, 2016)) by introducing hybridized oligos (Table 1) via BveI restriction sites. Retro‐ and lentiviral particles were prepared as described below.

### Retro‐ and lentivirus production and transduction

Retroviral and lentiviral particles were produced by transfecting HEK293T cells with transfer vectors. The above‐mentioned retroviral and lentiviral transfer vectors were combined with psPAX2 (Addgene, 12260; gift from Didier Trono) and pMD2.G (Addgene, 12259; gift from Didier Trono) at a 4:3:1 ratio and transfected using polyethylenimine (PEI; 23966, Polysciences). Medium was changed 16 h post‐transfection and virus‐containing cell culture supernatants were harvested after 48 and 72 h and passed through a 0.45‐µm filter or centrifuged for 5 min at 3,000 *g* at 4°C to remove cellular debris. Viral supernatants were used unconcentrated, or concentrated 100× by high‐speed centrifugation (10,000 *g*, 4 h, 4°C) using a 10% sucrose‐containing buffer (50 mM of Tris–HCl pH 7.5, 100 mM of NaCl, 0.5 mM of EDTA, 10% sucrose) as described (Jiang *et al*, 2015).

Stable LGP2‐expressing HEK293 were generated through retroviral transduction followed by puromycin selection (1 µg/ml). The doxycycline‐inducible expression of 3FLAG‐LGP2 or 3FLAG‐LGP2 KRK mutant was introduced in ADAR1 KO HEK293 cells (clone 1) through sequential transduction of the Tet‐On advanced system and selection with 500 µg/ml G418 followed by transduction of pLVX‐Tight‐Puro‐based lentiviruses and selection with 1 µg/ml of puromycin. Inducible expression of LGP2 was verified by treating cells with 1 µg/ml doxycycline for 72 h and analysis by immunoblotting and RT‐qPCR. To introduce (doxycycline‐inducible) expression of shRNAs in CAL27, cells were transduced with Tet‐pLKO‐neo‐shADAR1 or ‐shGFP lentiviruses and selected with G418 (400 µg/ml). Silencing of ADAR1 was confirmed by immunoblotting and RT‐qPCR.

### Generation of gene knockouts using CRISPR‐Cas9

To generate ADAR1, RIG‐I, MDA5, or LGP2 knockout HEK293, cells were seeded in a 6‐well plate and transfected with 2 µg of the respective pX459‐sgRNA plasmid(s) (described above). After 24 h, culture medium was replaced with medium containing 1 µg/ml of puromycin for 36 h. Single cells were subsequently seeded in 96‐well plates and expanded for screening by immunoblotting.

To generate RIG‐I, MDA5, or LGP2 knockout THP‐1 monocytes, cells were seeded in 6‐well plates and transduced with lentivirus supernatant for delivery of AA19_pLKO‐sgRNAs by means of spin infection (90 min, 2,000 *g*, 33°C) in the presence of 4 μg/ml of polybrene (#107689, Merck). After 72 h, cells were selected with medium containing 0.45 μg/ml of puromycin for 72 h. Transient expression of Cas9 introduced in sgRNA‐expressing THP‐1 cells by infection with the adenoviral vector AdV.PGK.Cas9. SV40pA.dE2A.F50 (1.39 × 10^11^ TCID_50_/ml; kind gift of Manuel Gonçalves (Maggio *et al*, 2014)). Medium was refreshed at 24 h post‐infection. At 14 days post‐infection, cells were single cell‐sorted into 96‐well plates using an Aria III (BD Biosciences). After clonal expansion, single cell clones were screened for correct gene editing by immunoblotting.

### Immunoblotting

Cells were washed once in PBS and lysed in plates using SDS lysis buffer (1% SDS, 150 mM of NaCl, 50 mM of Tris pH 7.5, protease inhibitors (Complete EDTA‐free, Roche), and benzonase (Santa Cruz)). For detection of phosphorylated proteins, cells were lysed on ice in RIPA lysis buffer (Pierce), supplemented with protease inhibitors, benzonase, and phosphatase inhibitor cocktail set V (Calbiochem). Lysates for phospho‐blotting were cleared by centrifugation (15 min, 13,500 *g* at 4°C). Protein concentrations were assessed by BCA Protein Assay (Pierce, Thermo Fisher Scientific) and subsequently equalized. Samples were resolved along with Precision Plus Protein Dual Color Standards (Bio‐Rad) on 4–15% or 10% Mini‐PROTEAN TGX precast gels (Bio‐Rad) or 4–20% Novex Tris‐Glycine gels (Invitrogen) and transferred onto PVDF or nitrocellulose membranes (both from Bio‐Rad) by semi‐dry transfer. Membranes were blocked in 5% nonfat dried milk (NFDM) in PBS‐T (PBS, 0.1% Tween‐20) or, for phospho‐blotting, in sterile‐filtered 5% BSA (Millipore) in TBS‐T (20 mM of Tris pH 7.5, 150 mM of NaCl, 0.1% Tween‐20). Membranes were incubated with the relevant primary and secondary antibodies in either 5% NFDM in PBS‐T or Western BLoT Immuno Booster 1 or 2 solution (Takara Bio). Antibodies and dilutions are listed in Table 2. PVDF membranes were developed on a ChemiDoc XP machine (Bio‐Rad) using Luminata Crescendo or Luminata Forte ECL substrate (both Millipore). Nitrocellulose membranes were developed on an Odyssey CLX‐1391 (LI‐COR Biosciences).

**Table 2:** Antibodies used for immunoblotting.

Target/Epitope	Dilution	Catalog no. and company
ADAR1	1:500	sc‐73408 (Santa Cruz)
ISG60	1:500	sc‐393512 (Santa Cruz)
β‐actin	1:10,000	A5441 (Sigma)
FLAG	1:20,000	F1804 (Sigma)
RIG‐I	1:1,000	#3743 (Cell Signaling Technology)
MDA5	1:1,000	#5321 (Cell Signaling Technology)
MDA5	1:1,000	21775‐1‐AP (ProteinTech)
LGP2	1:400	29030 (IBL)
p‐IRF3 (Ser396)	1:1,000	#4947 (Cell Signaling Technology)
IRF3	1:1,000	#11904 (Cell Signaling Technology)
p‐STAT1 (Tyr701)	1:1,000	#7649 (Cell Signaling Technology)
STAT1	1:1,000	#9172 (Cell Signaling Technology)
β‐actin‐HRP	1:10,000	sc‐47778 (Santa Cruz)
FLAG‐HRP	1:20,000	A8592 (Sigma)
Goat‐anti‐mouse IgG‐HRP	1:10,000	G‐21040 (Invitrogen)
Goat‐anti‐rabbit IgG‐HRP	1:20,000	4050‐05 (Southern Biotech)
Goat‐anti‐mouse IgG IRDye 680LT	1:5,000	926‐68020 (LI‐COR Biosciences)
Goat‐anti‐rabbit IgG IRDye 800CW	1:5,000	926‐32211 (LI‐COR Biosciences)

### Semi‐denaturing detergent agarose gel electrophoresis (SDD‐AGE)

To assess MDA5 or LGP2 oligomerization, cells were stimulated as described in the figure legends, harvested by trypsinization, and lysed in SDD‐AGE lysis buffer (0.5% IGEPAL CA‐630 (Sigma), 50 mM of Tris pH 7.4, 150 mM of NaCl, 10% glycerol) supplemented with protease inhibitors (cOmplete EDTA‐free, Roche) for 30 min while rotating at 4°C. Lysates were cleared by centrifugation (15 min, 13,500 *g* at 4°C), and protein concentrations were measured by BCA Protein Assay. Equal amounts of total proteins for each sample were incubated with 4× SDD‐AGE sample buffer (2× TBE, 20% glycerol, 8% SDS, 0.01% bromophenol blue) at room temperature for 15 min. Samples were resolved on vertical 1.5% agarose gels containing 1× TBE in 0.1% SDS in running buffer (1× TBE and 0.1% SDS). Proteins were transferred onto Immun‐Blot PVDF membranes (Bio‐Rad) and analyzed by immunoblotting as described above.

### RT‐qPCR

Total RNA was extracted using TRIzol or TRIzol LS (Invitrogen) according to the manufacturer's instructions. Five hundred nanograms of RNA were subsequently treated with ezDNase and reverse‐transcribed using SuperScript IV VILO Master Mix (both Invitrogen) according to the manufacturer's instructions. cDNA was diluted in nuclease‐free water and gene expression was measured in technical duplicates or triplicates using PowerUp SYBR Green Master Mix (Applied Biosystems) on a QuantStudio 3 (Thermo Fisher Scientific). Gene‐specific primers are listed in Table 1.

### Cell growth assays

CAL27 cells transduced with inducible shADAR1 (or shGFP) were seeded at 30,000 cells per well in a 12‐well plate. The following day, doxycycline was added at a final concentration of 1 µg/ml and cells were transfected with siLGP2 or siCtrl as described above. Doxycycline‐containing medium was refreshed 72 h post‐transfection. Cell confluency was measured every 4 h on a IncuCyte S3 Live‐Cell Analysis machine (Essen Bioscience) or at an end point of 120 h post‐siRNA transfection by Crystal Violet staining. For staining, cells were fixed in 100% ice‐cold methanol for 10 min, washed with PBS, and stained for 10 min with 0.1% Crystal Violet (V5265, Sigma) in 40% methanol. Stained plates were washed with water and dried, before imaging on a GelCount machine (Oxford Optronics Ltd.). Upon imaging, Crystal Violet was extracted from the stained cells by incubation in 15% acetic acid at RT for 20 min. The amount of extracted Crystal Violet was quantified by measuring the optical density at 590 nm using an Infinite M Plex plate reader (Tecan).

### Confocal microscopy

Cells were seeded and cultured in 8‐well Millicell EZ slides (Millipore). Where indicated, cells were transfected with siRNAs prior to seeding or treated with 1 μg/ml doxycycline after seeding. Cells were fixed with 4% PFA and permeabilized using 0.25% Triton X‐100 (Sigma) in PBS. Cells were stained with antibodies diluted in TNB (TBS, 0.5% blocking reagent (#11096176001, Roche), 0.02% Thimerosal (Sigma)). Washing steps were performed using TBS containing 0.05% Tween‐20. Cells were stained with the primary antibodies mouse α‐FLAG (clone M2, Sigma) and rabbit α‐IRF3 (#11904, Cell Signaling Technology) and secondary antibodies goat α‐mouse‐IgG1‐Alexa647 (A‐21240, Invitrogen) goat α‐rabbit‐Alexa488 (A‐11008, Invitrogen). Samples were embedded in DAPI‐containing Prolong Gold antifade mountant (Invitrogen). Images were acquired on a Leica TCS SP5 confocal microscope using a 63×/1.40 oil objective. Image analysis was performed using CellProfiler 4.1.3 software to quantify the occurrence of IRF3 nuclear translocation in a semi‐automated fashion. Of each experimental condition, multiple images (each with 30–100 cells in view) were analyzed, in order to have at least 450 nuclei included in the analyses.

### Statistical analysis

Statistical analysis was performed using GraphPad Prism software. Data distribution was first assessed for normality using D'Agostino‐Pearson and Shapiro‐Wilk normality tests. Nonparametric data were analyzed using the unpaired two‐tailed Mann–Whitney *U* test for single comparisons or the Kruskal–Wallis test for multiple comparisons, followed by Dunn's *post hoc* test. Multiple‐group analysis was carried out by ordinary two‐way ANOVA, followed by Tukey's *post hoc* test. Nonparametric data were log‐transformed prior to two‐way ANOVA analysis.

### Bioinformatic analysis of human tissues and cancer patient data

RNA sequencing data from TCGA studies were downloaded from the Broad Institute portal (https://gdac.broadinstitute.org). We focused on solid tumor types from organs not including the brain and with more than 25 survival events (*n* = 17). Level 3, log_2_‐RSEM normalized data files were utilized for downstream analysis. Normal tissue samples were discarded from subsequent analysis. Pan‐cancer clinical data including overall survival information were downloaded from Synapse (https://www.synapse.org) under accession syn12026747. All bioinformatic analysis of the TCGA data was carried out in R (version 4.0.3) using Rstudio. The survival (version 3.2‐7) package was used to fit Cox regression models to the data. Patients in each dataset were stratified according to median cut‐off values for mRNA expression levels of the genes encoding ADAR1 and LGP2, *ADAR* and *DHX58,* respectively. To predict overall survival, multivariate Cox regression models were fitted using ADAR and DHX58 stratifiers as covariates. To compare individual groups, univariate Cox regression models were fitted separately in ADAR1 high and low patients using DHX58 status as predictor. From these univariate models, hazard ratios, 95% confidence intervals, and Wald test *P* values were extracted and plotted using ggplot2 (version 3.3.5) and survminer (version 0.4.9).

## Author contributions

**Jorn E Stok:** Data curation; Formal analysis; Investigation; Visualization; Methodology; Writing—review and editing. **Timo Oosenbrug:** Data curation; Formal analysis; Investigation; Visualization; Methodology; Writing—review and editing. **Laurens R ter Haar:** Investigation; Writing—review and editing. **Dennis Gravekamp:** Investigation; Writing—review and editing. **Christian P Bromley:** Data curation; Formal analysis; Investigation; Visualization; Methodology; Writing—review and editing. **Santiago Zelenay:** Data curation; Formal analysis; Writing—review and editing. **Caetano Reis e Sousa:** Funding acquisition; Writing—review and editing. **Annemarthe G van der Veen:** Conceptualization; Data curation; Formal analysis; Supervision; Funding acquisition; Investigation; Visualization; Methodology; Writing—original draft; Project administration.

In addition to the CRediT author contributions listed above, the contributions in detail are:

JES, TO, and AGV designed experiments and analyzed data. JES, TO, and AGV conducted experiments with assistance from LRH and DG. CPB and SZ performed the TCGA bioinformatic analysis. CRS provided advice and sponsored the initial stages of the project. AGV supervised the project. AGV wrote the manuscript with assistance from JES and TO. All authors reviewed the manuscript.

## Supporting information



Expanded View Figures PDF

Source Data for Expanded View

Source Data for Figure 1

Source Data for Figure 2

Source Data for Figure 3

Source Data for Figure 4

## Data Availability

This study includes no data deposited in external repositories.
