# Supplementary material for: RNA sensing via the RIG‐I‐like receptor LGP2 is essential for the induction of a type I IFN response in ADAR1 deficiency
Source: EMBO J. 2022 Feb 14;41(6):e109760. doi: 10.15252/embj.2021109760 (PMC8922249; doi:10.15252/embj.2021109760)
Supplement: Supplementary file 1 — Expanded View Figures PDF [file EMBJ-41-e109760-s002.pdf]

Expanded View Figures

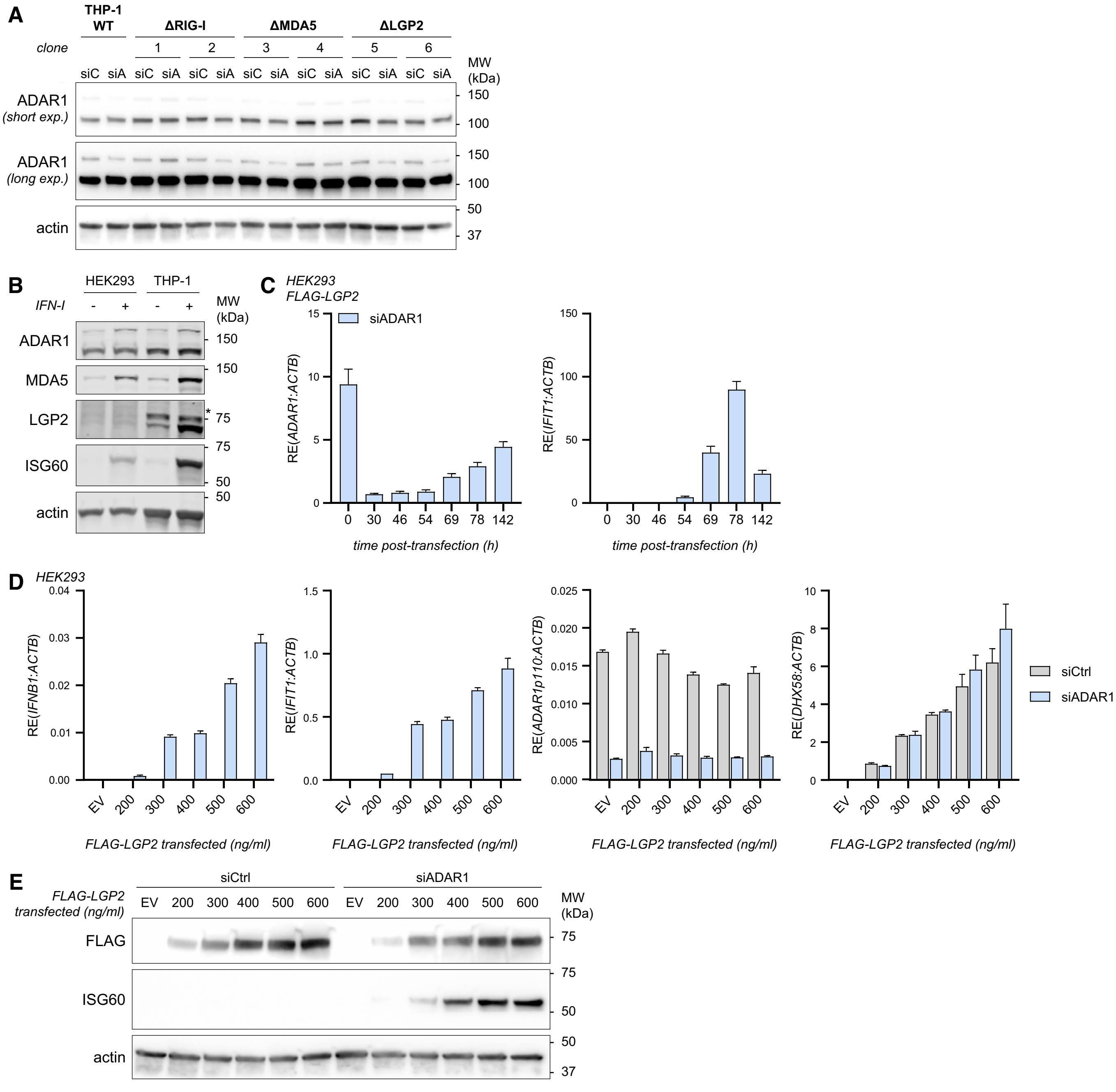

Figure EV1.

Figure EV1. LGP2 is required for the induction of a type I IFN response upon siRNA-mediated depletion of ADAR1 in HEK293, related to Fig 1.

A PMA-differentiated THP-1 WT, RIG-I-, MDA5-, and LGP2-knockout cells were transfected with siCtrl (siC) or siADAR1 (siA). Protein lysates were prepared 56 h post-transfection and ADAR1 knockdown efficiency was monitored by immunoblot analysis. Data correspond to the biological replicate shown in Fig 1B.

B Expression level and type I IFN inducibility of relevant proteins in HEK293 and THP-1. HEK293 and PMA-differentiated THP-1 cells were treated with or without recombinant type I IFN. Protein lysates were analyzed by SDS-PAGE followed by immunoblotting with the indicated antibodies ($n = 3$). *, nonspecific band.

C Kinetics of siRNA-mediated depletion of ADAR1 and induction of the type I IFN response in HEK293. HEK293 cells stably expressing FLAG-LGP2 were transfected with an siRNA targeting ADAR1 (siADAR1) and harvested at the indicated time points post-transfection. ADAR1 knockdown and IFIT1 upregulation were monitored by RT-qPCR analysis (using Taqman probes) and normalized to ACTB. Data are means ± s.d. from one experiment.

D HEK293 WT cells were transfected with siADAR1 or a control siRNA (siCtrl) and 8 h later with increasing amounts of a vector encoding FLAG-LGP2. As a control, cells were transfected with 250 ng of an empty vector (EV). Cells were harvested 80 h post siRNA transfection. RT-qPCR analysis was used to monitor IFN-β and IFIT1 expression, ADAR1 knockdown, and LGP2 (*DHX58*) expression. All transcripts were normalized to ACTB. Data are means ± s.d. from a representative of two biological replicate experiments.

E Cells were treated as in (D). Protein lysates were prepared and analyzed by SDS-PAGE followed by immunoblotting using the indicated antibodies.

Source data are available online for this figure.

Figure EV2. LGP2-deficient cells fail to sense unedited self RNAs, yet maintain the ability to detect viral dsRNAs, related to Fig 1.

A WT and LGP2-knockout (clones 1 and 2) HEK293 cells were transfected with an siRNA targeting ADAR1 (siADAR1) or a control siRNA (siCtrl) and were treated 8 h later with recombinant type I IFN to upregulate RLR expression. Cells were harvested 80 h post siRNA transfection and RT-qPCR analysis was used to monitor IFN-β and IFIT1 expression and ADAR1 knockdown. All transcripts were normalized to ACTB. Data are means ± s.d. from a representative of three biological replicate experiments.

B Cells were treated as in (A). Protein lysates were prepared 80 h post siRNA transfection, followed by SDS-PAGE and immunoblotting using the indicated antibodies ($n = 3$). siC, siCtrl; siA, siADAR1.

C LGP2-knockout (clones 1 and 2) HEK293 cells were transfected with siADAR1 or siCtrl and 8 h later with a vector encoding FLAG-LGP2 or an empty vector (EV). Cells were harvested 80 h post siRNA transfection and RT-qPCR analysis was used to monitor IFN-β and IFIT1 expression and ADAR1 knockdown. All transcripts were normalized to ACTB. Data are means ± s.d. from a representative of two biological replicate experiments.

D Cells were treated as in (C). Protein lysates were prepared 80 h post siRNA transfection, followed by SDS-PAGE and immunoblotting using the indicated antibodies ($n = 2$).

E, F WT, LGP2-knockout (clones 1 and 2), and stably expressing FLAG-LGP2 HEK293 cells were transfected with transfection reagent only (LF2000), poly(I:C) (56, 112, 225, or 450 ng in (E)), or RNA isolated from HEK293 cells infected with EMCV in the presence of ribavirin (450 or 900 ng in (F)). Cells were harvested 16 h post-transfection and RT-qPCR analysis was used to monitor IFN-β and IFIT1 expression. All transcripts were normalized to ACTB. Data are means ± s.d. from a representative of four (E) or three (F) biological replicate experiments.

G MDA5-knockout HEK293 cells stably expressing FLAG-LGP2 or an empty vector (EV) were transfected with increasing amounts (5, 20, 40, 80, or 240 ng/ml) of a vector encoding FLAG-MDA5 WT or FLAG-MDA5 G495R. As a control, cells were transfected with 240 ng/ml control vector or left untreated. Cells were harvested 24 h post-transfection and RT-qPCR analysis was used to monitor IFN-β and IFIT1 expression. All transcripts were normalized to ACTB. Data are means ± s.d. from a representative of two biological replicate experiments.

Source data are available online for this figure.

 © 2022 The Authors

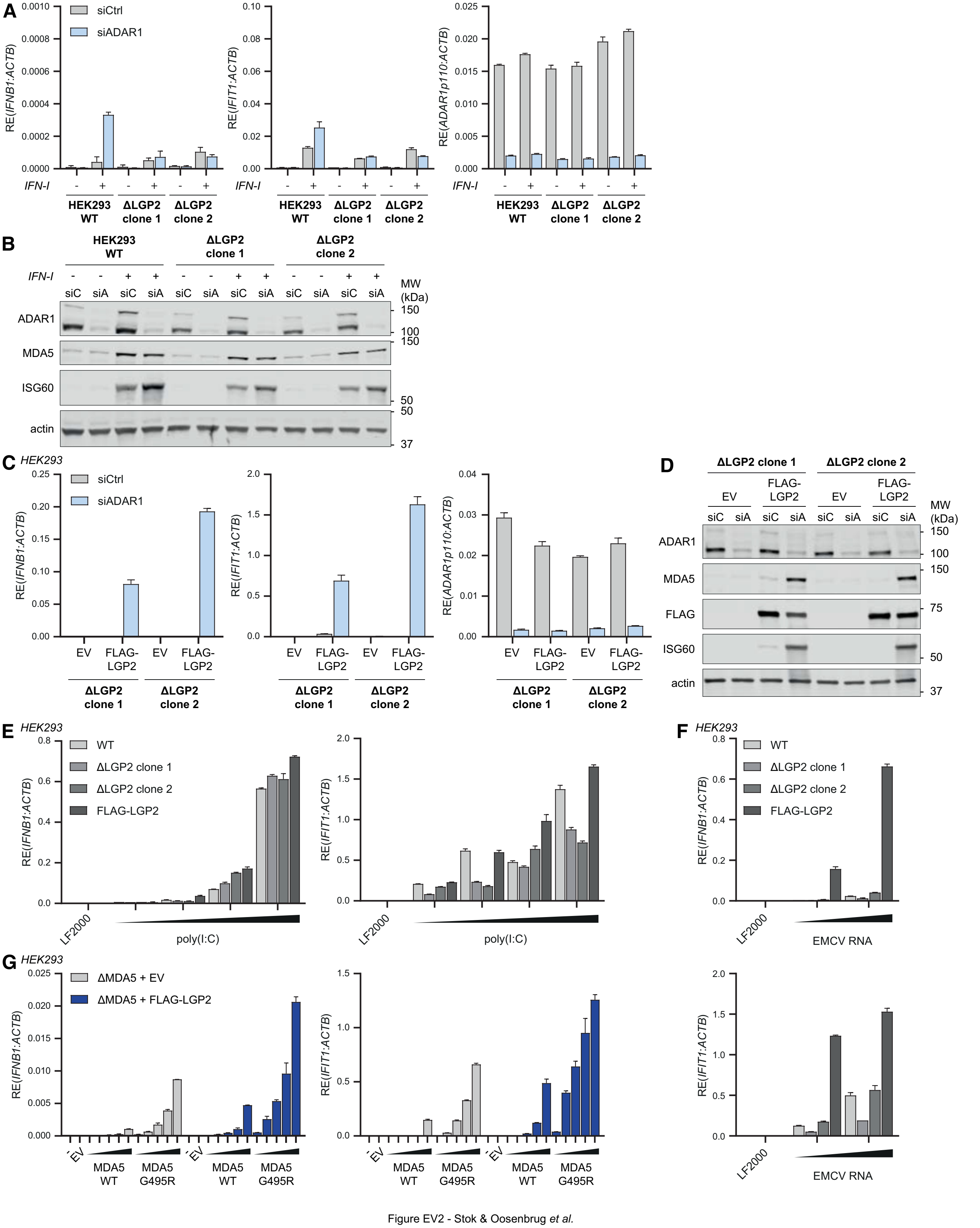
A
siCtrl
siADAR1
RE(IFNB1:ACTB)
RE(IFIT1:ACTB)
RE(ADAR1p110:ACTB)
IFN-I
HEK293 WT
ΔLGP2 clone 1
ΔLGP2 clone 2
B
IFN-I
siC siA
MW (kDa)
ADAR1
MDA5
ISG60
actin
150
100
50
37
C
HEK293
EV
FLAG-LGP2
D
ΔLGP2 clone 1
ΔLGP2 clone 2
FLAG
75
E
WT
ΔLGP2 clone 1
ΔLGP2 clone 2
FLAG-LGP2
LF2000
poly(I:C)
F
EMCV RNA
G
ΔMDA5 + EV
ΔMDA5 + FLAG-LGP2
EV
MDA5 WT
MDA5 G495R
Figure EV2 - Stok & Oosenbrug et al.

Figure EV2.

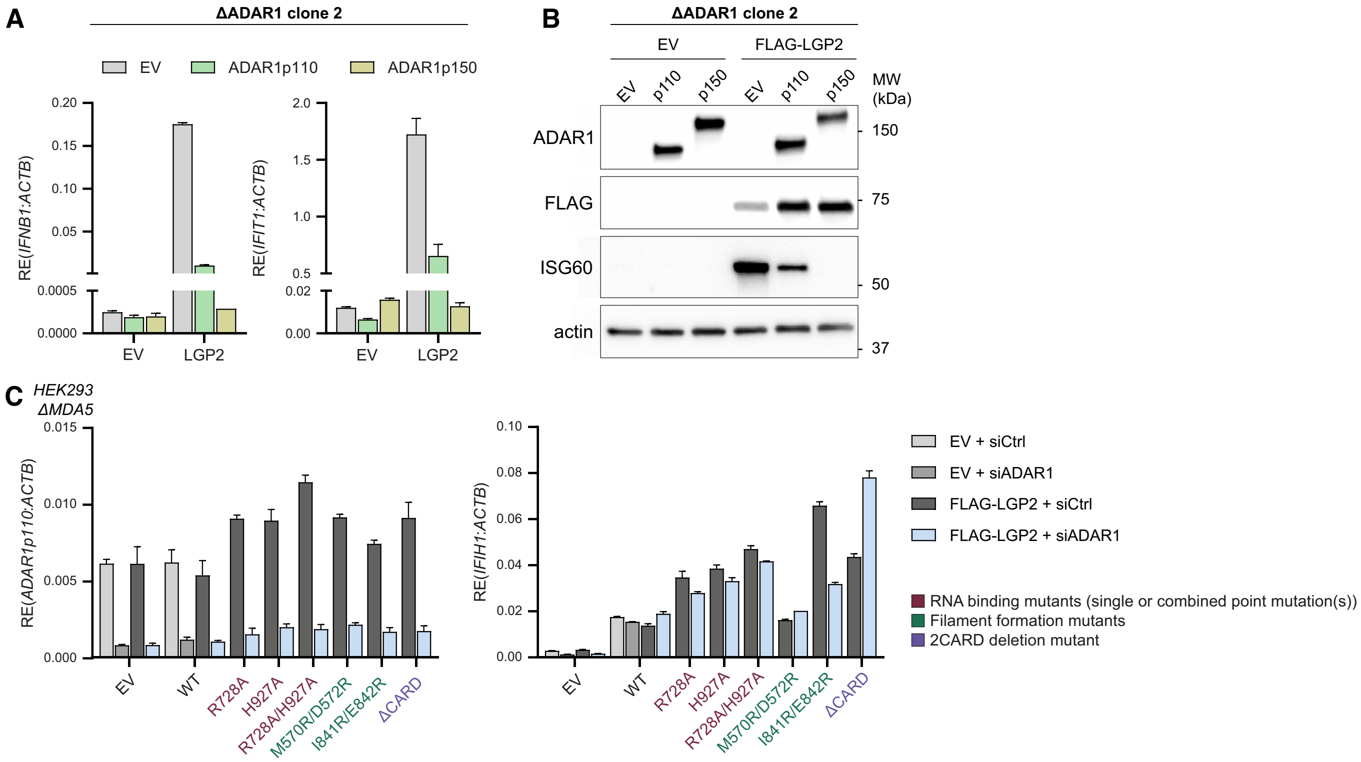

Figure EV3. A type I IFN response is unleashed in ADAR1-knockout cells upon expression of LGP2, related to Figs 2 and 3.

A ADAR1-knockout HEK293 cells (clone 2) were cotransfected with an empty vector (EV) or a FLAG-LGP2-encoding vector (LGP2) combined with a vector encoding GFP-tagged ADAR1 p110 or p150. Cells were harvested 48 h post-transfection and the type I IFN response was monitored by measuring IFN-β and IFIT1 transcript expression, relative to ACTB expression, by RT-qPCR. Data are means ± s.d. from a representative of three biological replicate experiments.

B ADAR1-knockout HEK293 cells (clone 2) were transfected as in (A). Protein lysates were analyzed by SDS-PAGE followed by immunoblotting using the indicated antibodies (n = 3).

C MDA5-knockout HEK293 cells, generated in Fig 1D, were transfected with an ADAR1-targeting siRNA (siADAR1) or a control siRNA (siCtrl) and 8 h later with an empty vector (EV) or a vector encoding the indicated WT, truncation, or point mutant(s) of MDA5. Cells were harvested 72 h post-siRNA transfection and RT-qPCR analysis was used to monitor ADAR1 knockdown and MDA5 (*IFIH1*) expression. All transcripts were normalized to ACTB. Data are means ± s.d. from a representative of two biological replicate experiments.

Source data are available online for this figure.

Figure EV4. Loss of ADAR1 inhibits tumor cell growth in an LGP2-dependent manner, related to Fig 5.

A *ADAR*^{low} patients with concomitant *DHX58*^{high} expression have prolonged survival across multiple cancer types. Hazard ratios and 95% confidence intervals from univariate Cox regression models for *DHX58* stratification in *ADAR*^{low} (left panel) and *ADAR*^{high} (right panel) patients from 17 TCGA datasets (sarcoma = SARC, liver = LIHC, esophageal = ESCA, breast = BRCA, bladder = BLCA, endometrial = UCEC, rectal = READ, cervical = CESC, melanoma = SKCM, ovarian = OV, pancreas = PAAD, lung adenocarcinoma = LUAD, stomach = STAD, head and neck = HNSC, clear cell renal cell carcinoma = KIRC, lung squamous = LUSC, colon = COAD). Median cut-off values for both *ADAR* and *DHX58* were used for patient stratification. Dashed lines indicate a hazard ratio of 1. Wald test *P* values are shown.

B–D CAL27 cells were transduced with doxycycline-inducible shRNAs targeting ADAR1 or GFP (negative control) and subsequently treated with doxycycline and/or transfected with two independent siRNAs targeting LGP2 (siLGP2 #1 or #2) or a control siRNA (siCtrl). (B) Cells were harvested 72 h post-treatment and RT-qPCR analysis was used to monitor knockdown efficiency of ADAR1 and LGP2 (*DHX58*) upon doxycycline treatment or siRNA transfection, respectively. All transcripts were normalized to ACTB. Data are means ± s.d. from a representative of three biological replicate experiments. (C) Cell confluency was measured every 4 h on a IncuCyte S3 Live-Cell Analysis machine. Data are means ± s.e.m (16 replicate fields of view per experimental condition) from a representative of two biological replicate experiments. (D) Knockdown efficiency of ADAR1 and LGP2 (*DHX58*) in samples of (C) at 120 h post-treatment was determined as in (B). Data are means ± s.d. from one experiment.

© 2022 The Authors

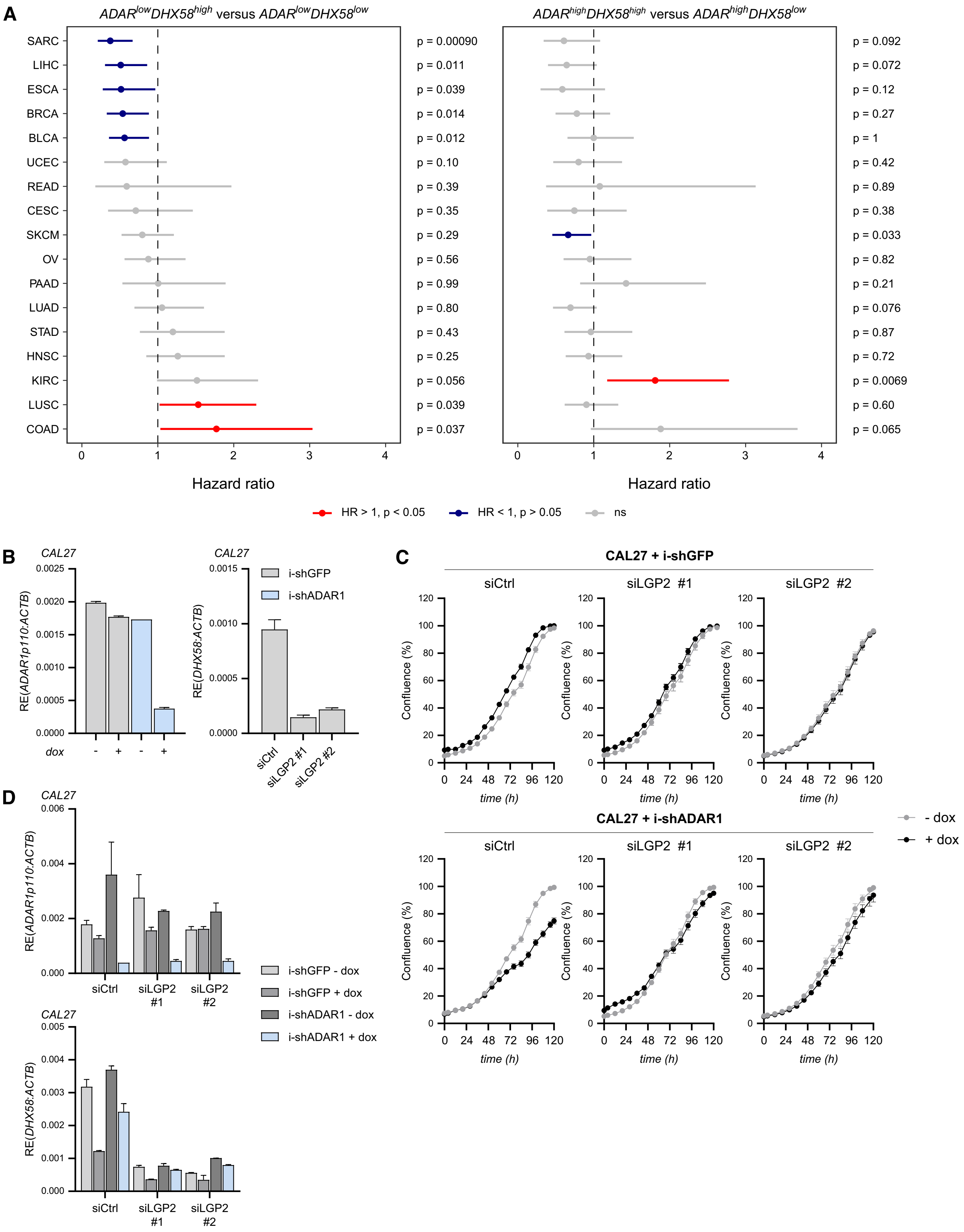
A
ADAR^low DHX58^high versus ADAR^low DHX58^low
ADAR^high DHX58^high versus ADAR^high DHX58^low
SARC
LIHC
ESCA
BRCA
BLCA
UCEC
READ
CESC
SKCM
OV
PAAD
LUAD
STAD
HNSC
KIRC
LUSC
COAD
p = 0.00090
p = 0.011
p = 0.039
p = 0.014
p = 0.012
p = 0.10
p = 0.39
p = 0.35
p = 0.29
p = 0.56
p = 0.99
p = 0.80
p = 0.43
p = 0.25
p = 0.056
p = 0.039
p = 0.037
p = 0.092
p = 0.072
p = 0.12
p = 0.27
p = 1
p = 0.42
p = 0.89
p = 0.38
p = 0.033
p = 0.82
p = 0.21
p = 0.076
p = 0.87
p = 0.72
p = 0.0069
p = 0.60
p = 0.065
Hazard ratio
HR > 1, p < 0.05
HR < 1, p > 0.05
ns
B
CAL27
RE(ADAR1p110:ACTB)
dox
RE(DHX58:ACTB)
i-shGFP
i-shADAR1
siCtrl
siLGP2 #1
siLGP2 #2
C
CAL27 + i-shGFP
CAL27 + i-shADAR1
siCtrl
siLGP2 #1
siLGP2 #2
Confluence (%)
time (h)
- dox
+ dox
D
CAL27
RE(ADAR1p110:ACTB)
RE(DHX58:ACTB)
i-shGFP - dox
i-shGFP + dox
i-shADAR1 - dox
i-shADAR1 + dox

Figure EV4.

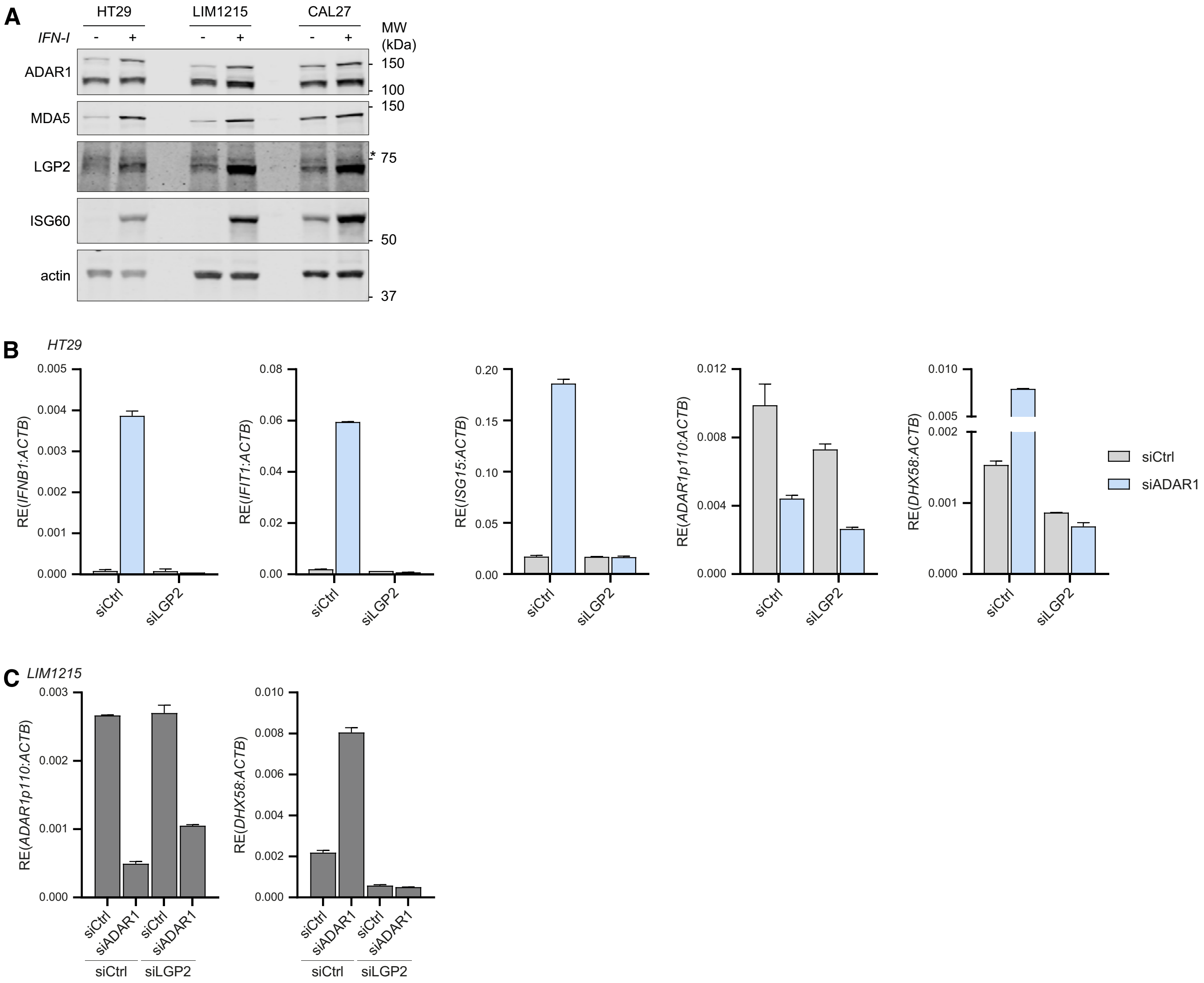

Figure EV5. Type I IFN responsiveness and siADAR1-dependent type I IFN induction in various cancer cell lines, related to Figs 5 and 6.

A Endogenous expression level and type I IFN inducibility of relevant proteins in HT29, LIM1215, and CAL27 cells. Cells were treated with or without recombinant type I IFN. Protein lysates were analyzed by SDS-PAGE followed by immunoblotting with the indicated antibodies. *, nonspecific band.

B HT29 cells were transfected with the indicated siRNAs. Cells were harvested 72 h post-transfection and RT-qPCR analysis was used to monitor the type I IFN response (IFN-β, IFIT1, and ISG15 transcripts) and knockdown efficiency of ADAR1 and LGP2 (*DHX58*). All transcripts were normalized to ACTB. Data are means ± s.d. from a representative of three biological replicate experiments.

C LIM1215 cells were transfected with the indicated siRNAs. Cells were harvested 72 h post-transfection and knockdown efficiency of ADAR1 and LGP2 (*DHX58*) was analyzed as in (C). Data are means ± s.d. from a representative of three biological replicate experiments.

Source data are available online for this figure.

© 2022 The Authors
