# Supplementary material for: RNA sensing via the RIG‐I‐like receptor LGP2 is essential for the induction of a type I IFN response in ADAR1 deficiency
Source: EMBO J. 2022 Feb 14;41(6):e109760. doi: 10.15252/embj.2021109760 (PMC8922249; doi:10.15252/embj.2021109760)
Supplement: Supplementary file 2 — Source Data for Expanded View [file EMBJ-41-e109760-s007.zip › EV_Figure_Source_Data/EMBOJ-2021-109760R1-Figure_EV1_Source_Data-sd.pdf]

Source Data Figure EV1

A)

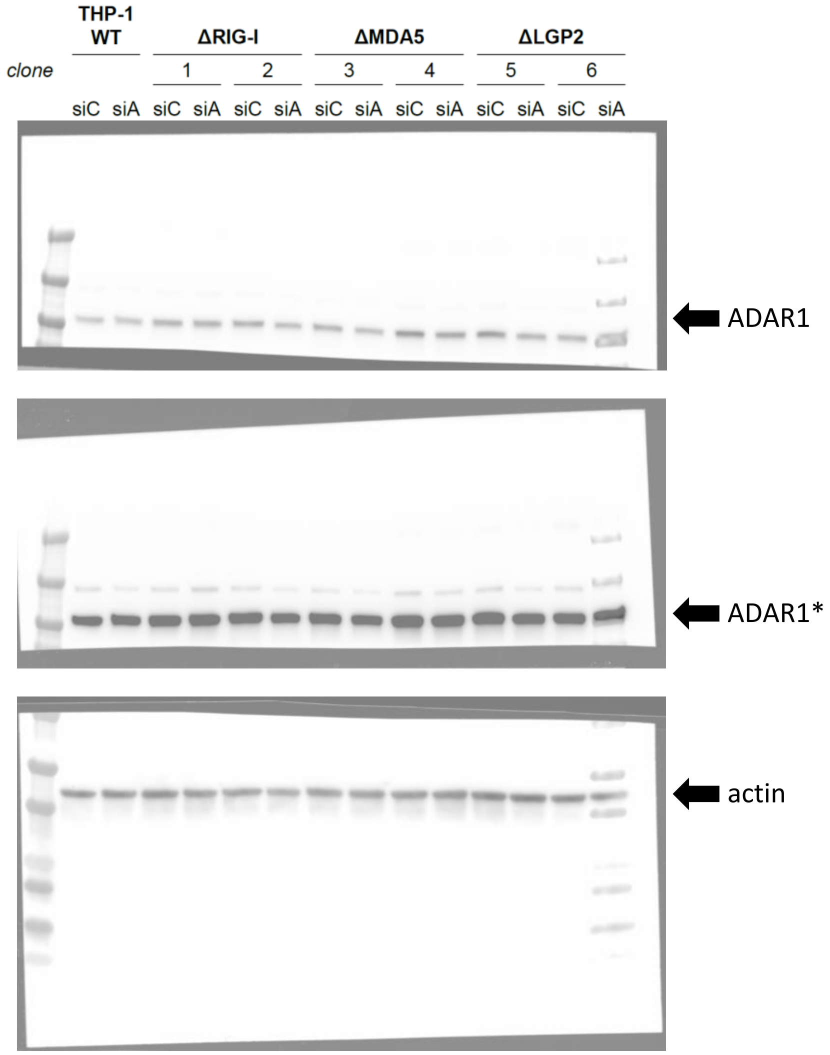

* Different exposure of the same membrane as shown above

Multichannel ChemiDox XP images (chemiluminescence & colorimetric) are presented, whereas the corresponding single channel chemiluminescence images were used for the final figures.

Source Data Figure EV1

B)

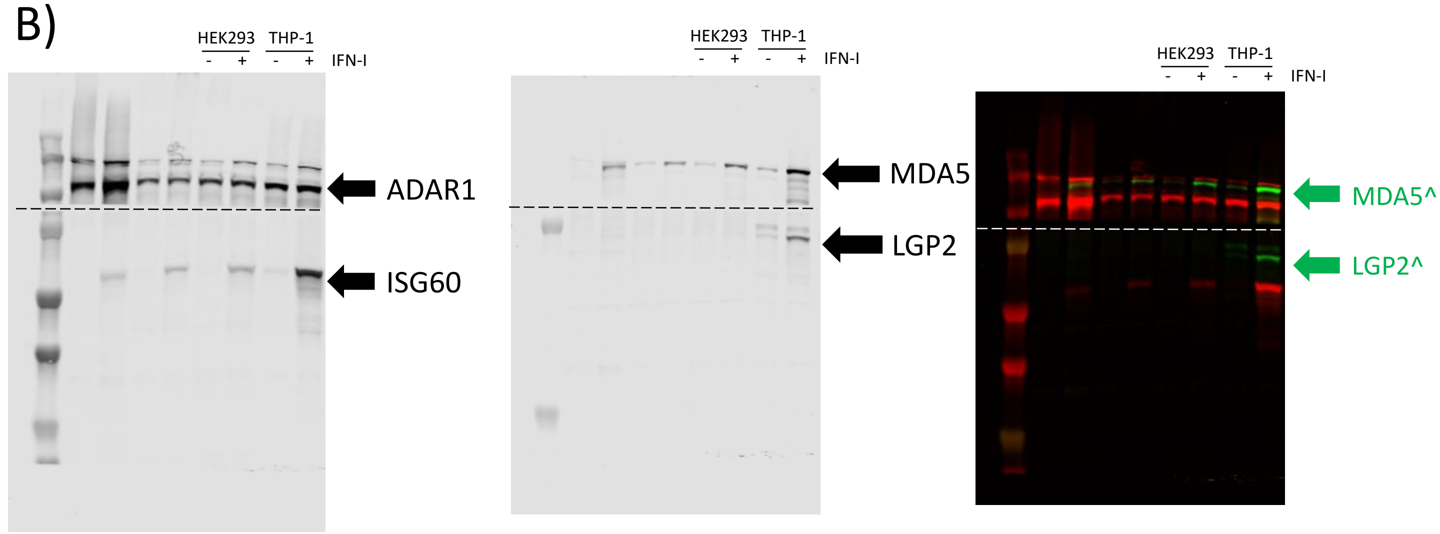

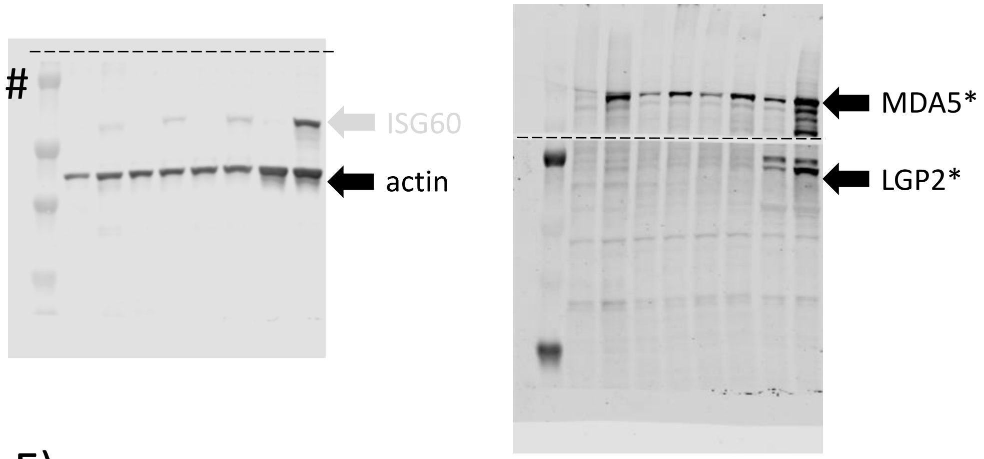

# only the **bottom** part of the cut membrane was imaged
* Different exposure of the same membrane as shown above
^ Dual color view of the membrane shown on the left for better visualization of the protein standard

E)

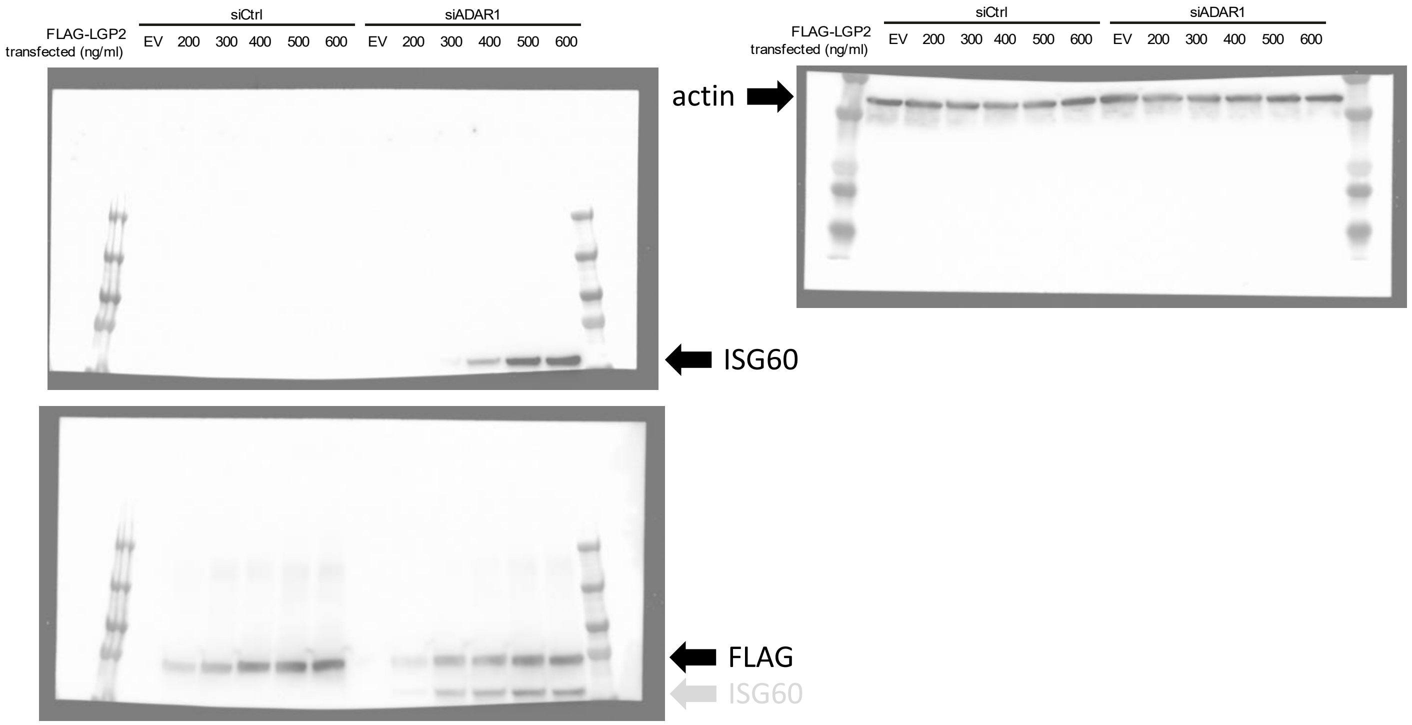

Grey arrows indicate protein bands that are visible from a previous round of antibody staining. Dotted lines indicate where membrane has been cut horizontally prior to staining. Multichannel ChemiDox XP images (chemiluminescence & colorimetric) are presented, whereas the corresponding single channel chemiluminescence images were used for the final figures.
