# Supplementary material for: RNA sensing via the RIG‐I‐like receptor LGP2 is essential for the induction of a type I IFN response in ADAR1 deficiency
Source: EMBO J. 2022 Feb 14;41(6):e109760. doi: 10.15252/embj.2021109760 (PMC8922249; doi:10.15252/embj.2021109760)
Supplement: Supplementary file 2 — Source Data for Expanded View [file EMBJ-41-e109760-s007.zip › EV_Figure_Source_Data/EMBOJ-2021-109760R1-Figure_EV2_Source_Data-sd.pdf]

Source Data Figure EV2

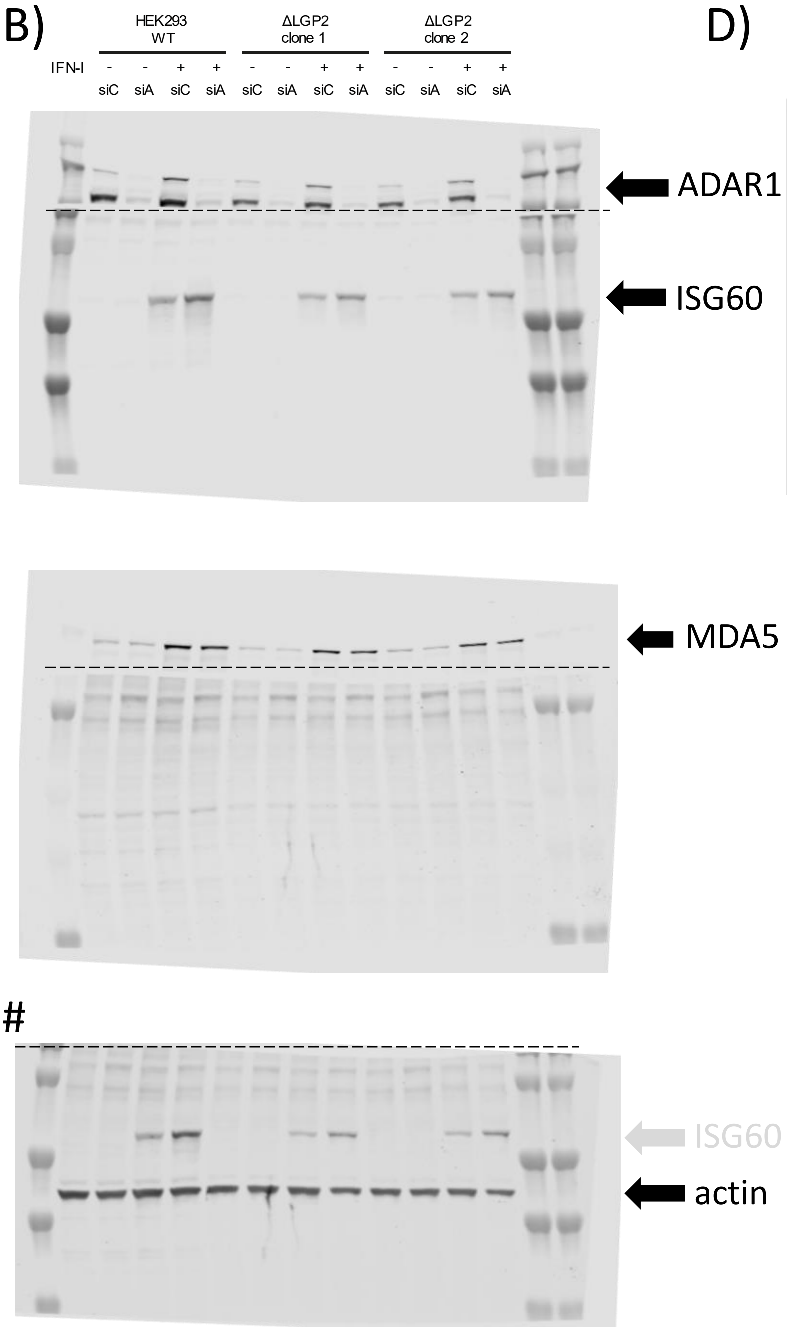

only the **bottom** part of the cut membrane

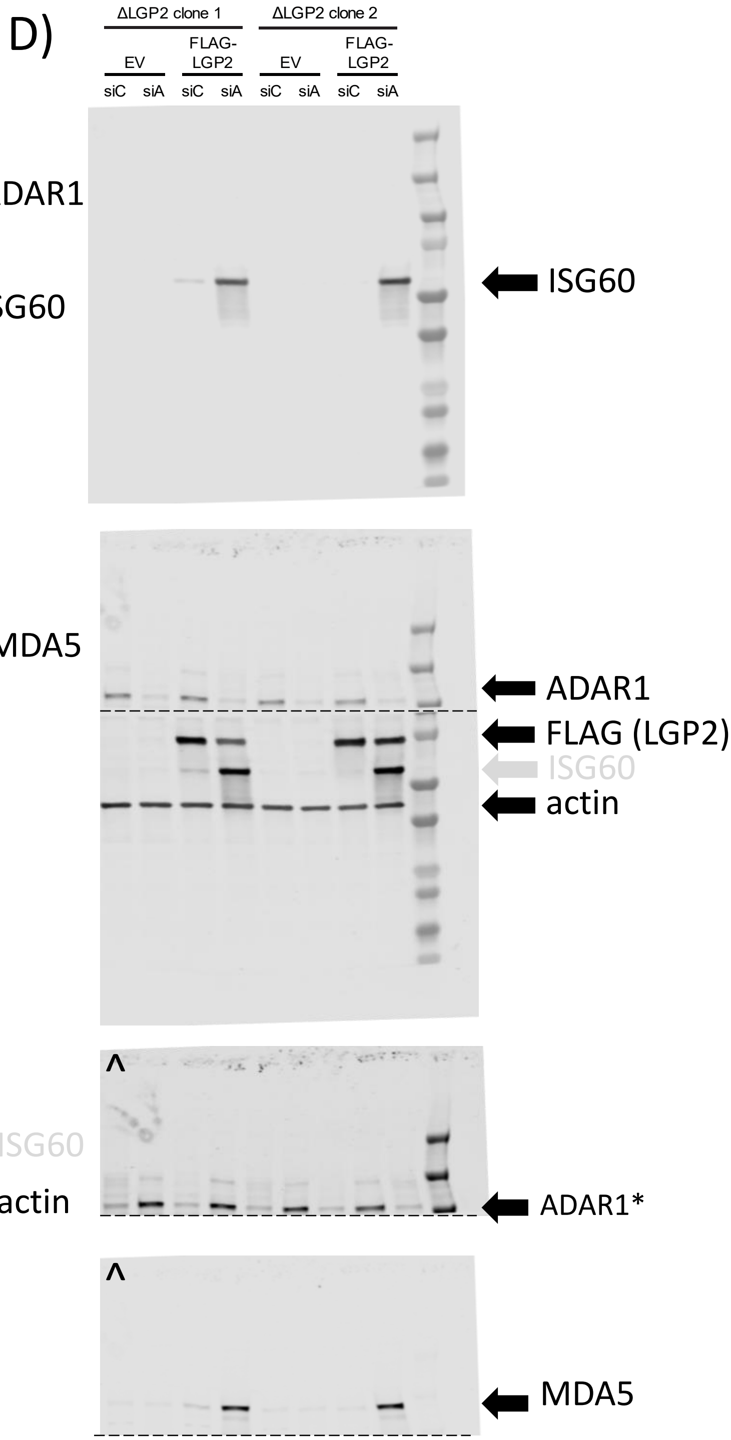

^only the **top** part of the cut membrane

* different exposure of the same **top** part of the cut membrane shown above

Grey arrows indicate protein bands that are visible from a previous round of antibody staining.
Dotted lines indicate where membrane has been cut horizontally prior to staining.
