# Supplementary material for: RNA sensing via the RIG‐I‐like receptor LGP2 is essential for the induction of a type I IFN response in ADAR1 deficiency
Source: EMBO J. 2022 Feb 14;41(6):e109760. doi: 10.15252/embj.2021109760 (PMC8922249; doi:10.15252/embj.2021109760)
Supplement: Supplementary file 3 — Source Data for Figure 1 [file EMBJ-41-e109760-s001.pdf]

Source Data Figure 1

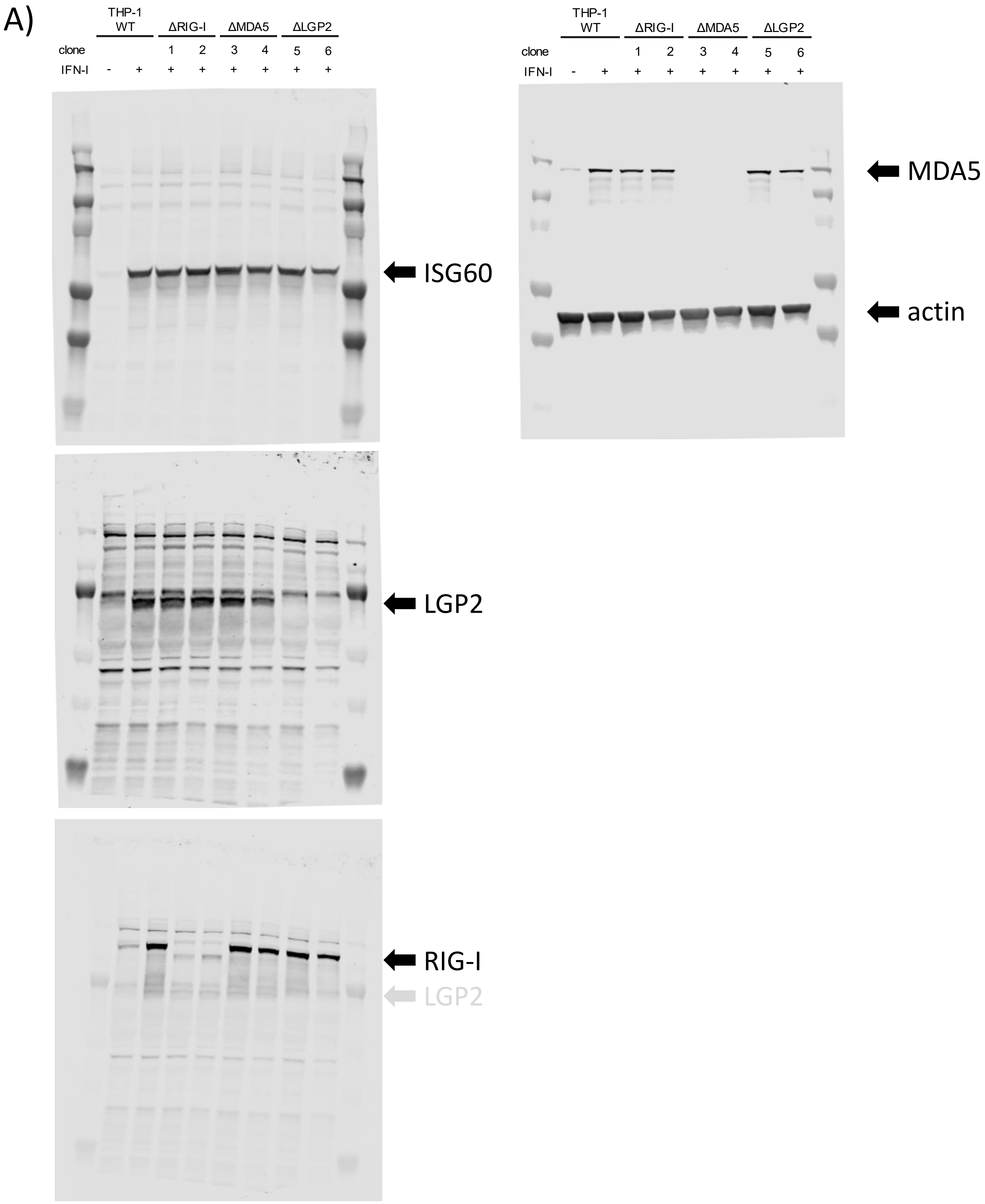

Grey arrows indicate protein bands that are visible from a previous round of antibody staining.

Source Data Figure 1

D)

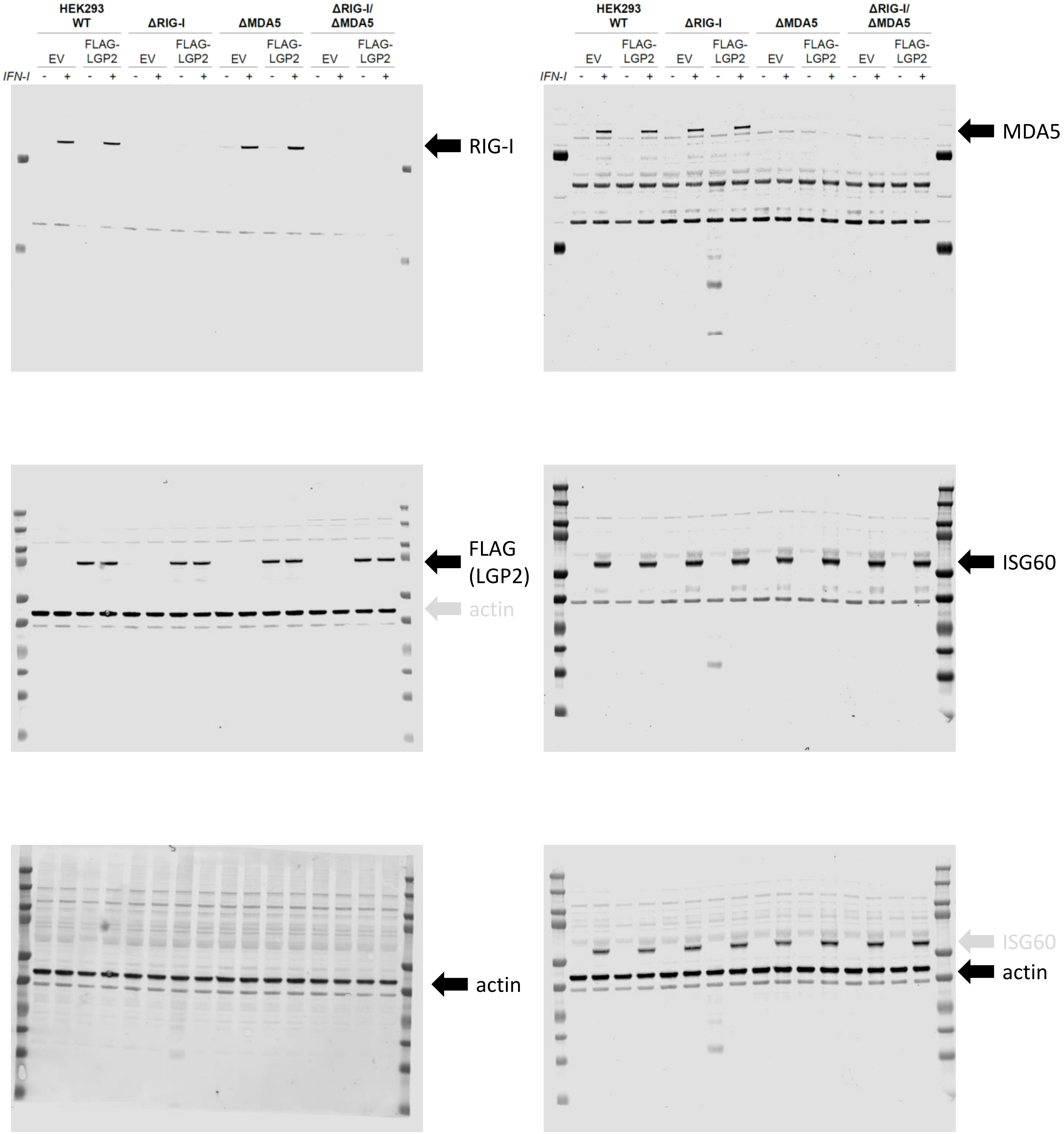

Grey arrows indicate protein bands that are visible from a previous round of antibody staining.

Source Data Figure 1

F)

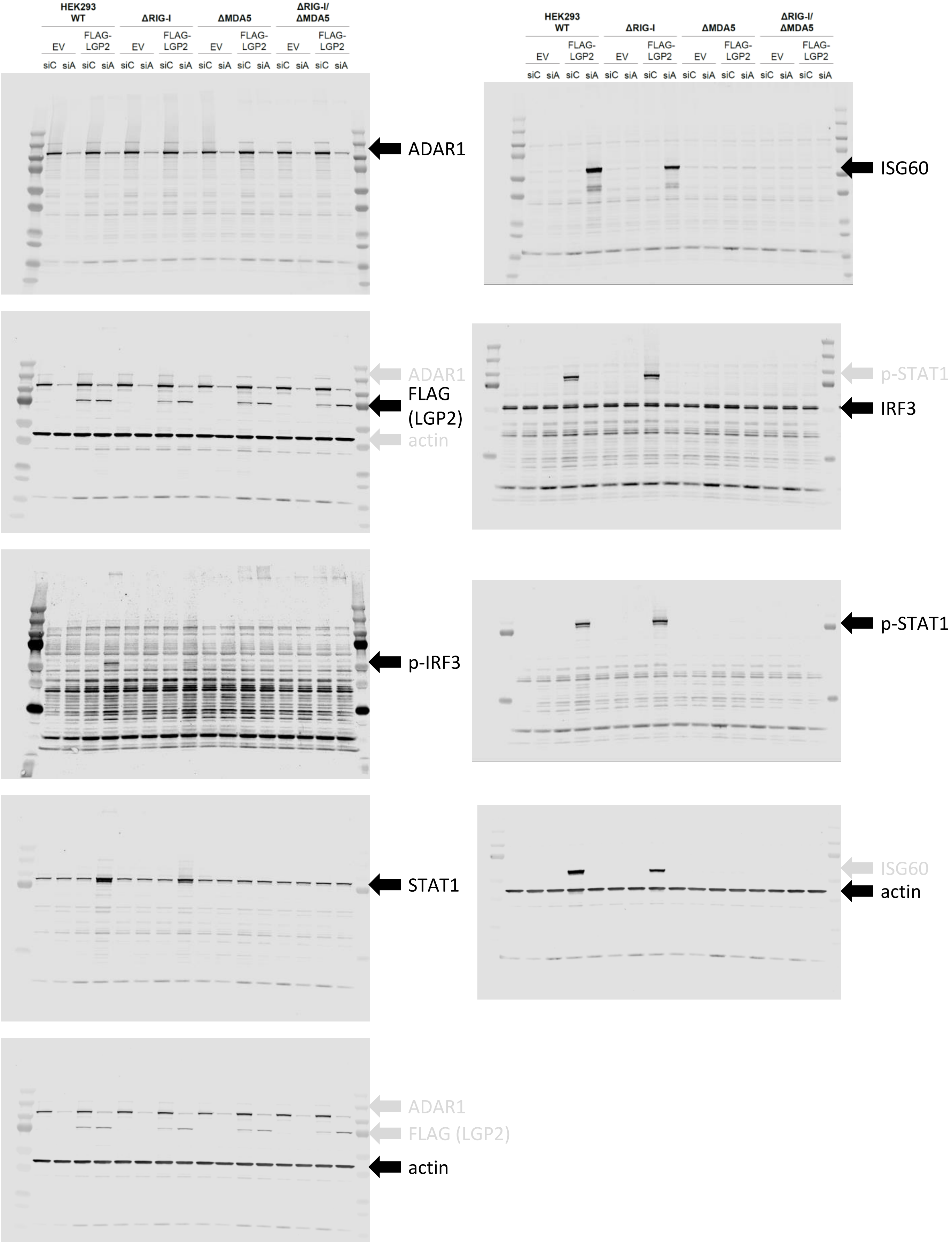

Grey arrows indicate protein bands that are visible from a previous round of antibody staining.
