# Supplementary material for: RNA sensing via the RIG‐I‐like receptor LGP2 is essential for the induction of a type I IFN response in ADAR1 deficiency
Source: EMBO J. 2022 Feb 14;41(6):e109760. doi: 10.15252/embj.2021109760 (PMC8922249; doi:10.15252/embj.2021109760)
Supplement: Supplementary file 6 — Source Data for Figure 4 [file EMBJ-41-e109760-s005.pdf]

Source Data Figure 4

C)

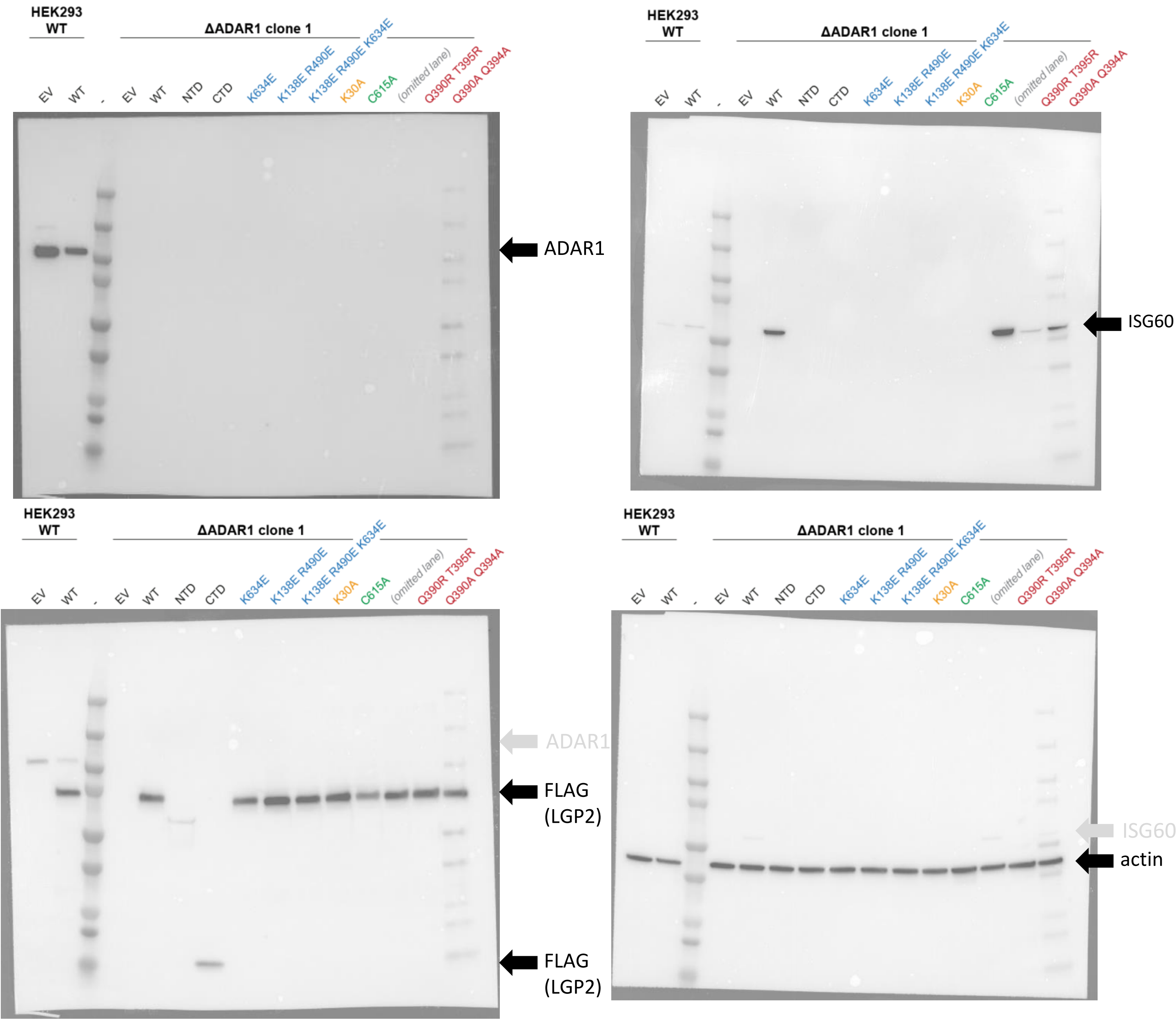

Note: lane #13 was omitted from the final figure. For the final figure, the dotted lines indicate the juxtaposition of the two adjacent lanes (#12 and #14).

Grey arrows indicate protein bands that are visible from a previous round of antibody staining. Multichannel ChemiDox XP images (chemiluminescence & colorimetric) are presented, whereas the corresponding single channel chemiluminescence images were used for the final figures.
