# Supplementary material for: RNA sensing via the RIG‐I‐like receptor LGP2 is essential for the induction of a type I IFN response in ADAR1 deficiency
Source: EMBO J. 2022 Feb 14;41(6):e109760. doi: 10.15252/embj.2021109760 (PMC8922249; doi:10.15252/embj.2021109760)
Supplement: Supplementary file 7 [file EMBJ-41-e109760-s003.pdf]

THE EMBO JOURNAL

RNA sensing via LGP2 is essential for the induction of a type I IFN response in ADAR1 deficiency

Jorn Stok, Timo Oosenbrug, Laurens Ter Haar, Dennis Gravekamp, Christian Bromley, Santiago Zelenay, Caetano Reis e Sousa, and Annemarthe Van der Veen
DOI: 10.15252/embj.2021109760

Corresponding author(s): Annemarthe Van der Veen (A.G.van_der_Veen@lumc.nl)

Review Timeline:

Submission Date:	20th Sep 21
Editorial Decision:	13th Oct 21
Revision Received:	24th Nov 21
Editorial Decision:	5th Jan 22
Revision Received:	7th Jan 22
Accepted:	11th Jan 22

Editor: Karin Dumstrei

Transaction Report:

(Note: With the exception of the correction of typographical or spelling errors that could be a source of ambiguity, letters and reports are not edited. Depending on transfer agreements, referee reports obtained elsewhere may or may not be included in this compilation. Referee reports are anonymous unless the Referee chooses to sign their reports.)

1st Editorial Decision **13th Oct 2021**

Dear Annemarthe,

Thank you for submitting your manuscript to The EMBO Journal. Your manuscript has now been seen by three referees and their comments are provided below.

As you can see from the reports, the referees appreciate the analysis. However, they also raise different concerns with the analysis that should be addressed to consider publication here. Should you be able to address the concerns raised in full then I would like to invite a revised version. I think it would be helpful to discuss the raised points further and I am available to do so via email or video.

When preparing your letter of response to the referees' comments, please bear in mind that this will form part of the Review Process File, and will therefore be available online to the community. For more details on our Transparent Editorial Process, please visit our website: https://www.embopress.org/page/journal/14602075/authorguide#transparentprocess

I have attached a document with helpful tips on how to prepare the revised version. Please pay attention to the parts on the Data Availability Section and figure legends.

Thank you for the opportunity to consider your work for publication. I look forward to discussing the revisions further with you

with best wishes

Karin

Karin Dumstrei, PhD
Senior Editor
The EMBO Journal

Information is available in our Guide For Authors: https://www.embopress.org/page/journal/14602075/authorguide

Revision to The EMBO Journal should be submitted online within 90 days - let us know if you need more time and we can grant an extension. Please click on the link below to submit the revision online before 11th Jan 2022:

https://emboj.msubmit.net/cgi-bin/main.plex

Referee #1:

In this manuscript, Stok et al used various cellular assays to show that LGP2 plays a key role in MDA5-mediated auto-inflammation. These include depletion of ADAR1 or treatment with chemotherapeutic agents, such as 5-AZA-cytidine. Overall, the data are convincing and supportive of the main conclusion. I have a few suggestions that could further improve the manuscript.

1. The authors showed that MDA5 activation under the ADAR1 deficiency is completely dependent on LGP2, but argued that LGP2 is dispensable for MDA5's response to polyIC or RNA derived from EMCV-infected cells. In order to support this argument, Fig S2E needs a side-by-side comparison between WT and LGP2 KO, and between with and without LGP2 complementation in the KO clones.

2. The authors argue that MDA5 activation in most auto-inflammatory conditions requires (or depends on) LGP2. However, the authors used just 2-3 conditions (ADAR1 KO, 5-AZA-cytidine, ADAR1+Palbociclib) where MDA5 auto inflammation has previously been documented. To be more complete, I recommend the authors to examine additional conditions that are highly physiologically relevant - (a) gain-of-function MDA5 mutation, (b) deficiency of the mitochondrial PNPase. Self RNAs that trigger MDA5 are supposed to be different in these two conditions, and the findings (regardless of the result) would potentially provide clues for the mechanism for the LGP2 dependence.

3. WB for ADAR1 KD in THP1 cells is missing, and should be shown in Fig 1.

Referee #2:

Distinction between self and non-self is absolutely critical. Aberrant recognition of self-ligands by pattern recognition receptors e.g. of the RLR family (RIG-I, MDA5 and LGP2) leads to auto-immune/auto-inflammatory diseases. After recognition of their ligands, two RLR members RIG-I and MDA5 mediate downstream signaling with their CARD domains, whereas the third member, LGP2, lacks the CARD domains is considered to regulate RIG-I and MDA5.
One of the cellular mechanisms to avoid self-recognition are RNA modifications e.g. by Adenosin deaminase Acting on RNA (ADAR1) that allow host RNAs to escape detection by immune sensors. Consequently, ADAR1 loss-of-function leads to the auto-immune diseases, e.g. Aicardi-Goutières syndrome.
In this manuscript Stok & Oosenbrug et al. show that LGP2 is required for self RNA sensing in ADAR1-deficient human cells via MDA5. Accordingly, ADAR1-deficient cells are not able to imitate a type I IFN response in the absence of LGP2 (or in the absence of MDA5). LGP2 truncation and point mutation experiments show that this depends on the function of LGP2 to sense RNA and amplify MDA5 signaling. Further, cell proliferation inhibition and type I IFN response of ADAR1-depleted tumor cells depends on LGP2.

In summary, this manuscript provides novel insight into the function of LGP2 in sensing of self RNA and its potential role in cancer therapy. This is a well-conducted, interesting and relevant molecular biology study and the data present is convincing. I have only a few suggestions how the study could be improved.

Major issues:

One of the major issues of the manuscript is, that evidence in primary cells missing. The authors should either use cells from patients with low ADAR levels, deplete LGP2 and quantify IFN/ISG levels or alternatively use (any) primary cells, deplete LGP2 and/or ADAR1 (siRNA or CRISPR) and show whether autoactivation of innate immunity by ADAR depletion is dependent on LGP2.

I had hoped for a bit more on the connection between LGP2 and MDA5. Can the authors visualise interaction of MDA5 and LGP2 upon ADAR1 depletion. Do these two factors co-localise/co-purify upon ADAR1 kd? Is RNA-binding or are the CARD domains of MDA5 required to mediate LGP2-dependent signalling? The authors should address one of these issues experimentally.

Minor issues:

- LGP2 levels are increased in the presence of ADAR1 (see Fig. 2C, Fig. S3B, Fig. S5C-D), however this effect is not visible in S1D. Can the authors speculate why this may be the case?
- Fig S4: Please quantify the overall survival rate and hazard ratio for all selected cancers and calculate whether there is a significant difference between ADARlowDHX58high and ADARlowDHX58low patients. Can the statement from the manuscript (line 309, 'improved overall survival') be really made? Please rephrase to avoid overinterpretation.
- Fig 4B+C: It seems like the Q390R/T395R mutants has an impact on type I IFN induction. In contrast, the manuscript states that the mutants has no impact on LGP2 ability to induce type I IFN induction (line 288). Please rephrase
- Fig 4B: Please provide a corresponding expression blot
- Fig S5C: Please add an si.LGP2 only control

Referee #3:

In this study by Stok et al, the authors investigate the role of LGP2 in recognizing self-RNA and inducing type I IFN in the absence of ADAR1. Previous studies demonstrate that in the absence of ADAR1, RNA sensor MDA5 recognizes self dsRNAs and activates the production of type I IFN, demonstrating that ADAR1 is important for preventing induction of innate immune responses through the recognition of self RNA (Alu elements). Concurrent deletion of MDA5/ADAR1 abrogates the induction of type I IFN by self dsRNA. A recent study demonstrates that loss of LGP2 rescues the embryonic lethality associated with the ADAR1 Za RNA binding mutant, indicating that LGP2 may be play a role in recognition of self RNA. Here the authors strengthen the role of LGP2 in recognizing self RNA and inducing the production of IFN through its interactions with MDA5. In addition, the authors demonstrate that the RNA binding and ATPase activities of LGP2 are critical for recognition of self RNA and induction of type I IFN. These are novel and interesting findings that demonstrate that higher type I IFN expression in the absence of ADAR1 is due to recognition of self RNA by LGP2 and not MDA5. There are some major concerns, as the majority of the data relies on an artificial overexpression of LGP2 in HEK 293 cells for the observed phenotype. The authors should address the following:

Major concerns:
1. The specificity of siRNA KD/CRIPSR KO should be demonstrated with cDNA complementation experiments.
2. The association of ADAR1/DHX58 in cancer survival is only a correlation and it needs to be functionally associated with induction of IFN through recognition of self RNA via LGP2. Also, the induction of IFN during treatment of ADAR1 KD cells with

anticancer drugs can occur independently of the RLR pathway.
3. This study can be strengthened by comparing the binding efficiencies of LGP2 vs MDA5 to self RNA.
4. As the current model still relies on MDA5 for induction of type I IFN, the authors should perform experiments in ADAR1/LGP2 double KO THP1 cells or another relevant cell line that does not require overexpression of LGP2.
5. Is overexpression of RIG-I or MDA5 sufficient to induce IRF3 localization/IFN responses in the absence of ADAR1?
6. Is the RNA binding function of MDA5 required for LGP2 mediated induction of type I IFN?

Minor concerns:
1. Statistical analysis should be included in all graphs.
2. Fig 1: KD efficiency of ADAR1 proteins in THP1 cells should be included.
3. Fig 1F: There is translocation of IRF3 to the nucleus only in cells expressing low levels of LGP2 but not in high expressing cells. Why?
4. Fig S1A: Induction of ISG60 is low in 293 cells as compared to the data shown in Fig 1C.
5. Fig S2A: Wt controls are missing for IFN treatment groups. Along with IRF3 target genes, Stat1/2 regulated genes should be included as controls for ISG induction in response to IFN treatment.
6. Fig 2C: FLAG-LGP2 expression is lower than other controls.
7. LN172-174: It is not clear what the authors mean by "monitoring p150 RNA levels leads to distorted results".
8. Fig 5B must include some sort of quantitative measurement rather than measurement of crystal violet staining intensity.

Referee #1:

In this manuscript, Stok et al used various cellular assays to show that LGP2 plays a key role in MDA5-mediated auto-inflammation. These include depletion of ADAR1 or treatment with chemotherapeutic agents, such as 5-AZA-cytidine. Overall, the data are convincing and supportive of the main conclusion. I have a few suggestions that could further improve the manuscript.

We thank the reviewer for his/her enthusiasm for our study and for his/her constructive feedback. We have addressed the suggestions point-by-point below.

1. The authors showed that MDA5 activation under the ADAR1 deficiency is completely dependent on LGP2, but argued that LGP2 is dispensable for MDA5's response to polyIC or RNA derived from EMCV-infected cells. In order to support this argument, Fig S2E needs a side-by-side comparison between WT and LGP2 KO, and between with and without LGP2 complementation in the KO clones.

We have now complemented **Fig. EV2E and EV2F** with a side-by-side comparison between WT, LGP2 KO (generated with 2 independent gRNAs), and FLAG-LGP2 expressing HEK293 cells. Cells were transfected with poly(I:C) (**Fig. EV2E**) or with RNA derived from EMCV-infected cells (**Fig. EV2F**). The data demonstrate that type I IFN induction is detectable in LGP2 KO clones yet enhanced by ectopic FLAG-LGP2 expression. Note that we used FLAG-LGP2 expressing HEK293 cells instead of LGP2 KO cells complemented with FLAG-LGP2, as we had performed these experiments while the manuscript was under review.
Furthermore, we have clarified in the text (line 213-219) that we do not argue that LGP2 is *dispensable* for MDA5's response to EMCV RNA, i.e. LGP2 clearly potentiates the response via MDA5. However, it is not *absolutely* required, as is the case in ADAR1 deficiency. Stimulation of LGP2 KO cells with EMCV RNA yields a *modest* type I IFN response, unlike ADAR1 depletion, which completely fails to induce type I IFNs in LGP2 KO cells. In the case of poly(I:C), ectopic FLAG-LGP2 expression has a minimal effect on type I IFN induction, further demonstrating that sensing of viral dsRNA or mimics thereof is less reliant on LGP2.

2. The authors argue that MDA5 activation in most auto-inflammatory conditions requires (or depends on) LGP2. However, the authors used just 2-3 conditions (ADAR1 KO, 5-AZA-cytidine, ADAR1+Palbociclib) where MDA5 auto inflammation has previously been documented. To be more complete, I recommend the authors to examine additional conditions that are highly physiologically relevant - (a) gain-of-function MDA5 mutation, (b) deficiency of the mitochondrial PNPase. Self RNAs that trigger MDA5 are supposed to be different in these two conditions, and the findings (regardless of the result) would potentially provide clues for the mechanism for the LGP2 dependence.

These are interesting suggestions. We have performed the suggested experiments. In the case of an MDA5 gain-of-function mutant (MDA5 G495R*) we find that the autoactivation that is seen upon transfection of this mutant is increased by ectopic LGP2 co-expression, but LGP2 is not strictly required for autoactivation and type I IFN induction by the MDA5 G495R mutant, i.e. the MDA5 G495R mutant can bypass LGP2 (Fig. 1, reviewer only). Note that the same observations are made for MDA5 WT when it is overexpressed.

* Described in: Rice G. et al., Gain-of-function mutations in *IFIH1* cause a spectrum of human disease phenotypes associated with upregulated type I interferon signaling, Nature Genetics 2014.

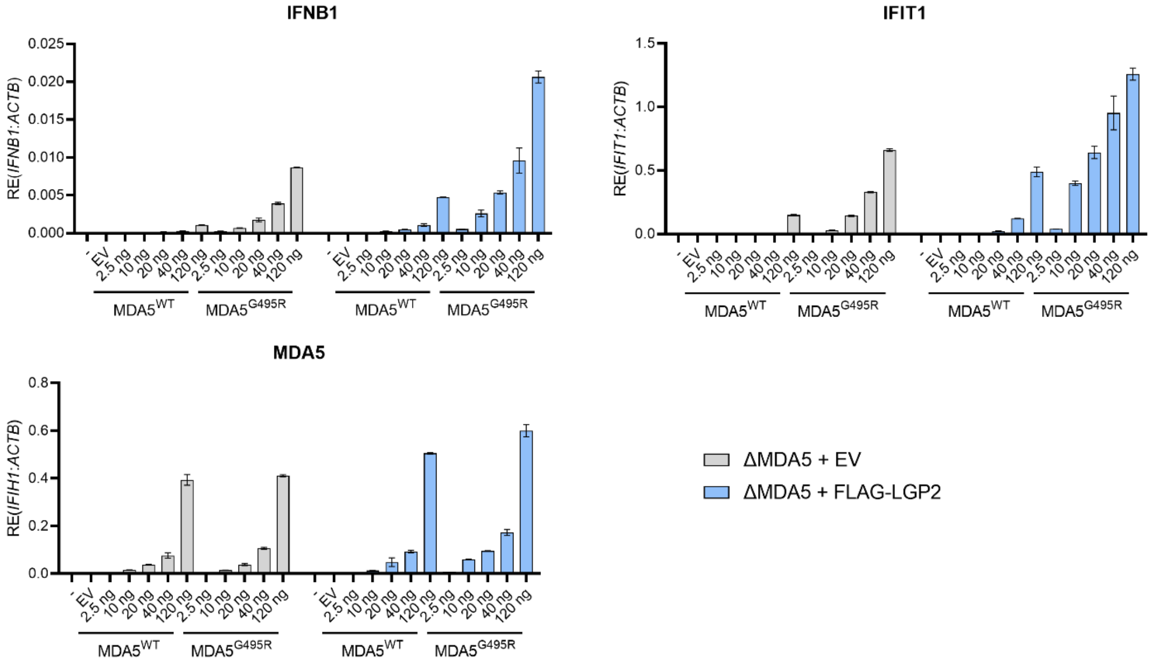

Fig. 1: MDA5 KO HEK293 cells were transduced with FLAG-LGP2 or an empty vector as a control (EV) and subsequently transfected with the indicated doses of MDA5 WT or MDA5 G495R. RNA was extracted and type I IFN induction was monitored by RT-qPCR analysis of IFIT1 and IFN-β transcripts.

With regards to PNPase deficiency, we have knocked down PNPT1 using siRNAs in FLAG-LGP2 expressing HEK293 cells but we failed to see type I IFN induction upon PNPT1 depletion (see Fig. 2, reviewer only). We could therefore not test the impact of LGP2 on PNPase-mediated type I IFN induction in these cells.

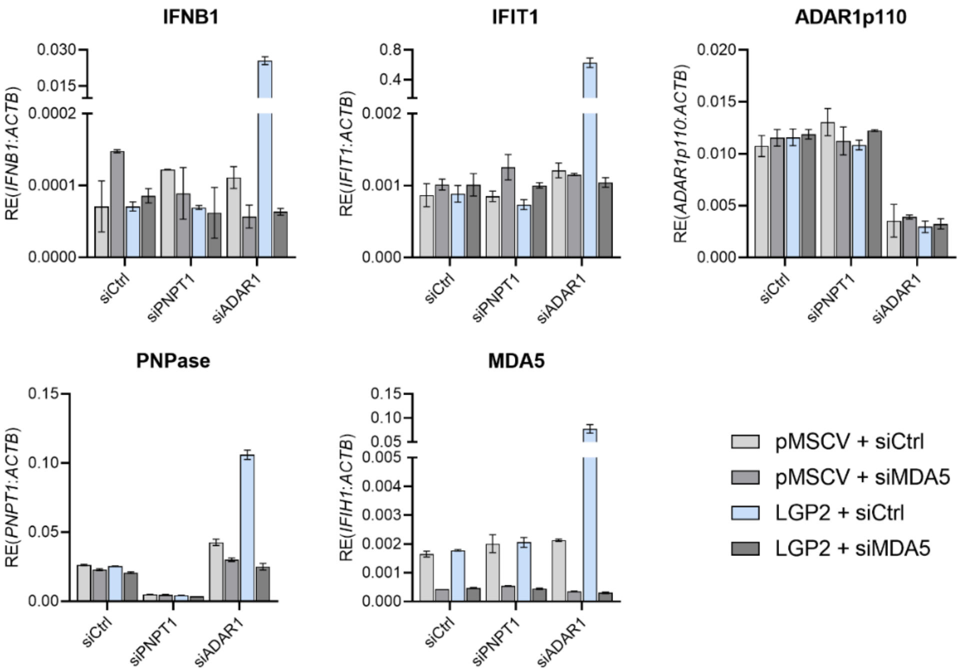

Fig. 2: Knockdown of PNPT1 does not induce a type I IFN response in FLAG-LGP2 expressing HEK293 cells in our experimental set-up.

As we could not investigate the LGP2 dependence in the context of PNPase deficiency in HEK293 cells, we chose *not* to add these data or the data on the MDA5 gain-of-function mutant (Fig. 1, reviewer only) to the manuscript.

3. WB for ADAR1 KD in THP1 cells is missing, and should be shown in Fig 1.

In **Fig. 1B** we demonstrate the knockdown of ADAR1 p110 at *transcript* level, which is a more reliable marker for knockdown efficiency than ADAR1 p150 (due to the IFN-inducible nature of the latter isoform).
At a *protein* level, the knockdown of ADAR1 p150 in THP1 is nearly undetectable at the time point chosen for analysis of the type I IFN response (56 hrs) (Fig. 3, reviewer only). The knockdown of ADAR1 p150 is likely (partially) overcome at this point due to its IFN-inducibility (which explains why the knockdown efficiency appears stronger in the MDA5 KO and LGP2 KO cells that do not induce IFNs). Although it is perhaps disappointing that we cannot clearly demonstrate the knockdown efficiency at *protein* level, the observations do indicate that even a minimal reduction in ADAR1 p150 expression is sufficient to induce a type I IFN response in THP1-derived macrophages.
We did not include the panel below in the (Extended View) figures of the manuscript but we are happy to do so if the reviewer finds this necessary.

In addition to the above, we have now further strengthened our observations regarding the LGP2-dependent type I IFN induction following ADAR1 depletion in **primary human monocyte-derived macrophages**. These data are added as a new panel in **Fig. 1C.** Note that in these cells, ADAR1 was depleted with a different set of siRNAs (ON-TARGETplus siRNA instead of siGenome siRNA), thus providing independent validation of the observed phenotype. The ADAR1 knockdown efficiency in primary macrophages is considerably stronger than in THP1-derived macrophages (measured at transcript level).

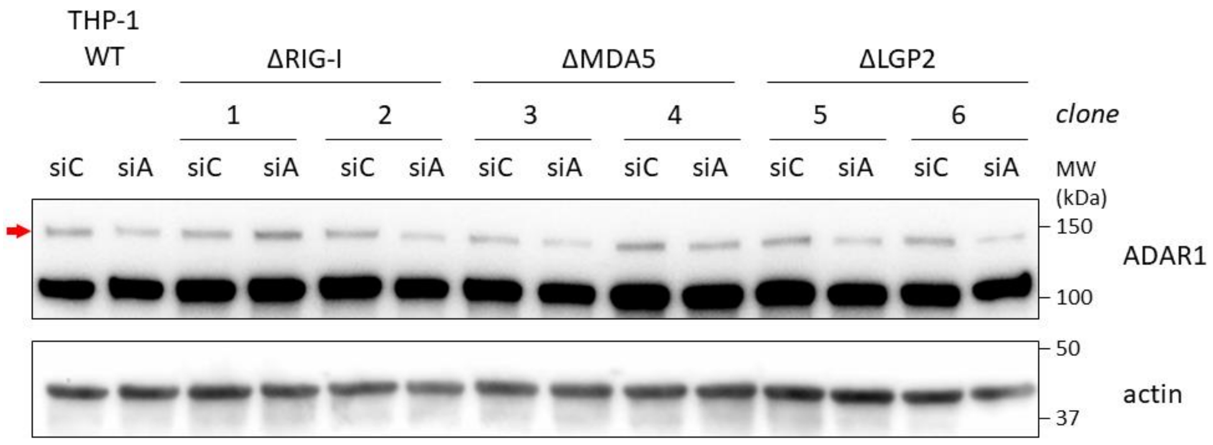

Fig. 3: Knockdown of ADAR1 in THP1-derived macrophages following transfection with control (siC) or ADAR1 (siA) siRNAs. Knockdown of the p150 isoform of ADAR1 is indicated with a red arrow.

Referee #2:
Distinction between self and non-self is absolutely critical. Aberrant recognition of self-ligands by pattern recognition receptors e.g. of the RLR family (RIG-I, MDA5 and LGP2) leads to auto-immune/auto-inflammatory diseases. After recognition of their ligands, two RLR members RIG-I and MDA5 mediate downstream signaling with their CARD domains, whereas the third member, LGP2, lacks the CARD domains is considered to regulate RIG-I and MDA5.
One of the cellular mechanisms to avoid self-recognition are RNA modifications e.g. by Adenosin deaminase Acting on RNA (ADAR1) that allow host RNAs to escape detection by immune sensors. Consequently, ADAR1 loss-of-function leads to the auto-immune diseases, e.g. Aicardi-Goutières syndrome.
In this manuscript Stok & Oosenbrug et al. show that LGP2 is required for self RNA sensing in ADAR1-deficient human cells via MDA5. Accordingly, ADAR1-deficient cells are not able to imitate a type I IFN response in the absence of LGP2 (or in the absence of MDA5). LGP2 truncation and point mutation experiments show that this depends on the function of LGP2 to sense RNA and amplify MDA5 signaling. Further, cell proliferation inhibition and type I IFN response of ADAR1-depleted tumor cells depends on LGP2.

In summary, this manuscript provides novel insight into the function of LGP2 in sensing of self RNA and its potential role in cancer therapy. This is a well-conducted, interesting and relevant molecular biology study and the data present is convincing. I have only a few suggestions how the study could be improved.

We thank the reviewer for his/her positive and constructive feedback. We are pleased to include new data in this revision on the validation of our observations in **primary human monocyte-derived macrophages** and on the connection between LGP2 and MDA5 in ADAR1-deficient cells (see below for details).

Major issues:

One of the major issues of the manuscript is, that evidence in primary cells missing. The authors should either use cells from patients with low ADAR levels, deplete LGP2 and quantify IFN/ISG levels or alternatively use (any) primary cells, deplete LGP2 and/or ADAR1 (siRNA or CRISPR) and show whether autoactivation of innate immunity by ADAR depletion is dependent on LGP2.

We have now further validated our observations regarding the LGP2-dependent type I IFN induction following ADAR1 depletion in **primary human monocyte-derived macrophages**. Monocytes were isolated from PBMCs obtained from 2 donors and differentiated into macrophages using M-CSF. We subsequently transfected these macrophages 7 days post-differentiation with siRNAs targeting ADAR1 and/or LGP2, as described*. Cells were harvested 96 hrs post-transfection and IFN-β, IFIT1, ISG15, ADAR1 p110, and LGP2 transcript levels were determined by qPCR. Consistent with our earlier data and hypothesis, we find that loss of LGP2 markedly reduces type I IFN induction upon ADAR1 depletion. We added these data as a new panel in **Fig. 1C.**

*Troegeler A. et al., An efficient siRNA-mediated gene silencing in primary human monocytes, dendritic cells, and macrophages, Immunol Cell Biol 2014

I had hoped for a bit more on the connection between LGP2 and MDA5. Can the authors visualise interaction of MDA5 and LGP2 upon ADAR1 depletion. Do these two factors co-

localise/co-purify upon ADAR1 kd? Is RNA-binding or are the CARD domains of MDA5 required to mediate LGP2-dependent signalling? The authors should address one of these issues experimentally.

We have focused on the third suggestion of the reviewer. We have depleted ADAR1 through siRNAs and simultaneously expressed various MDA5 mutants in MDA5 KO HEK293 cells that were stably transduced with FLAG-LGP2 or an empty vector (as a control). We expressed limited amounts of MDA5 plasmid, to prevent autoactivation as a result of overexpression. Type I IFN induction was monitored by IFIT1 and ISG15 qPCR. As expected, we find that MDA5 WT is required to convey LGP2-dependent signaling. Instead, expression of MDA5 mutants that have impaired capacity to form filaments (M570R/D572R, I841R/E842R)* or to bind RNA (R728A, H927A, R728A/H927A)* or that lack the CARD domains completely (MDA5ΔCARD), all fail to induce a type I IFN response in ADAR1-depleted cells. We conclude that LGP2-dependent type I IFN induction requires signaling, filament formation, and RNA binding by MDA5. We have included these new data as **Fig. 3E and Fig. EV3C.**

*These mutations in MDA5 were previously described in: Wu et al., Structural basis for dsRNA recognition, filament formation, and antiviral signal activation by MDA5, Cell 2013.

As a side note, we did attempt to co-IP myc-MDA5 and FLAG-LGP2 from ADAR1-depleted cells. While we did find that myc-MDA5 and FLAG-LGP2 co-immunoprecipitate, we did not find a difference between ADAR1-sufficient and -deficient cells (Fig. 4A, reviewer only). This may be due to autoactivation following overexpression of MDA5. We failed to detect endogenous MDA5 in FLAG-LGP2 immunoprecipitates (Fig. 4B, reviewer only). We did not include these data in the manuscript.

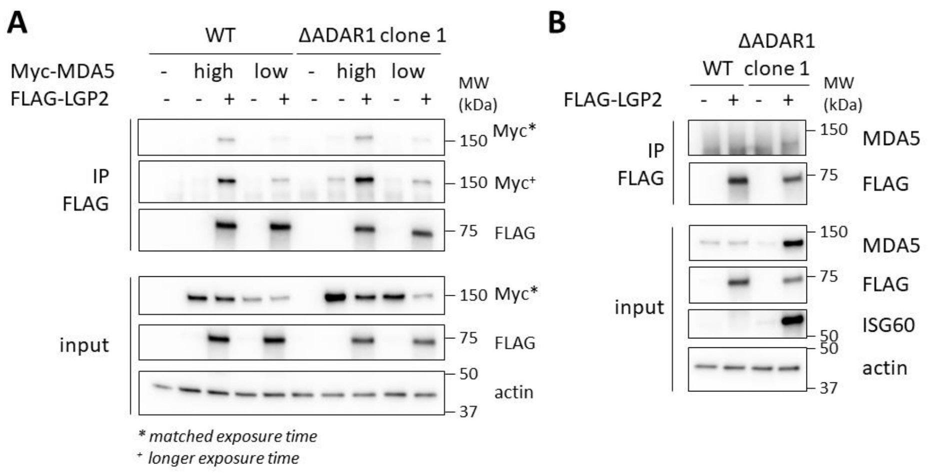

Fig. 4: **A**. Co-immunoprecipitation of FLAG-LGP2 and Myc-MDA5. FLAG-LGP2 and Myc-MDA5 were transiently expressed in WT or ADAR1 KO HEK293. LGP2 was immunoprecipitated through its FLAG tag. Immunoprecipitates were separated by SDS-PAGE and immunoblotted using the indicated antibodies. **B.** Co-immunoprecipitation of FLAG-LGP2 and *endogenous* MDA5. FLAG-LGP2 was transiently expressed in WT or ADAR1 KO HEK293 and LGP2 was immunoprecipitated through its FLAG tag. Immunoprecipitates were separated by SDS-PAGE and immunoblotted using the indicated antibodies.

Minor issues:

- LGP2 levels are increased in the presence of ADAR1 (see Fig. 2C, Fig. S3B, Fig. S5C-D), however this effect is not visible in S1D. Can the authors speculate why this may be the case?

The reviewer pointed out that *ectopic* LGP2 protein levels are increased in ADAR1-expressing cells, as compared to ADAR1-deficient cells (**Fig. 2C** and **Fig. EV3B**). We have looked further into this and observed that LGP2 mRNA levels are unaltered between ADAR1-sufficient and -deficient cells, i.e. the observed differences are evident at protein level but not at transcript level (Fig. 5A, reviewer only). This suggests that the increase in FLAG-LGP2 expression is not simply due to differences in plasmid transfection efficiency. We subsequently tested whether increased FLAG-LGP2 expression depends on ADAR1 expression *per se* or on its editing activity. We find that FLAG-LGP2 protein (but not mRNA) levels are also elevated in the presence of an ADAR1-editing deficient mutant (see Fig. 5B, reviewer only). A possible explanation is that the LGP2-dependent type I IFN response that is induced in the absence of ADAR1 results in increased degradation of LGP2. Another explanation is that oligomerised LGP2 is not as efficiently retrieved from lysates or separated by SDS-PAGE. In either case, LGP2 is sufficiently expressed to elicit a type I IFN response in absence of ADAR1.

As a side note, the increase in ectopic LGP2 protein expression in the presence of ADAR1 is also seen in **Fig. EV1D**. This is better visible in Fig. 6 (reviewer only), in which the samples from **Fig. EV1D** were loaded in a different order that allows direct comparison between ADAR1-sufficient and -deficient cells.

Finally, **Fig. EV5B-C** shows an increase in *endogenous* LGP2 expression in the *absence* of ADAR1. This can be explained by its IFN-inducible nature (i.e. it is an ISG), which means it is elevated in response to loss of ADAR1.

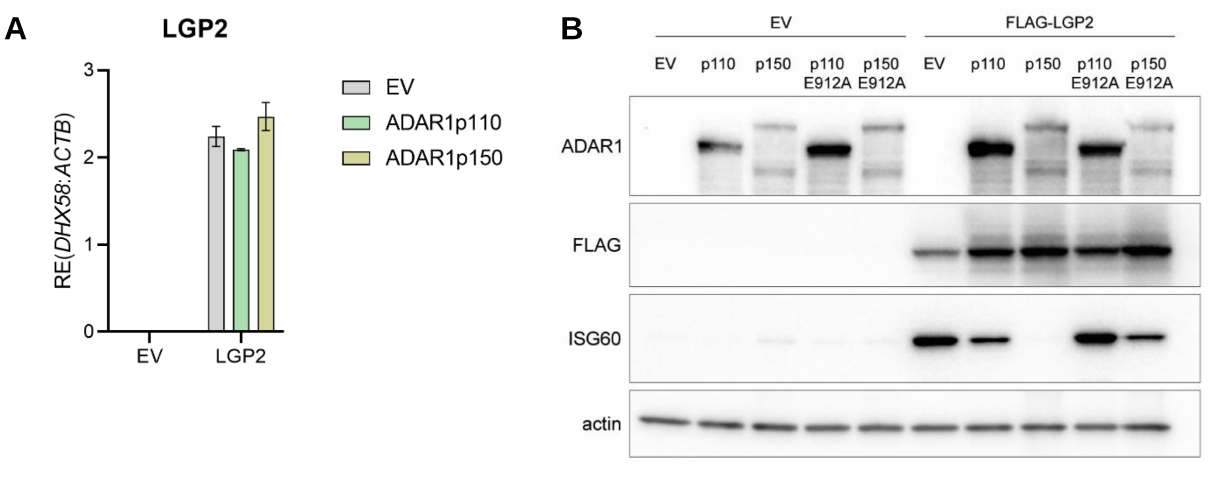

Fig. 5: **A.** FLAG-LGP2 mRNA expression is unaltered by the presence or absence of ADAR1 (RT-qPCR data are corresponding to data presented in **Fig. 2C**). **B.** FLAG-LGP2 protein expression is increased in ADAR1-expressing cells, irrespective of the editing activity of ADAR1.

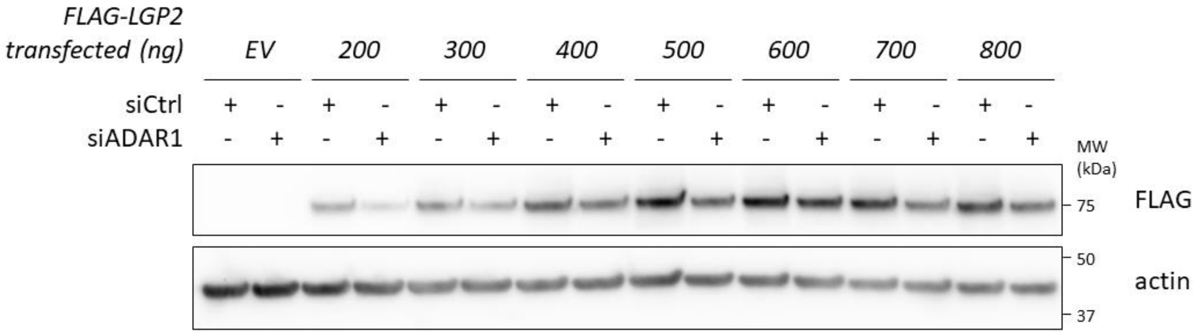

Fig. 6: Re-analysed samples of **Fig. EV1D**, now loading samples from ADAR1-sufficient and -deficient cells next to each other to allow direct comparison of FLAG-LGP2 expression.

- Fig S4: Please quantify the overall survival rate and hazard ratio for all selected cancers and calculate whether there is a significant difference between ADARlowDHX58high and ADARlowDHX58low patients. Can the statement from the manuscript (line 309, 'improved overall survival') be really made? Please rephrase to avoid overinterpretation.

The quantification of the overall survival rate and hazard ratio has already been included in **Fig. EV4A** (see legend on the bottom of the panel). To clarify further, we have now added the individual *p* values for all comparisons in **Fig. EV4A**.

In addition, we here provide all individual Kaplan Meier curves for all cancers examined (Fig. 7, reviewer only). We feel that this bulky dataset does not truly add any information that is not contained yet in **Fig. EV4A** and hence we excluded these plots from the manuscript.

We have rephrased the text as follows to avoid overstatement:

Line 315-320: 'Consistent with the hypothesis, *ADAR*^{low}*DHX58*^{high} patients had improved overall survival compared to *ADAR*^{low}*DHX58*^{low} patients in bladder cancer (BLCA), breast cancer (BRCA), sarcoma (SARC), esophageal (ESCA) and liver (LIHC) cancer (Fig. 5A and Supplemental Fig. 4). In contrast, no difference in survival was found for these cancer types in *ADAR*^{high} cancer patients stratified based on *DHX58* levels.'

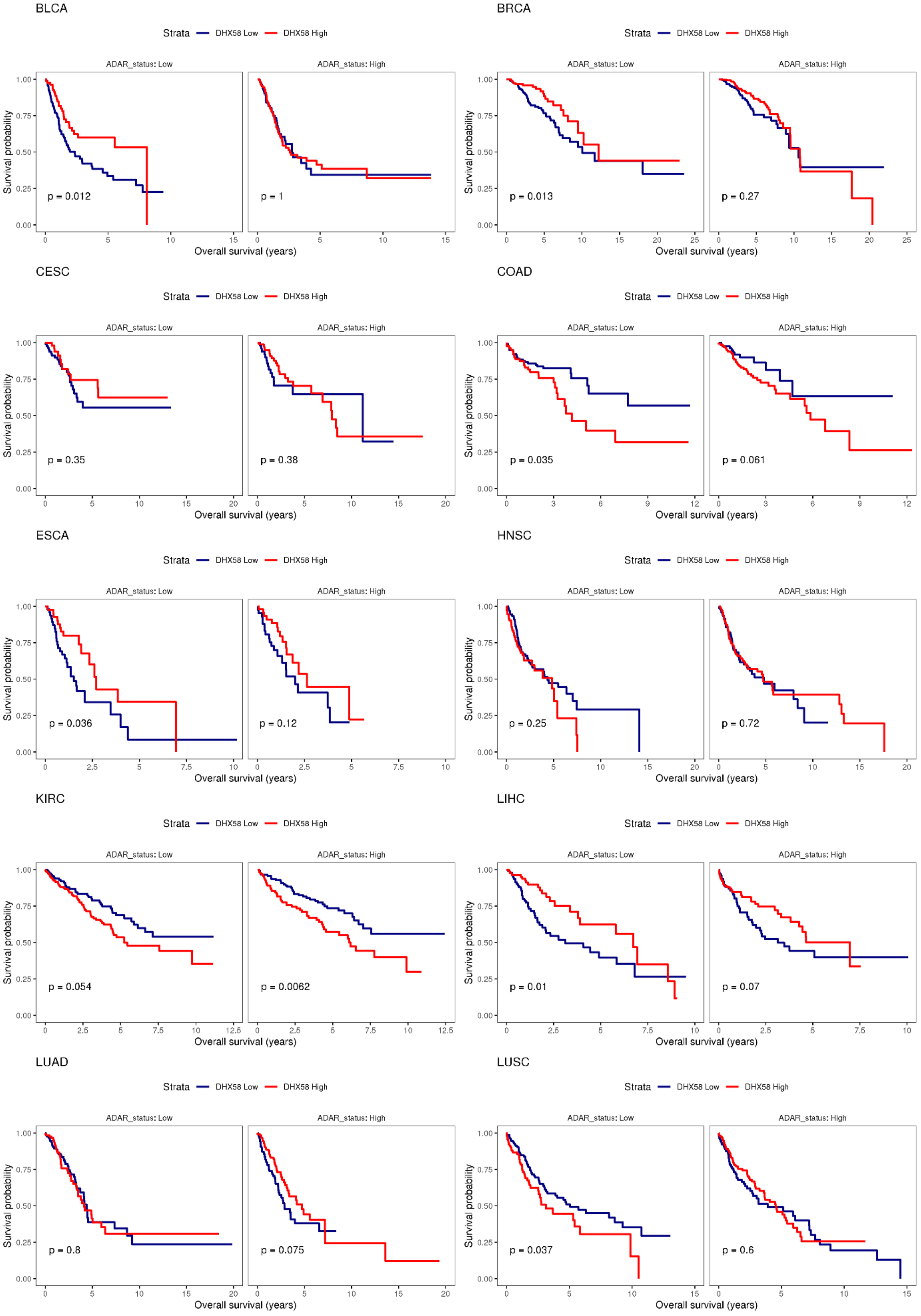
BLCA
Strata DHX58 Low DHX58 High
ADAR_status: Low
ADAR_status: High
Survival probability
p = 0.012
p = 1
Overall survival (years)
BRCA
p = 0.013
p = 0.27
CESC
p = 0.35
p = 0.38
COAD
p = 0.035
p = 0.061
ESCA
p = 0.036
p = 0.12
HNSC
p = 0.25
p = 0.72
KIRC
p = 0.054
p = 0.0062
LIHC
p = 0.01
p = 0.07
LUAD
p = 0.8
p = 0.075
LUSC
p = 0.037
p = 0.6

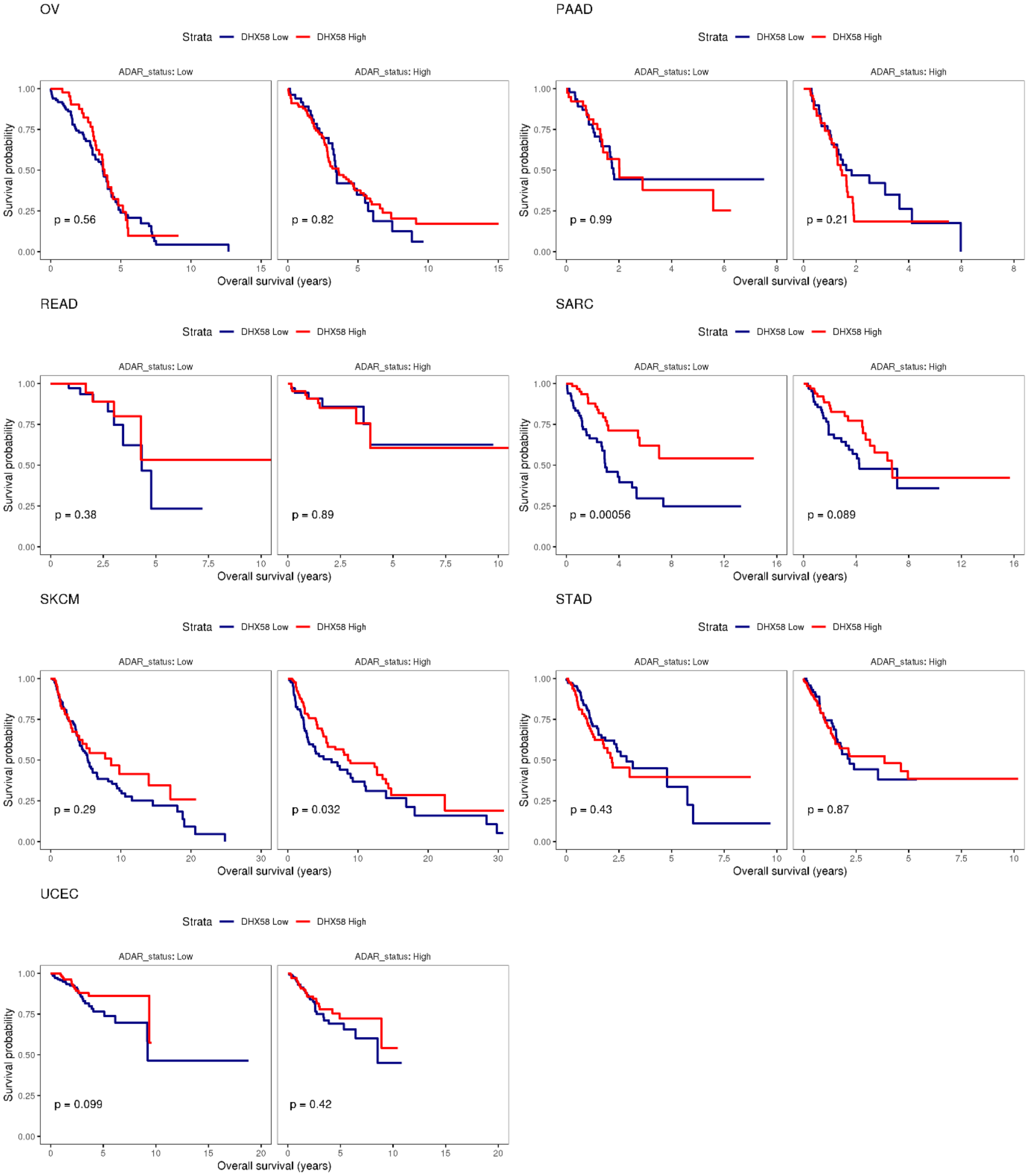

Fig. 7: Individual Kaplan-Meier plots from all cancers examined in **Fig. 5A** and **Fig. EV4A**. Sarcoma = SARC, liver = LIHC, esophageal = ESCA, breast = BRCA, bladder = BLCA, endometrial = UCEC, rectal = READ, cervical = CESC, melanoma = SKCM, ovarian = OV, pancreas = PAAD, lung adenocarcinoma = LUAD, stomach = STAD, head and neck = HNSC, clear cell renal cell carcinoma = KIRC, lung squamous = LUSC, colon = COAD). Median cut-off values for both *ADAR* and *DHX58* were used for patient stratification. Survival curves were compared using a log-rank test.

- Fig 4B+C: It seems like the Q390R/T395R mutants has an impact on type I IFN induction. In contrast, the manuscript states that the mutants has no impact on LGP2 ability to induce type I IFN induction (line 288). Please rephrase

The reviewer correctly pointed out that the Q390R/T395R has a modest impact on type I IFN induction, unlike the Q390A/Q394A mutant. This was consistently seen in our experiments (n=3). We have therefore rephrased the overstatement in the text as follows:

Line 293-296: “We found that the Q390A/Q394A mutation did not impact on the ability of LGP2 to induce a type I IFN response in ADAR1 knockout cells, while the Q390R/T395R mutation reduced, but not abolished, type I IFN induction (**Fig. 4B-C**).”

- Fig 4B: Please provide a corresponding expression blot

The corresponding expression blot is provided in **Fig. 4C**.
All samples were analysed on a single membrane. The dotted line indicates the juxtaposition of two non-adjacent lanes. We have clarified this in the corresponding figure legend.

- Fig S5C: Please add an si.LGP2 only control

This has been done and **Fig. EV5B** (previously Fig. S5C) has been replaced with a new panel that includes the siLGP2 only control.

Referee #3:
In this study by Stok et al, the authors investigate the role of LGP2 in recognizing self-RNA and inducing type I IFN in the absence of ADAR1. Previous studies demonstrate that in the absence of ADAR1, RNA sensor MDA5 recognizes self dsRNAs and activates the production of type I IFN, demonstrating that ADAR1 is important for preventing induction of innate immune responses through the recognition of self RNA (Alu elements). Concurrent deletion of MDA5/ADAR1 abrogates the induction of type I IFN by self dsRNA. A recent study demonstrates that loss of LGP2 rescues the embryonic lethality associated with the ADAR1 Za RNA binding mutant, indicating that LGP2 may be play a role in recognition of self RNA. Here the authors strengthen the role of LGP2 in recognizing self RNA and inducing the production of IFN through its interactions with MDA5. In addition, the authors demonstrate that the RNA binding and ATPase activities of LGP2 are critical for recognition of self RNA and induction of type I IFN. These are novel and interesting findings that demonstrate that higher type I IFN expression in the absence of ADAR1 is due to recognition of self RNA by LGP2 and not MDA5. There are some major concerns, as the majority of the data relies on an artificial overexpression of LGP2 in HEK 293 cells for the observed phenotype. The authors should address the following:

We thank the reviewer for his/her positive and constructive feedback. We are pleased to inform the reviewer that we have included new data in this revision on the validation of our observations in primary human macrophages (see details below). Our observations have now been demonstrated in THP1-derived macrophages, primary human macrophages, HEK293 cells, CAL27 cells, HT29 cells, and LIM1215 cells using either siRNA, shRNA or CRISPR KO approaches.

Major concerns:
1. The specificity of siRNA KD/CRIPSR KO should be demonstrated with cDNA complementation experiments.

Complementation of LGP2 KO HEK293 cells is shown in **Fig. EV2C and EV2D**.

Complementation of ADAR1 KO HEK293 cells is shown in **Fig. 2B and 2C and Fig. EV3A and EV3B**.

Complementation of MDA5 KO HEK293 cells is now shown in **Fig. 3E and Fig. EV3C** (both newly added).

We did not set-up complementation experiments of RIG-I KO HEK293 cells, as our data is consistent with published literature that demonstrates that RIG-I is not important for type I IFN induction upon loss of ADAR1 (reviewed in: Heraud-Farlow JE & Walkley CR, Journal of Molecular Medicine 2016). This is therefore not a key point in our study.

In THP1 cells (**Fig. 1A and 1B**), we have used two CRISPR clones for each RLR KO (generated using different gRNAs) to reduce the likelihood of off-target effects.

In addition to the above points, we have now also validated the role of LGP2 in type I IFN induction in ADAR1 deficiency in **primary human monocyte-derived macrophages**. These data are added as a new panel in **Fig. 1C** Note that these experiments were done with an independent set of siRNAs (ON-TARGETplus siRNA instead of siGenome siRNA, which are

used in **Fig. 1E-G**, **Fig. 5B-C** and **Fig. 6**). This experiment therefore further addresses the concerns about the specificity of the siRNAs.

2. The association of ADAR1/DHX58 in cancer survival is only a correlation and it needs to be functionally associated with induction of IFN through recognition of self RNA via LGP2. Also, the induction of IFN during treatment of ADAR1 KD cells with anticancer drugs can occur independently of the RLR pathway.

The association between *ADAR*/*DHX58* in cancer survival is indeed only a correlation, as indicated in the text (line 320-324). We have functionally associated *ADAR*, *DHX58*, cell growth, and type I IFN induction in **Fig. 5** and **Fig. 6** of our manuscript. Of course, these *in vitro* experiments do not reflect patient survival, yet do hint at a causal relationship between *ADAR*, *DHX58*, and survival. As pointed out in our discission (line 468-470), these observations would have to be further validated in *in vivo* models.

Furthermore, the reviewer pointed out that the induction of IFN upon treatment with anticancer drugs can also occur independently of the RLR pathway. We believe the reviewer is referring to Chiappinelli *et al*., Cell, 2015. This study demonstrated that treatment of ovarian cancer cells with 5-Azacytidine induced type I IFNs in a MAVS- and TLR3-dependent manner. This could indeed explain the residual type I IFN response that we observe in cells that are deficient in ADAR1 and LGP2 and treated with 5-Azacytidine (**Fig. 6A** and **6B**). We have now clarified this in our text through the following addition:

Line 356-357: "A possible explanation is that this LGP2-independent ligand activates TLR3 (Chiappinelli *et al*, 2015)."

3. This study can be strengthened by comparing the binding efficiencies of LGP2 vs MDA5 to self RNA.

This is an interesting suggestion. However, as the identity of the immunostimulatory self RNA in ADAR1-deficient cells remains poorly characterized, it is currently not possible to do such *in vitro* experiments. In our future research, we may use the model system that we have generated to identify the immunostimulatory RNAs that are associated with LGP2 in ADAR1-deficient cells using iCLIP. The suggested experiment on the binding efficiencies of LGP2 vs MDA5 will definitely be a relevant point to explore further once the ligand has been characterized. We therefore feel this suggestion is outside the scope of the current study.

4. As the current model still relies on MDA5 for induction of type I IFN, the authors should perform experiments in ADAR1/LGP2 double KO THP1 cells or another relevant cell line that does not require overexpression of LGP2.

We have additionally shown that type I IFN induction in ADAR1-depleted cells requires LPG2 in **primary human monocyte-derived macrophages** (see also major comment #1 of reviewer #2). These data are included as a new panel in **Fig. 1C**.

We have now demonstrated this phenotype in THP1-derived macrophages, HT29, LIM1215, and primary human macrophages using siRNAs, shRNAs, and CRISPR KOs targeting ADAR1 and/or LGP2. In none of these cell types, LGP2 was overexpressed. We feel that altogether this provides solid data on the role of LGP2 in type I IFN induction following ADAR1 depletion.

5. Is overexpression of RIG-I or MDA5 sufficient to induce IRF3 localization/IFN responses in the absence of ADAR1?

Yes, but overexpression of RIG-I or MDA5 will also induce IRF3 localization and a type I IFN response in the *presence* of ADAR1, due to autoactivation of these receptors when overexpressed.
As shown in **Fig. EV2A/B**, upregulation of *endogenous* MDA5 expression through pre-treatment of HEK293 with recombinant type I IFN was not sufficient to bypass LGP2 and to induce a type I IFN response in the absence of LGP2.

6. Is the RNA binding function of MDA5 required for LGP2 mediated induction of type I IFN?

This question was also raised by reviewer #2 and we have addressed this question experimentally. Please see major comment #2 of reviewer #2 for our response.

Minor concerns:
1. Statistical analysis should be included in all graphs.

Statistical analysis has now been included in all graphs in **Fig. 5** and **6**, according to EMBO Journal guidelines. **Fig. 5C** and **Fig. 6A-C** have therefore now changed to include all data (n=3) rather than a representative experiment.

2. Fig 1: KD efficiency of ADAR1 proteins in THP1 cells should be included.

We kindly refer the reviewer to reviewer #1, comment #3, where we have addressed this comment.

3. Fig 1F: There is translocation of IRF3 to the nucleus only in cells expressing low levels of LGP2 but not in high expressing cells. Why?

A possibility is that LGP2 is (partially) degraded in ADAR1-deficient cells as a mechanism to ‘shut off’ the type I IFN response in these cells. Alternatively, the epitope may be less accessible when LGP2 is activated and oligomerized. This would be consistent with the observation that FLAG-LGP2 expression is lower in ADAR1-deficient cells (**Fig. 2C** and **Fig. EV3B**; see also minor comment #6 below and reviewer #2, minor issue #1).

We have included a number of additional representative images below (Fig. 8, reviewer only). Perhaps there is a trend that low LGP2 expressors more frequently show nuclear IRF3 than high LGP2 expressors, but nuclear IRF3 is definitely not absent from all high LGP2 expressors (see for example the image in the bottom right in Fig. 8, reviewer only).

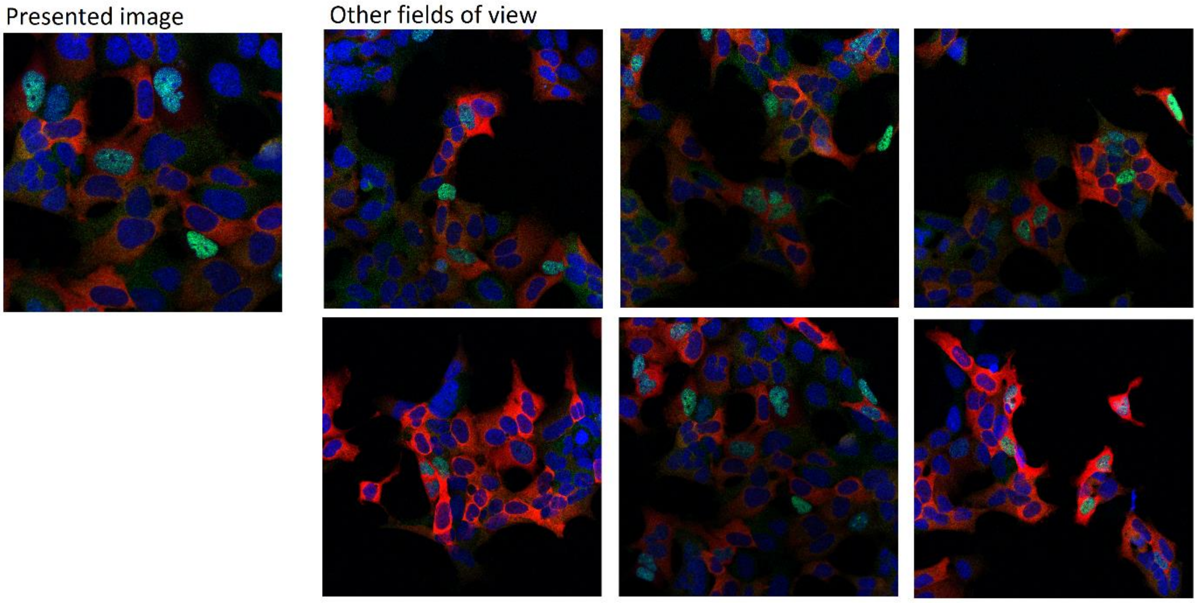

Fig. 8: Representative images belonging to **Fig. 1G**. Nuclear localization of IRF3 (green) in FLAG-LGP2 (red) expressing HEK293 upon ADAR1 depletion.

4. Fig S1A: Induction of ISG60 is low in 293 cells as compared to the data shown in Fig 1C.

This is due to a difference in exposure time of the images shown. In **Fig. EV1A** the exposure time was chosen based on the expression level of ISG60 in THP-1 cells, in which the signal intensity of ISG60 was much stronger. In **Fig. 1D (**previously Fig. 1C**)**, ISG60 is only shown in HEK293 cells and the exposure time was longer. In Fig. 9 (reviewer only) we show 'matched' exposure times.

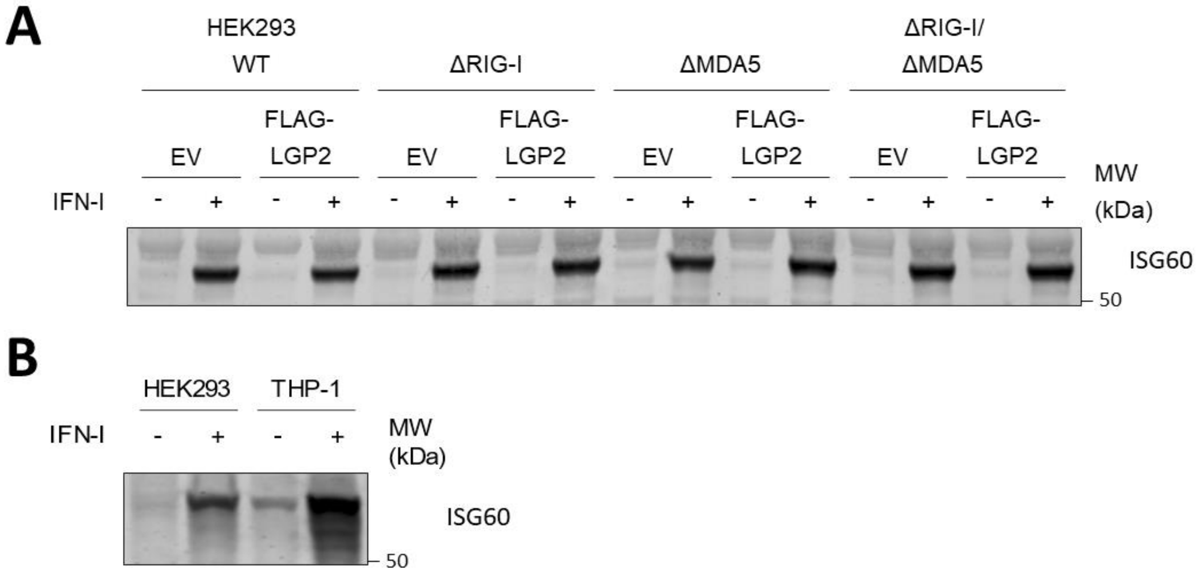

Fig. 9: Equal exposures ('matched exposures') of the ISG60 stains shown in **Fig 1D** (**A**) and **Fig. EV1A** (**B**).

5. Fig S2A: Wt controls are missing for IFN treatment groups. Along with IRF3 target genes, Stat1/2 regulated genes should be included as controls for ISG induction in response to IFN treatment.

We have now included WT controls in **Fig. EV2A and EV2B**.

Upregulation of Stat1/2-regulated genes (i.e. ISGs) is shown in **Fig. EV2B** (MDA5, and the newly added ISG60).

6. Fig 2C: FLAG-LGP2 expression is lower than other controls.

This point was also raised by reviewer #2 (minor issue #1). We have addressed this comment experimentally; see reviewer #2, minor issue #1.

7. LN172-174: It is not clear what the authors mean by "monitoring p150 RNA levels leads to distorted results".

We have rephrased the text as follows:

Line 171-174: "Note that throughout the manuscript ADAR1 knockdown efficiency is monitored through measurement of p110 expression levels, as analysis of the p150 isoform underestimates knockdown efficiency due to its IFN-inducible nature."

Basically, p150 (in contrast to p110) is and IFN-stimulated gene, hence the type I IFN response that is induced upon depletion of ADAR1 leads to a rapid increase in p150 expression levels. The depletion of p150 is therefore overcome quicker than usually seen in an siRNA experiment.

8. Fig 5B must include some sort of quantitative measurement rather than measurement of crystal violet staining intensity.

We have quantified the Crystal Violet staining by extraction of the Crystal Violet dye from the samples and analysis of absorbance on a spectrophotometer. We have included statistics on these measurements in **Fig. 5C** (n=3).
We have now also analysed cell growth over time of ADAR1-depleted CAL27 cells (-/+ LGP2) on an Incucyte live cell imager. These data were in line with the data shown in **Fig. 5B-C** (Crystal Violet assays), and are now included as a new panel in **Fig. EV4B-D**.

1st Revision - Editorial Decision 5th Jan 2022

Dear Annemarthe,

Thank you for submitting your revised manuscript to The EMBO Journal. Your revised version has now been seen by the original referees and their comments are provided below.

As you can see from the comments, the referees appreciate the introduced changes and support publication here. I agree with the comments raised by referee #1 that it would be good to include the data provided "reveiwer-only" Fig 1 and Fig 3 to the manuscript. Let's discuss if it would be useful to include the other figures as well. Please also discuss the caveats and limitation of the conclusions as indicated by referee #1.

When you submit your revised manuscript will you alsotake care of the following editorial points.

- you can only have 5 keywords (you have 6)

- the Data Availability section is missing. This is the place to enter accession numbers of newly generated data. This section should be placed after M&M and before Acknowledgements. If no new data is generated that needs to be deposited in a database then state: This study includes no data deposited in external repositories.

- the funding "Institute for Chemical Immunology (ICI-00203)" is missing from the online submission system

- M&M should be after Discussion.

- Table EV1 and EV2 should be renamed Table 1 and Table 2. Please correct callout in text as well.

- Our publisher has also done their pre-publication check on your manuscript. When you log into the manuscript submission system you will see the file "Data Edited Manuscript file". Please take a look at the word file and the comments regarding the figure legends and respond to the issues.

- Regarding statistic: There are several places where N=2 or N=1. We generally avoid showing statistics when N<3. Can you simply show the data points?

- We encourage the publication of source data, particularly for electrophoretic gels and blots, with the aim of making primary data more accessible and transparent to the reader. It would be great if you could provide me with a PDF file per figure that contains the original, uncropped and unprocessed scans of all or key gels used in the figure? The PDF files should be labeled with the appropriate figure/panel number, and should have molecular weight markers; further annotation could be useful but is not essential. The PDF files will be published online with the article as supplementary "Source Data" files.

- We include a synopsis of the paper (see http://emboj.embopress.org/). Please provide me with a general summary statement and 3-5 bullet points that capture the key findings of the paper.

- We also need a summary figure for the synopsis. The size should be 550 wide by [200-400] high (pixels). You can also use something from the figures if that is easier.

That should be all. Let me know if we need to discuss anything further.

With best wishes

Karin

Karin Dumstrei, PhD
Senior Editor
The EMBO Journal

Instructions for preparing your revised manuscript:

Guide For Authors: https://www.embopress.org/page/journal/14602075/authorguide

Use the link below to submit your revision:

https://emboj.msubmit.net/cgi-bin/main.plex

Referee #1:

The authors did a reasonable job answering my questions using new data, however have decided not to include most of the data in the manuscript. This is despite the fact that the new "reviewer-only" data should modify the conclusion in a way that they help the readers understand the nuanced aspect of the experiments. I strongly suggest that the "reveiwer-only" data in Fig 1 and Fig 3 included in the manuscript and the authors discuss caveats and limitation of their experiment/conclusion.

Referee #2:

The authors have addressed all my suggestions and comments perfectly and the updated manuscript is much stronger. It was a pleasure to read it and look at the exciting data!

Referee #3:

The authors have reasonably addressed all of my previous concerns.

Rebuttal letter

Response to editorial comments:

Note: in addition to the points below we have now shortened our abstract to 175 words.

- you can only have 5 keywords (you have 6)

We have deleted the word 'self RNA sensing'.

- the Data Availability section is missing. This is the place to enter accession numbers of newly generated data. This section should be placed after M&M and before Acknowledgements. If no new data is generated that needs to be deposited in a database then state: This study includes no data deposited in external repositories.

We have incorporated the statement 'This study includes no data deposited in external repositories' in the M&M.

- the funding "Institute for Chemical Immunology (ICI-00203)" is missing from the online submission system

Corrected.

- M&M should be after Discussion.

The M&M is now placed directly after the Discussion.

- Table EV1 and EV2 should be renamed Table 1 and Table 2. Please correct callout in text as well.

Corrected.

- Our publisher has also done their pre-publication check on your manuscript. When you log into the manuscript submission system you will see the file "Data Edited Manuscript file". Please take a look at the word file and the comments regarding the figure legends and respond to the issues.

The comments of Vivian Killet have been addressed: we have incorporated all her corrections and suggestions in the figure legends of the revised manuscript.

- Regarding statistic: There are several places where N=2 or N=1. We generally avoid showing statistics when N<3. Can you simply show the data points?

The error bars in qPCR experiments refer to technical replicates, with the exception of Fig. 1C and Fig. 6. The N number referred to the number of biological replicates. We have clarified the figures legends as suggested by Vivian Killet. The figure legends now include the following statement: "Data are means ± s.d. from a representative of three biological replicate experiments". In the M&M we have specified that for all qPCR experiments technical duplicates or triplicates were analysed.

- We encourage the publication of source data, particularly for electrophoretic gels and blots, with the aim of making primary data more accessible and transparent to the reader. It would be great if you could provide me with a PDF file per figure that contains the original, uncropped and unprocessed scans of all or key gels used in the figure? The PDF files should be labeled with the

appropriate figure/panel number, and should have molecular weight markers; further annotation could be useful but is not essential. The PDF files will be published online with the article as supplementary "Source Data" files.

All source data for gels and blots have been collected in PDF file and is ready for publication as Source Data.

- We include a synopsis of the paper (see http://emboj.embopress.org/). Please provide me with a general summary statement and 3-5 bullet points that capture the key findings of the paper.

A synopsis of the paper has been uploaded in the submission system.

- We also need a summary figure for the synopsis. The size should be 550 wide by [200-400] high (pixels). You can also use something from the figures if that is easier.

A summary figure has been uploaded in the submission system.

Response to referee comments:

Referee #1:

The authors did a reasonable job answering my questions using new data, however have decided not to include most of the data in the manuscript. This is despite the fact that the new "reviewer-only" data should modify the conclusion in a way that they help the readers understand the nuanced aspect of the experiments. I strongly suggest that the "reveiwer-only" data in Fig 1 and Fig 3 included in the manuscript and the authors discuss caveats and limitation of their experiment/conclusion.

We have incorporated the 'reviewer only' data from Fig. 3 as new Figure EV1A and refer to this figure in the main text (line 189-190), pointing out the modest knockdown efficiency of ADAR1 in THP1. In addition, we have added the 'reviewer only' data Fig. 1 as new Figure EV2G and discuss the implications of this experiment in line 256-259 as follows: "As a side note, in another setting of autoinflammation due to a gain-of-function mutation in MDA5 (MDA5 G495R), LGP2 also enhanced but was not strictly required for type I IFN induction (Fig. EV2G), which indicates that LGP2 is not necessarily essential for the detection of all types of self RNA."
We hope that this will further clarify the findings for the readers.

Referee #2:

The authors have addressed all my suggestions and comments perfectly and the updated manuscript is much stronger. It was a pleasure to read it and look at the exciting data!

Referee #3:

The authors have reasonably addressed all of my previous concerns.

2nd Revision - Editorial Decision 11th Jan 2022

Dear Annemarthe,

Thank you for submitting by your revised manuscript to The EMBO Journal. I have now had a chance to take a look at the revised manuscript and all looks good.

I am therefore very pleased to accept the manuscript for publication here.

Congratulations on a nice study

With best wishes

Karin

Karin Dumstrei, PhD
Senior Editor
The EMBO Journal

Please note that it is EMBO Journal policy for the transcript of the editorial process (containing referee reports and your response letter) to be published as an online supplement to each paper. If you do NOT want this, you will need to inform the Editorial Office via email immediately. More information is available here:
https://www.embopress.org/page/journal/14602075/authorguide#transparentprocess

Your manuscript will be processed for publication in the journal by EMBO Press. Manuscripts in the PDF and electronic editions of The EMBO Journal will be copy edited, and you will be provided with page proofs prior to publication. Please note that supplementary information is not included in the proofs.

Please note that you will be contacted by Wiley Author Services to complete licensing and payment information. The 'Page Charges Authorization Form' is available here: https://www.embopress.org/pb-assets/embo-site/tej_apc.pdf

Should you be planning a Press Release on your article, please get in contact with embojournal@wiley.com as early as possible, in order to coordinate publication and release dates.

If you have any questions, please do not hesitate to call or email the Editorial Office. Thank you for your contribution to The EMBO Journal.

** Click here to be directed to your login page: https://emboj.msubmit.net

EMBO PRESS

YOU MUST COMPLETE ALL CELLS WITH A PINK BACKGROUND

PLEASE NOTE THAT THIS CHECKLIST WILL BE PUBLISHED ALONGSIDE YOUR PAPER

Corresponding Author Name: Dr. A.G. van der Veen

Journal Submitted to: The EMBO Journal
Manuscript Number: EMBOJ-2021-109760

USEFUL LINKS FOR COMPLETING THIS FORM

http://www.antibodypedia.com
http://1degreebio.org
http://www.equator-network.org/reporting-guidelines/improving-bioscience-research-repor
http://grants.nih.gov/grants/olaw/olaw.htm
http://www.mrc.ac.uk/Ourresearch/Ethicsresearchguidance/Useofanimals/index.htm
http://ClinicalTrials.gov
http://www.consort-statement.org
http://www.consort-statement.org/checklists/view/32-consort/66-title
http://www.equator-network.org/reporting-guidelines/reporting-recommendations-for-tumo
http://datadryad.org
http://figshare.com
http://www.ncbi.nlm.nih.gov/gap
http://www.ebi.ac.uk/ega
http://biomodels.net/
http://biomodels.net/miriam/
http://jjj.biochem.sun.ac.za
https://osp.od.nih.gov/biosafety-biosecurity-and-emerging-biotechnology/
http://www.selectagents.gov/

Reporting Checklist For Life Sciences Articles (Rev. June 2017)

This checklist is used to ensure good reporting standards and to improve the reproducibility of published results. These guidelines are consistent with the Principles and Guidelines for Reporting Preclinical Research issued by the NIH in 2014. Please follow the journal's authorship guidelines in preparing your manuscript.

A- Figures
1. Data
The data shown in figures should satisfy the following conditions:

- the data were obtained and processed according to the field's best practice and are presented to reflect the results of the experiments in an accurate and unbiased manner.
- figure panels include only data points, measurements or observations that can be compared to each other in a scientifically meaningful way.
- graphs include clearly labeled error bars for independent experiments and sample sizes. Unless justified, error bars should not be shown for technical replicates.
- if n< 5, the individual data points from each experiment should be plotted and any statistical test employed should be justified
- Source Data should be included to report the data underlying graphs. Please follow the guidelines set out in the author ship guidelines on Data Presentation.

2. Captions
Each figure caption should contain the following information, for each panel where they are relevant:

- a specification of the experimental system investigated (eg cell line, species name).
- the assay(s) and method(s) used to carry out the reported observations and measurements
- an explicit mention of the biological and chemical entity(ies) that are being measured.
- an explicit mention of the biological and chemical entity(ies) that are altered/varied/perturbed in a controlled manner.

- the exact sample size (n) for each experimental group/condition, given as a number, not a range;
- a description of the sample collection allowing the reader to understand whether the samples represent technical or biological replicates (including how many animals, litters, cultures, etc.).
- a statement of how many times the experiment shown was independently replicated in the laboratory.
- definitions of statistical methods and measures:
  - common tests, such as t-test (please specify whether paired vs. unpaired), simple $\chi 2$ tests, Wilcoxon and Mann-Whitney tests, can be unambiguously identified by name only, but more complex techniques should be described in the methods section;
  - are tests one-sided or two-sided?
  - are there adjustments for multiple comparisons?
  - exact statistical test results, e.g., P values = x but not P values < x;
  - definition of 'center values' as median or average;
  - definition of error bars as s.d. or s.e.m.

Any descriptions too long for the figure legend should be included in the methods section and/or with the source data.

In the pink boxes below, please ensure that the answers to the following questions are reported in the manuscript itself. Every question should be answered. If the question is not relevant to your research, please write NA (non applicable). We encourage you to include a specific subsection in the methods section for statistics, reagents, animal models and human subjects.

B- Statistics and general methods

Please fill out these boxes (Do not worry if you cannot see all your text once you press return)

1.a. How was the sample size chosen to ensure adequate power to detect a pre-specified effect size?	Image acquisition was performed on multiple fields of view (>10) to ensure enough cells can be analyzed for nuclear IRF3 translocation (Fig 1G and 3C) or to accurately determine cell confluency (Fig EV4C). For other experimental settings, independent repeats were performed to confirm phenotypes. The number or independent repeat experiments are indicated in the individual figure legends.
1.b. For animal studies, include a statement about sample size estimate even if no statistical methods were used.	NA
2. Describe inclusion/exclusion criteria if samples or animals were excluded from the analysis. Were the criteria pre-established?	Technical replicate values with a clearly abnormal melting curve were excluded from qRT-PCR analysis.
3. Were any steps taken to minimize the effects of subjective bias when allocating animals/samples to treatment (e.g. randomization procedure)? If yes, please describe.	NA
For animal studies, include a statement about randomization even if no randomization was used.	NA
4.a. Were any steps taken to minimize the effects of subjective bias during group allocation or/and when assessing results (e.g. blinding of the investigator)? If yes please describe.	NA
4.b. For animal studies, include a statement about blinding even if no blinding was done	NA
5. For every figure, are statistical tests justified as appropriate?	Yes, information on statistical tests is included in the figure legends and/or materials en methods section of the manuscript.
Do the data meet the assumptions of the tests (e.g., normal distribution)? Describe any methods used to assess it.	Data distribution was assessed for normality using D'Agostino-Pearson and Shapiro-Wilk normality tests, as mentioned in the materials and methods section. Non-normally distributed data were analysed using an appropiate nonparametric statistical tests, or in case of multiple-group analysis, data was log-transformed prior to two-way ANOVA analysis.

Is there an estimate of variation within each group of data?	NA
Is the variance similar between the groups that are being statistically compared?	Data for fig 5C do meet the equal variance assumption, whereas the data for fig 1G, 3C, and 6A-C do not. Appropiate statistical tests were used to account for unequal variances.

C- Reagents

6. To show that antibodies were profiled for use in the system under study (assay and species), provide a citation, catalog number and/or clone number, supplementary information or reference to an antibody validation profile. e.g., Antibodypedia (see link list at top right), 1DegreeBio (see link list at top right).	Antibody clone numbers and company information is provided in the Materials and Methods section as Supplemental Table 2.
7. Identify the source of cell lines and report if they were recently authenticated (e.g., by STR profiling) and tested for mycoplasma contamination.	HEK293: ATCC; HEK293T: ATCC; THP-1 Dual: Invivogen; CAL27: ATCC; LIM1215: Gift from René Bernards, The Netherlands Cancer Institute; HT29: Gift from Jacques van Dongen, Leids Universitair Medisch Centrum. Cell lines have been regularly tested negative for mycoplasma contamination. HEK293 cells were recently authenthicated by STR profiling.

* for all hyperlinks, please see the table at the top right of the document

D- Animal Models

8. Report species, strain, gender, age of animals and genetic modification status where applicable. Please detail housing and husbandry conditions and the source of animals.	NA
9. For experiments involving live vertebrates, include a statement of compliance with ethical regulations and identify the committee(s) approving the experiments.	NA
10. We recommend consulting the ARRIVE guidelines (see link list at top right) (PLoS Biol. 8(6), e1000412, 2010) to ensure that other relevant aspects of animal studies are adequately reported. See author guidelines, under 'Reporting Guidelines'. See also: NIH (see link list at top right) and MRC (see link list at top right) recommendations. Please confirm compliance.	NA

E- Human Subjects

11. Identify the committee(s) approving the study protocol.	Sanquin's internal ethical board (the Dutch blood bank)
12. Include a statement confirming that informed consent was obtained from all subjects and that the experiments conformed to the principles set out in the WMA Declaration of Helsinki and the Department of Health and Human Services Belmont Report.	The following statement is included in the materials and methods section: "Human materials were obtained in accordance with the Declaration of Helsinki and the Dutch rules with respect to the use of human materials from volunteer donors. Buffy coats from healthy anonymized donors were obtained after their written informed consent, as approved by Sanquin's internal ethical board. "
13. For publication of patient photos, include a statement confirming that consent to publish was obtained.	NA
14. Report any restrictions on the availability (and/or on the use) of human data or samples.	NA
15. Report the clinical trial registration number (at ClinicalTrials.gov or equivalent), where applicable.	NA
16. For phase II and III randomized controlled trials, please refer to the CONSORT flow diagram (see link list at top right) and submit the CONSORT checklist (see link list at top right) with your submission. See author guidelines, under 'Reporting Guidelines'. Please confirm you have submitted this list.	NA
17. For tumor marker prognostic studies, we recommend that you follow the REMARK reporting guidelines (see link list at top right). See author guidelines, under 'Reporting Guidelines'. Please confirm you have followed these guidelines.	NA

F- Data Accessibility

18: Provide a "Data Availability" section at the end of the Materials & Methods, listing the accession codes for data generated in this study and deposited in a public database (e.g. RNA-Seq data: Gene Expression Omnibus GSE39462, Proteomics data: PRIDE PXD000208 etc.) Please refer to our author guidelines for 'Data Deposition'.<br><br>Data deposition in a public repository is mandatory for:<br>a. Protein, DNA and RNA sequences<br>b. Macromolecular structures<br>c. Crystallographic data for small molecules<br>d. Functional genomics data<br>e. Proteomics and molecular interactions	NA
19. Deposition is strongly recommended for any datasets that are central and integral to the study; please consider the journal's data policy. If no structured public repository exists for a given data type, we encourage the provision of datasets in the manuscript as a Supplementary Document (see author guidelines under 'Expanded View' or in unstructured repositories such as Dryad (see link list at top right) or Figshare (see link list at top right).	NA
20. Access to human clinical and genomic datasets should be provided with as few restrictions as possible while respecting ethical obligations to the patients and relevant medical and legal issues. If practically possible and compatible with the individual consent agreement used in the study, such data should be deposited in one of the major public access-controlled repositories such as dbGAP (see link list at top right) or EGA (see link list at top right).	NA
21. Computational models that are central and integral to a study should be shared without restrictions and provided in a machine-readable form. The relevant accession numbers or links should be provided. When possible, standardized format (SBML, CellML) should be used instead of scripts (e.g. MATLAB). Authors are strongly encouraged to follow the MIRIAM guidelines (see link list at top right) and deposit their model in a public database such as Biomodels (see link list at top right) or JWS Online (see link list at top right). If computer source code is provided with the paper, it should be deposited in a public repository or included in supplementary information.	NA

G- Dual use research of concern

22. Could your study fall under dual use research restrictions? Please check biosecurity documents (see link list at top right) and list of select agents and toxins (APHIS/CDC) (see link list at top right). According to our biosecurity guidelines, provide a statement only if it could.	No
